# Streamlining NMR Chemical Shift Predictions for Intrinsically Disordered Proteins: Design of Ensembles with Dimensionality Reduction and Clustering

**DOI:** 10.1021/acs.jcim.4c00809

**Published:** 2024-08-05

**Authors:** Michael J. Bakker, Amina Gaffour, Martin Juhás, Vojtěch Zapletal, Jakub Stošek, Lars A. Bratholm, Jana Pavlíková Přecechtělová

**Affiliations:** † Faculty of Pharmacy in Hradec Králové, Charles University, Akademika Heyrovského 1203/8, 500 05 Hradec Králové, Czech Republic; ‡ Department of Chemistry, Faculty of Science, University of Hradec Králové, Rokitanského 62, 500 03 Hradec Králové, Czech Republic; ¶ Department of Chemistry, Faculty of Science, Masaryk University, Kotlářská 2, 611 37 Brno, Czech Republic; § School of Chemistry, University of Bristol, Cantock’s Close, BS8 1TS Bristol, U.K.

## Abstract

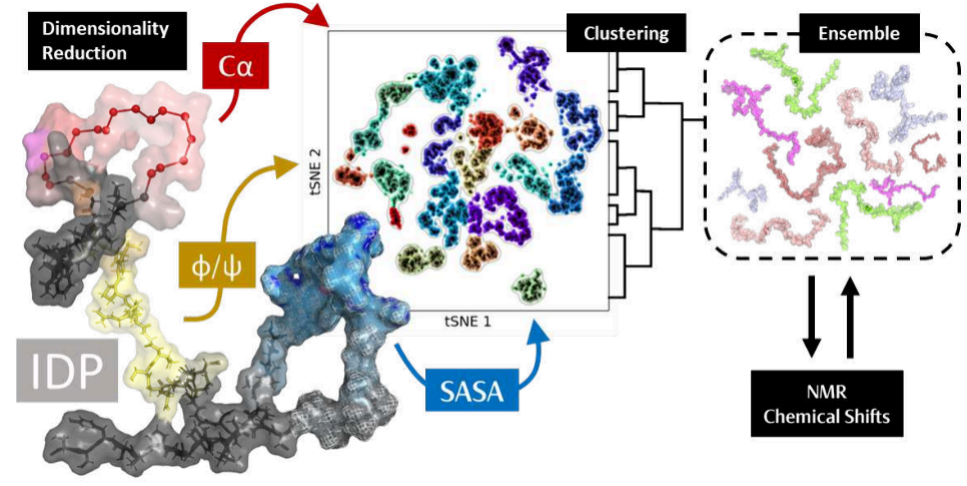

By merging advanced dimensionality reduction (DR) and clustering algorithm (CA) techniques, our study advances the sampling procedure for predicting NMR chemical shifts (CS) in intrinsically disordered proteins (IDPs), making a significant leap forward in the field of protein analysis/modeling. We enhance NMR CS sampling by generating clustered ensembles that accurately reflect the different properties and phenomena encapsulated by the IDP trajectories. This investigation critically assessed different rapid CS predictors, both neural network (e.g., Sparta+ and ShiftX2) and database-driven (ProCS-15), and highlighted the need for more advanced quantum calculations and the subsequent need for more tractable-sized conformational ensembles. Although neural network CS predictors outperformed ProCS-15 for all atoms, all tools showed poor agreement with H_N_ CSs, and the neural network CS predictors were unable to capture the influence of phosphorylated residues, highly relevant for IDPs. This study also addressed the limitations of using direct clustering with collective variables, such as the widespread implementation of the GROMOS algorithm. Clustered ensembles (CEs) produced by this algorithm showed poor performance with chemical shifts compared to sequential ensembles (SEs) of similar size. Instead, we implement a multiscale DR and CA approach and explore the challenges and limitations of applying these algorithms to obtain more robust and tractable CEs. The novel feature of this investigation is the use of solvent-accessible surface area (SASA) as one of the fingerprints for DR alongside previously investigated α carbon distance/angles or ϕ/ψ dihedral angles. The ensembles produced with SASA tSNE DR produced CEs better aligned with the experimental CS of between 0.17 and 0.36 r^2^ (0.18–0.26 ppm) depending on the system and replicate. Furthermore, this technique produced CEs with better agreement than traditional SEs in 85.7% of all ensemble sizes. This study investigates the quality of ensembles produced based on different input features, comparing latent spaces produced by linear vs nonlinear DR techniques and a novel integrated silhouette score scanning protocol for tSNE DR.

## Introduction

The biochemistry dogma suggests that proteins comprise amino acids that fold into 3D structures and determine the function of the protein. A growing field of research on the dark proteome reveals the importance of intrinsically disordered proteins (IDPs), which lack rigid secondary structures, both for their functions and prospective dysfunctions.^1−3^ This class of proteins comprises a significant portion of the human proteome^2,4−10^ and plays a crucial role in the regulation of cellular processes including signal transduction,^7,8,11^ transcription,^2,7,8,11,12^ and DNA repair.^2,5,13^ They are also involved in protein–protein interactions,^2,4,6−8,11,12^ acting as flexible scaffolds for enzymes,^2,4,7,9,11,13^ and binding to a range of partners with high affinity.^4,10,12^ In addition, IDPs are also responsible for the regulation of gene expression,^5,9,12,14,15^ cell differentiation,^9,16,17^ and the immune response,^18,19^ as they can adapt and respond rapidly to changes in their environment. These proteins are commonly associated with multiple disorders such as Alzheimer’s^5−9,15,19−21^ and Parkinson’s^5,7,11,19−21^ and are of great interest for the investigation of these disorders.

Many IDPs undergo post-translational modification, such as phosphorylation, which involves adding a phosphate group to a protein (Figure S1).^5,22^ This reversible process mediated by kinase enzymes can elicit diverse changes in the protein’s function, structure, or conformation.^23,24^ Phosphorylation can also disrupt or create new binding sites, alter protein stability, modulate precise cellular signaling pathways, and enable rapid responses to environmental stimuli.^4−9,11,13,15−21,23,24^ While extensive research has been conducted on the impact of phosphorylation on IDPs, the exact mechanisms are many times left unexplored. This is partially due to the technical challenges of characterizing and detecting these proteins.^25,26^

The functional role of IDPs and IDRs in the cell is predominantly based on their intrinsic flexibility and capability to perform various operations.^27,28^ This makes it particularly challenging to determine their structure using traditional experimental techniques, such as CryoEM, which only derives static structures.^29^ Utilizing mathematical algorithms, molecular dynamics (MD) can forecast protein behavior over time, providing insight into its dynamics, flexibility, and interactions with other molecules. In combination with NMR chemical shifts (CSs), relaxation data, small-angle scattering (SAXS), circular dichroism (CD), paramagnetic relaxation enhancements (PREs), or any other comparable experimental methods, MD provides a more comprehensive understanding of the structure and behavior of the protein.^28,30^

NMR CSs can be predicted or computed from the conformations of the proteins and averaged together to compare with experimental data. Quantum calculations can derive them,^31,32^ although computational resources required to compute chemical shielding for so many residues are daunting. Rapid neural network-derived chemical shift prediction (CSP) tools, such as Sparta+^33^ or ShiftX2,^34^ have emerged in recent decades to assist researchers in protein structure characterization. CSP models can be beneficial for ordered proteins, but these tools may be overfit for the concerns of IDPs. Other techniques have been implemented using databases of quantum chemical calculations (ProCS-15) to avoid complications from overfitting and to compute atoms outside the range of the backbone rapidly, unusual atom types (e.g., ^31^P), or without massive databases for the neural network to train upon.^35^

**Figure 1:**
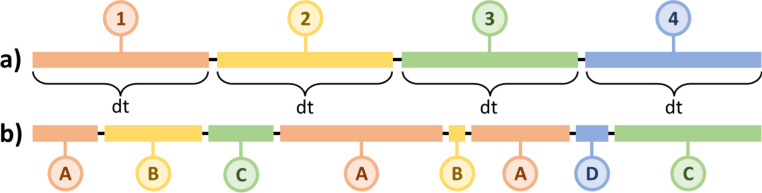
Representative schematic comparing conformational ensembles generated (a) sequentially and (b) by clustering.

While the application of CSP tools is computationally cheap and can be easily implemented on a complete MD simulation, calculating NMR parameters at the quantum mechanics level is significantly more demanding. State-of-the-art protein NMR calculations^31,36−38^ that build on MD and density functional theory (DFT) typically employ sequential structural ensembles (SEs), see Figure 1a. Biomolecular structures are taken from an MD simulation trajectory at regular time intervals, and the structural ensemble obtained is subject to follow-up NMR calculations.^39^ The computed NMR parameters are then averaged over the ensemble to mimic the time average observed in the experiment.

While such an approach gives reliable predictions, it often requires hundreds of structures^40^ in the computed ensemble to obtain converged ensemble averages. The number of calculations that must be performed is given by (number of structures in an ensemble) × (number of residues),^31,36^ leading to a fast consumption of high-performance computing resources. The computational costs become a severe drawback, especially if the DFT calculations are repeated using a different ensemble, model chemistry, or fragment/surrounding size.^36^ The problem can be tackled by replacing the sequential ensemble with a cluster-based ensemble (CE), see Figure 1b. Cluster analysis (CA)^41−43^ is applied to an MD simulation trajectory to identify clusters of similar structures, and structures representative of individual clusters constitute an ensemble for NMR calculations.^31^ The approach has only been used sparsely and is typically employed by clustering based on an RMSD, which works reasonably well for biomolecules with a well-defined secondary and tertiary structure, such as DNA and structured proteins.

Applying RMSD-based clustering to highly flexible intrinsically disordered proteins (IDPs) is problematic.^44,45^ Additionally, the conformational space of IDPs is immensely high-dimensional, and clustering performed directly on the MD trajectory is inefficient. Therefore, dimensional reduction (DR) techniques^45,46^ have to be applied before clustering. DR is used in machine learning and data analysis to reduce the number of variables or features in a data set.^47,48^ It has been pointed out that the complexity of IDP structural data requires the use of nonlinear techniques such as Uniform Manifold Approximation and Projection (UMAP)^49^ and t-Distributed Stochastic Neighbor Embedding (tSNE).^50^ A recent study that combines the latter with K-means clustering^45^ has shown that tSNE is very effective in separating heterogeneous IDP conformations into homogeneous subgroups. The homogeneity of the clusters obtained was assessed in terms of global structural metrics such as RMSD or end-to-end distance. The work provided an unprecedented insight into the latent spaces of IDPs derived from tSNE and their dependence on the hyperparameter setup employed. Two critical aspects of clustering by tSNE were beyond the scope of the work and were not covered by the authors. (1) No quantitative validation of the resulting ensembles against the experimental data has been performed. Consequently, (2) there was no need to determine the cluster centers that could be used as representative structures for validation.

The principal objective of the present study is to derive sufficiently small conformational ensembles of IDPs for reliable, cost-effective NMR calculations and predictions based on MD simulations. In pursuit of this goal, we integrate DR techniques and CAs to design a comprehensive protocol that yields IDP ensembles consistent with experimental NMR data. The development of the protocol builds on (1) the assessment of different tools for the prediction of CSs in IDPs, (2) the evaluation of various DR techniques, both linear and nonlinear, and (3) the analysis of the clustering quality based on internal statistical indicators as well as based on validation against experimental NMR CSs.

To ensure an accurate depiction of the conformational dynamics of the IDPs we are examining, we evaluate the efficacy of different fingerprints for DR, including ϕ/ψ angles, α-carbon distances and angles, and solvent-accessible surface area (SASA), to capture phenomena critical to CS variations. We benchmark the hyperparameters of tSNE to assess their impact on the DR and the correlation of the predicted CSs with the experiment. For this purpose, we also propose a metric enabling the identification of the cluster centers, i.e., structures that represent the conformational characteristics of the clusters in question. The CEs derived from the cluster centers are then rigorously compared with traditional sampling methods to validate their efficacy.

## Methodology

**Figure 2:**
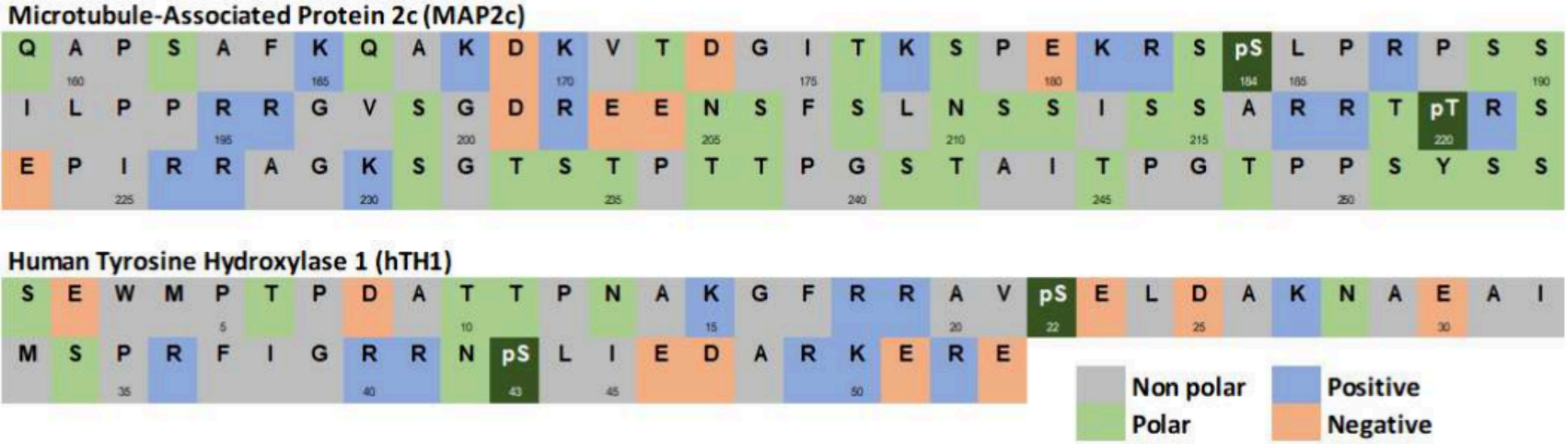
MAP2C and hTH1 single letter amino acid (AA) sequence codes with the phosphorylation sites identified.

Four proteins (hTH1, MAP2C, GB3, and UBIQ) were simulated for this investigation. MAP2C is a microtubule-associated protein involved in stabilizing the cytoskeleton of neural cells. The N-terminal region (<40% of sequence) is disordered but contains a segment with <80% propensity to form an α-helix.^51^ Phosphorylation in this region seems heavily dependent on Ser^184^ and Thr^220^.^52^ hTH1, human tyrosine hydroxylase I, is involved in the biosynthesis of dopamine^53^ and dysfunction has been linked to neurological diseases, such as Parkinson’s.^54^ 65 residues of the regulatory domain were determined biologically relevant,^55^ and the first 53 residues were simulated (Figure 2) for 2 μs, phosphorylated at Ser^22^ and Ser^43^. Phosphorylation at Ser^43^ showed a nominal influence, while phosphorylation at Ser^22^ encouraged phosphorylation at Ser^43^, which affects the activity of the protein.^56^ Ubiquitin is a small regulatory protein found in most tissues of eukaryotic organisms. It plays a key role in various biological processes, including marking proteins for degradation, altering their cellular location, and affecting their activity.^57^ Its structure has been characterized (1ubq) by NMR spectroscopy.^58^ GB3 is a cell surface protein found in Streptococcus bacteria. It binds to IgG, a type of antibody with high affinity.^59^ The structure of GB3 (2oed) has been determined by X-ray crystallography, both alone and in complex with an antibody fragment.^60^

### MD Simulations

All molecular dynamics (MD) simulations were performed using GROMACS,^61^ and the proteins were solvated in an aqueous environment with ionic species Na^+^ and Cl^–^ at 100 mM. The simulations ran at 298 K, 1 atm pressure (STP) using a 1 fs time step and the Velocity Verlet algorithm, a numerical integration algorithm using the Newtonian equations of motion. Neighbor searching was performed every ten steps. The Particle Mesh Ewald (PME) algorithm was used for electrostatic interactions with a one nm cutoff. A reciprocal grid of 120 × 120 × 120 cells was used with fourth-order B-spline interpolation. A single cutoff of 1.004 nm was used for Van-der-Waals interactions. Temperature coupling was done using the Nose-Hoover algorithm, while pressure coupling was done using the Parrinello–Rahman algorithm. The AMBER99SB-ILDN force field was used, as suggested by a previous investigation^51,62−65^ and the four-point TIP4P-D water model was implemented due to its success in maintaining disorder in an IDP trajectory.^51,66^ The trajectory lengths varied depending on the protein and whether the protein was ordered or disordered in nature, and details are provided in Table 1. The starting structures for MAP2C was generated using Flexible-Meccano,^67^ and ASTEROIDS software^68^ with prior NMR chemical shift data. The starting structure in hTH1 was selected randomly from an existent 1 μs trajectory, in which the conformation was fully equilibriated. Three replicates were generated for the phosphorylated and nonphosphorylated MAP2C trajectories, each with one using a starting structure containing an α-helix observed experimentally using CD (MAP2C/C and MAP2C/H).

**Table 1:** Overview of Classical MD Trajectories Obtained for the IDPs and Ordered Proteins (OPs) of Interest and the Naming Schemes Used Throughout the Manuscript^a^

**Protein/xx**	**Residues**	**Type**	**Phosphorylated**	**Length**
MAP2C/A^51^	96	IDP	yes	1100 ns
MAP2C/B^51^	96	IDP	yes	500 ns
MAP2C/C^51^	96	IDP	yes	500 ns
MAP2C/H^51^	96	IDP	no	500 ns
MAP2C/NH1^51^	96	IDP	no	500 ns
MAP2C/NH2^51^	96	IDP	no	500 ns
hTH1^31^	53	IDP	yes	2000 ns
GB3*	56	OP	no	200 ns
UBIQ*	87	OP	no	40 ns

a Trajectories that were generated just for this publication are marked with an asterisk (*).

### Chemical Shift Prediction Tools

CS predictions were made for both ensemble types by various prediction software. Sparta+^33,69^ is a database searching program that utilizes protein sequences and structural homology to predict chemical shifts of known conformations. Sparta+ calculates the ϕ, ψ, and χ_1_ angles from PDB coordinate files, then matches them to the complete database, returning the nearest 20 matches. The program then implements a similarity score, considering minor differences to generate a weighting factor for each of the six nuclei observed (H_N_, H_α_, C_α_, C_β_, C′, and N). ShiftX2^34^ follows a similar procedure with different algorithms to derive CSs, showing marginally improved accuracy over Sparta+ in some systems.^70^ Prosecco^71^ utilizes a purely neural-network-derived algorithm trained entirely on amino acid sequences and NMR CSs for IDPs to predict the CSs.

**Figure 3:**
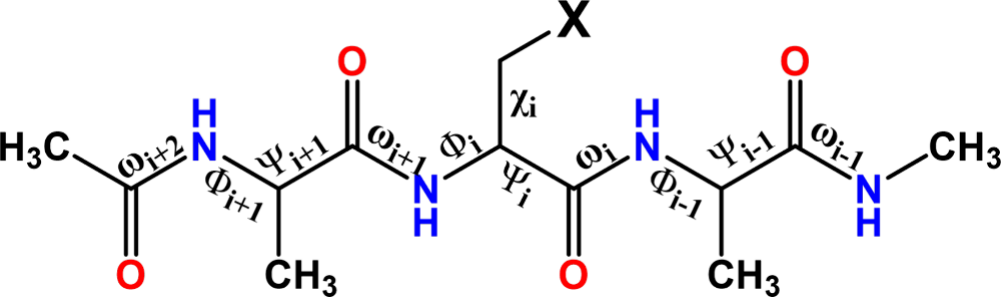
Example of the H_3_C–CO-Ala-X-Ala-NH–CH_3_ tripeptide used to compute the backbone contributions to the chemical shielding values. X represents the possible phosphorylated residue (Serine, Threonine, Tyrosine).

The ProCS-15 tool^35^ is a database–based method that predicts chemical shieldings using values from DFT calculations.^72^ It operates on models with the same sequence and structural pattern by calculating the chemical shielding based on a sum of three factors. First, chemical shielding is determined by a “naked” H_3_C–CO-Ala-X-Ala-NH–CH_3_ tripeptide (where X represents an arbitrary amino acid, see Figure 3) with a defined conformation. Second, the effects of side chains are taken into account. Third, the contributions of hydrogen bonding and the ring-current impact are considered. Since the ProCS-15 method does not use machine learning and instead relies on calculated chemical shielding values, there is a reduced risk of overfitting compared to other methods.

Since the original database of chemical shielding used by ProCS-15 does not contain chemical shielding for phosphorylated residues (pSer, pThr, pTyr), this chemical shielding had to be calculated and added to the database. The original FragBuilder code^73^ was extended to construct phosphorylated residues, and the modified FragBuilder tool was applied to generate input files to optimize the geometry of various tripeptide conformations. Geometry optimizations were performed in implicit water at the PM6^74^ level of theory using the Simple Virtual Screening technique. NMR calculations followed geometry optimizations in implicit water. The calculations used the OPBE functional^75−78^ and 6-31G(d,p) basis set.^79−83^ The computed chemical shielding values were compiled into a database along with the corresponding torsion angles. Necessary changes to the source code were introduced into the Phaistos and ProCS-15 source codes, allowing ProCS-15 to identify and process phosphorylated amino acids correctly. Common three-letter codes already defined in phaistos(ref (84))/ProCS-15 were employed: pSer – SEP, pThr — TPO, pTyr – PTR. The phosphate group was approximated as a carboxylate, allowing the use of an already available database of shielding corrections. The source code is available on GitHub.^85^ The complete procedure and methodology were described in the paper by Larsen et al.^35^

The built-in GROMACS, *dssp* tool was utilized to determine the secondary structure. Much of the trajectory analysis, radius of gyration (R_G_), end-to-end distances (EE_DIST_), solvent-accessible surface area (SASA), root-squared-mean deviation (RMSD), and root-mean-squared fluctuations (RMSF), were done through the *mdtraj*(86) python package, statistical analysis (e.g., kurtosis/skew) using *stats* package, and storage and management of large files utilized the *pickle*. Finally, to visualize and present the results, we used *seaborn* and *matplotlib* libraries.

**Figure 4:**
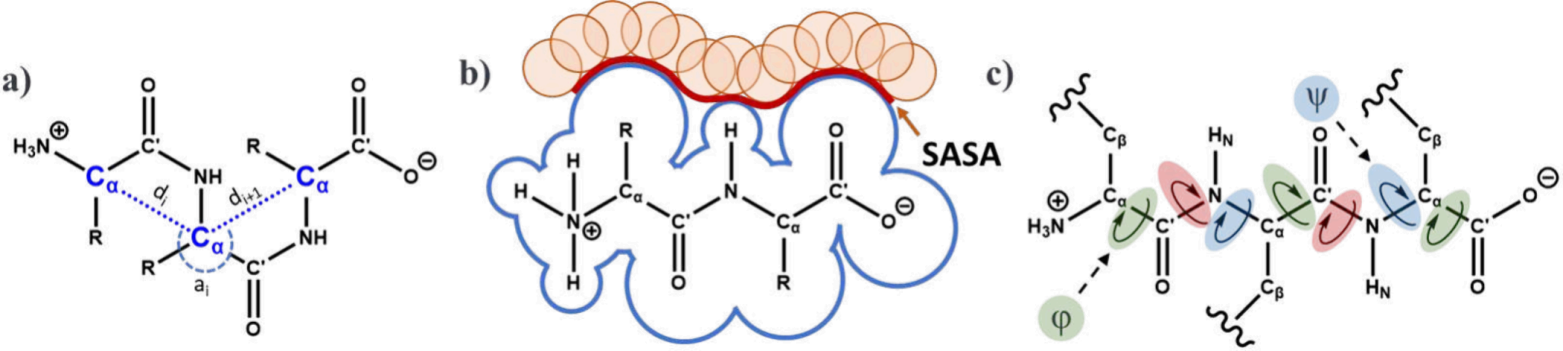
Features generated from the trajectory using (a) α-carbon distances and angles, (b) atomistic solvent-accessible surface area (SASA), and (c) ϕ and ψ dihedral angles.

Although CS prediction tools can be done on massive ensembles (>1000) or even the complete set, more in-depth computationally expensive tools can be made intractable when scaled to these levels. Additionally, these smaller ensembles provide insight into the states that can be divulged from the conformations, although oscillating between states that can interact with surrounding molecules or moieties. The traditional technique for generating these ensembles is sequential, with a median time step between each frame extracted from the trajectory (Figure 4). The Gromos clustering algorithm^87^ identifies clusters by selecting the structure with the most neighbors in a pool of structures and including its neighbors in the cluster, then repeating the process until all structures have been assigned to a cluster.^88^ Other clustering techniques implement specific features of the trajectory, such as α carbon distances and angles (Figure 4a), SASA (Figure 4b), the ϕ and ψ angles (Figure 4c) from the backbone. Pure clustering alone may not be sufficient, as it only examines similarities and differences between data points without considering the underlying structure or patterns in the data. In high-dimensional data, multicollinearity is a common issue with highly correlated features. Preprocessing the data while preserving the underlying patterns can help reduce noise and redundancy. Moreover, it allows for easier visualization and interpretation of the data in a lower-dimensional space, allowing for a qualitative assessment.

Linear dimensionality reduction was implemented by the *scikit-learn* package for principle component analysis (PCA) and linear discriminant analysis (LDA) methods, *deeptime* for the time-lagged independent component analysis algorithm (tICA). Nonlinear DR was done using *scikit-learn* for t-distributed stochastic neighbor embedding (tSNE). From *scikit-learn*, we also used k-means and agglomerative clustering algorithms and calculated the silhouette score to evaluate the quality of the clusters. In this paper, we explore the integration of DR and CA to obtain intelligently selected frames that still conserve the underlying data in the original trajectories.

## Results and Discussion

### Conformational Analysis

**Figure 5:**
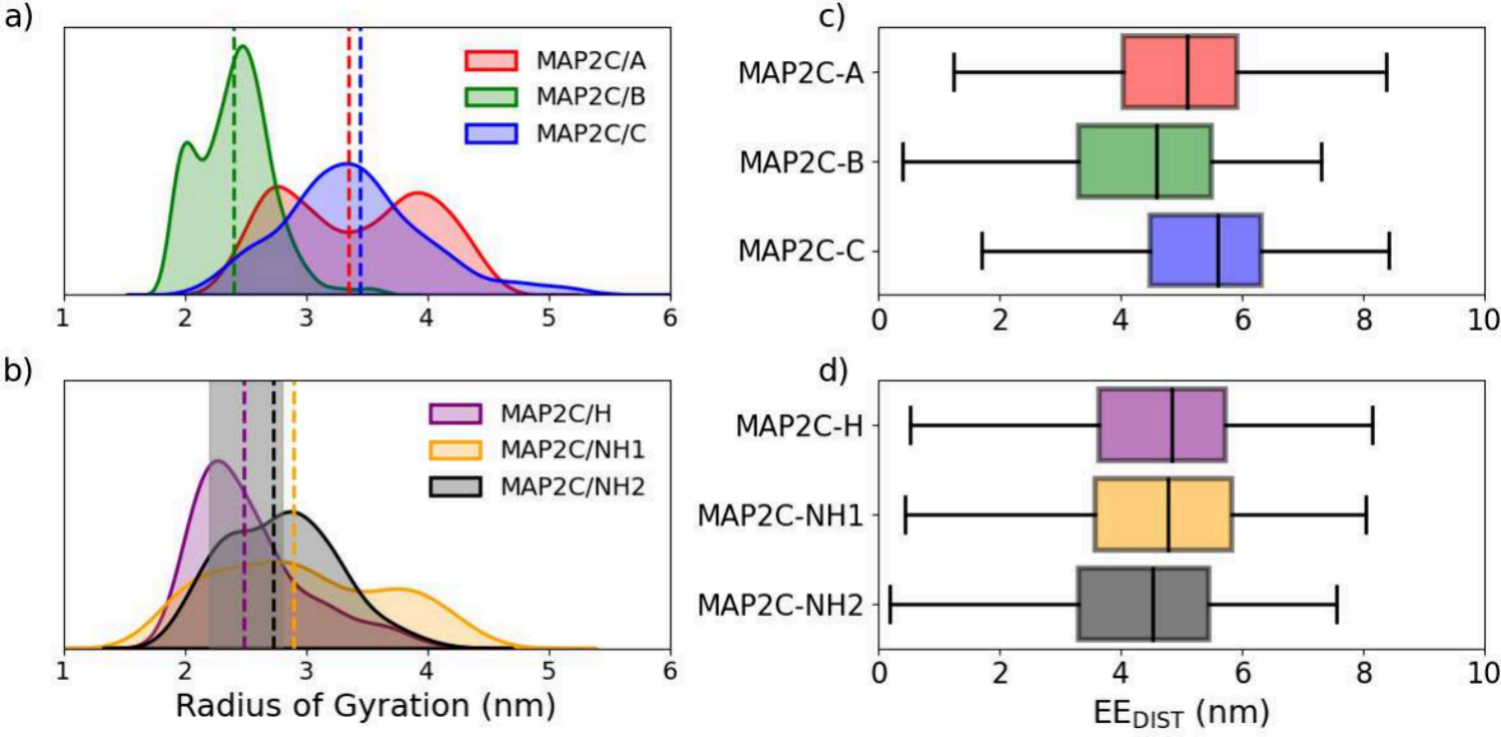
Computed R_G_ of each frame in the MAP2C trajectories are mapped according to their distribution with a kernel density estimation (KDE) for the phosphorylated (a) and nonphosphorylated (b) trajectories with the averages presented in dotted lines and the experimentally observed^51^ range shaded in gray for the nonphosphorylated. The end-to-end distance (EE_DIST_) is shown in box plots from the phosphorylated (c) and nonphosphorylated (d) trajectories.

All trajectories RMSD were computed and plotted in the SI (Figure S2) to ensure the trajectories are not trapped in microstates. Based on R_G_, the MAP2C/A and MAP2C/C trajectories produce similar averages (3.4 ± 0.6) and among the nonphosphorylated trajectories, MAP2C/H and MAP2C/NH1 have closer R_G_ (2.5 ± 0.5 and 2.8 ± 0.5 nm, respectively) to the experimentally obtained (2.5 ± 0.3 nm) as seen in the statistical analysis (Table 2). MAP2C/A and MAP2C/NH1 have negative kurtosis values from the R_G_, as the distribution is highly irregular. The distribution (Figure 5a/b) also shows that the MAP2C/A trajectory is bifurcated into expanded (≈4.0 nm) and contracted (≈2.8 nm) states during the trajectory. This is also reflected in their variances, as MAP2C/NH1 and MAP2C/A are distributed over a large range. MAP2C/B has a very low variance and is tightly clustered around the mean. For the hTH1 trajectory, the average R_G_ (1.9 ± 0.4 nm) indicates that the protein is also quite pluriform, and its kurtosis (1.63) and skew (1.27) suggest that the distribution of R_G_ values is skewed toward higher values, but is highly clustered around the mean (Figure S3). This is further supported by the high variance value (0.17), indicating a wide range of R_G_ values in the hTH1 trajectory. The EE_DIST_ plots (Figure 5c/d) show that the MAP2C/A and MAP2C/C are more extended on average than MAP2C/B, and both are more extended than each of the nonphosphorylated trajectories, with MAP2C/NH2 being the most compact.

**Table 2:** Computed Radius of Gyration (R_G_) Means (*x̅*), Standard Deviation (σ), Kurtosis, Skew, and Variance from Each Respective Trajectories

**Trajectory**	*x̅***(nm)**	**σ**	**Kurtosis**	**Skew**	**Variance**
MAP2C/H	2.54	0.46	0.84	1.16	0.21
MAP2C/NH1	2.95	0.70	–1.04	0.24	0.50
MAP2C/NH2	2.79	0.46	–0.43	0.27	0.21
MAP2C/A	3.41	0.61	–1.28	0.03	0.37
MAP2C/B	2.41	0.31	0.82	0.54	0.10
MAP2C/C	3.40	0.60	0.54	0.56	0.37
hTH1	1.91	0.41	1.63	1.27	0.17
GB3	1.09	0.01	0.51	0.63	0.00
UBIQ	1.18	0.01	0.31	0.43	0.00

**Figure 6:**
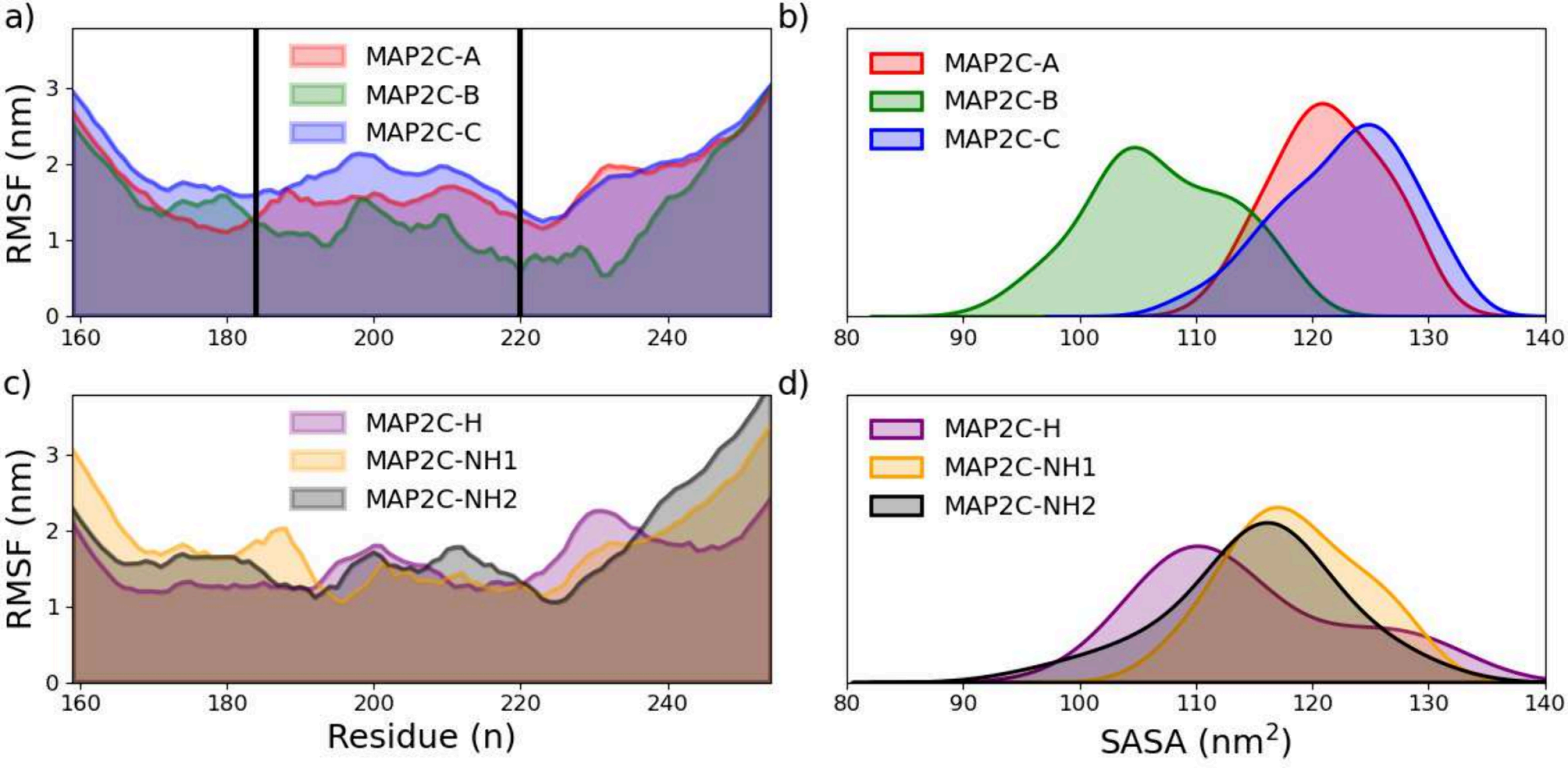
Root-mean-squared fluctuations (RMSF) calculated of the α-Carbon for each residue in the phosphorylated (a) and nonphosphorylated (c) trajectories, with black lines indicating phosphorylation sites, and the computed solvent-accessible surface area (SASA) for the phosphorylated (b) and nonphosphorylated (d) MAP2C trajectories.

Among the phosphorylated trajectories, MAP2C/A has the most significant fluctuation for most residues (Figure 6a), whereas MAP2C/B exhibits the least. Among the nonphosphorylated trajectories (Figure 6b), there is significantly less deviation between trajectories. Noticeably, the most significant fluctuation occurs at the end terminals of IDPs, a trend not observed in ordered proteins (Figure S23). It is vital to understand the differences and variations in these properties to cluster, as the primary focus of this investigation is on the generation of NMR chemical shifts. CSs depend on local interactions, either solvent-based or intramolecularly. To investigate the solvent interactions, the overall solvent accessibility was computed for each atom of the trajectory (Figure 6c/d) and showed a distinct divergence in interactions with surrounding solvents as well as intramolecular interactions.

The MAP2C/A and C trajectories have the most accessible surface area (≈122 nm^2^) and MAP2C/B the least (107 nm^2^), indicating that MAP2C/A and C exhibit more solvent interactions, while MAP2C/B simulates more intramolecular interactions. Among the nonphosphorylated trajectories, the SASAs are quite similar; MAP2C/H has the lowest SASA (114.4 nm^2^), although the variances (≈60) is nearly doubled in MAP2C/H and NH2 compared to their phosphorylated counterparts (≈30) as seen in Table S1 To compare between the proteins, the average SASA was computed per atom and shown in Table S2, demonstrating that disordered proteins have nearly double SASA per atom compared to ordered proteins. Additionally, from the ordered proteins, low variances in R_G_ and SASA reflect the reduced flexibility in GB3 and UBIQ, as it indicates a relatively tight distribution around the mean.

### Secondary Structure Analysis

Based on the DSSPs, approximately 56% of the MAP2C trajectories consisted of random coils, 62.6% in hTH1, 14.7% in GB3, and 20.4% in UBIQ (Table 3) as seen in the time-dependent DSSP (tDDSSP) plots (Figure S5–S8). Well-know stabilizing secondary structures, such as β-sheets and α-helices, appear in much higher quantities in OP versus IDPs. The MAP2C trajectories MAP2C/C and MAP2C/H have significant but transient appearances of α-helices, as seen in the tDDSSP plots. These structures were detected in the experiment and sought after in the simulation.^51^ Recent interest has emerged in the structural importance and contribution of polyproline type 2 (PPII) helices,^28,89,90^ as they have been observed in higher proportion in IDPs.^91,92^ Approximately 18% of the MAP2C trajectories had PPII helices, higher than hTH1 (8.9%), GB3 (0.0%), and UBIQ (2.2%).

**Table 3:** Secondary Structure Predictions (%) for Each of the Trajectories Using DSSP Implemented by GROMACS

**Trajectory**	**Coils**	**Bridges**^a^	**Helices**^b^	**PPII**	**Bend**	**Turn**	**Phos. Res.**
MAP2C/H	53.2	2.5	5.3	17.0	18.0	4.0	0.0
MAP2C/NH1	55.9	1.3	1.0	18.9	19.1	3.8	0.0
MAP2C/NH2	55.8	2.6	0.3	18.9	18.7	3.7	0.0
MAP2C/A	56.3	0.7	2.0	19.0	16.4	3.5	2.1
MAP2C/B	55.2	3.5	0.4	15.3	16.7	6.9	2.1
MAP2C/C	57.6	0.4	2.3	18.2	16.2	3.2	2.1
hTH1	62.6	1.8	1.5	8.9	16.4	5.0	3.8
GB3	14.7	42.3	26.5	0.0	7.1	9.3	0.0
UBIQ	20.4	33.9	19.8	2.2	4.6	19.1	0.0

a Combination of isolated and extended β-sheets.

b Combination of 3, 4, and 5-turn α-helices.

In hTH1, these PPII helices appear significant (>30%) in specific regions, Pro^5^–Pro^7^, and Ser^34^–Arg^36^, which may be stabilizing features of the protein (Figure S9). The PPII helices in the nonphosphorylated MAP2C trajectories seem to form indiscriminately throughout the chain, with solid favoritism around sites Ser^222^–Arg^226^, a structure which is maintained upon phosphorylation, with a significant drop in MAP2C/B only (Figure S9). MAP2C/B deviates from the other trajectories additionally with a notable decrease in α-helices structures, which are instead replaced with β-bridges and turns between Arg^227^ and Ser^231^. These structures may be significant or an artifact of the trajectory. Additional information about the global properties of the trajectories of interest can be found in Section S1 of the SI.

### NMR Chemical Shifts

In addition to global and secondary structure analysis, we seek to evaluate the molecular dynamics for local properties, such as interactions, hydrogen bonding, and NMR CSs. As discussed previously, to aid in calculating CSs, neural network-derived CSP (e.g., Sparta+, ShiftX2) and database-derived CSP (ProCS-15) can produce relatively accurate CSs from a trajectory significantly faster than DFT calculations. A comprehensive comparison of CSPs on sequential ensembles can be found in Section S2 of the SI, and the method of developing these SEs is discussed in Section S3 of the SI. Among the nonphosphorylated MAP2C trajectories, MAP2C/NH2 performs the best, followed by MAP2C/H and MAP2C/NH1 (Table S5). MAP2C/C shows the greatest agreement with experimental, and then MAP2C/A and MAP2C/B, in that order (Figures S17–S18). There were several strong outliers in each of the trajectories discussed and described in full in the SI. Still, the overall trend is that the best-performing atoms according to their R^2^ values are C_β_, C_α_, C′, N, H_α_, and H_N_, although the trend for the RMSE is H_α_, H_N_, C_β_, C′, C_α_, and N (Table S5 and S3, respectively).

**Figure 7:**
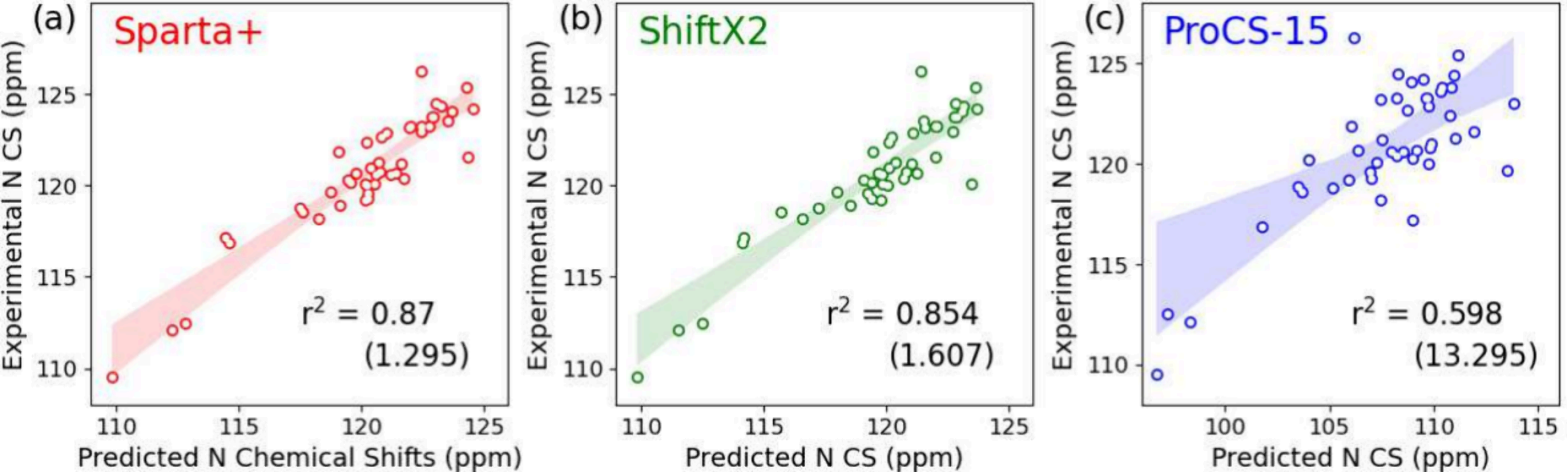
Correlation between experimental and predicted N chemical shifts in hTH1 using (a) Sparta+, (b) ShiftX2, and (c) ProCS-15 with r^2^ and root-mean squared error in parentheses.

**Figure 8:**
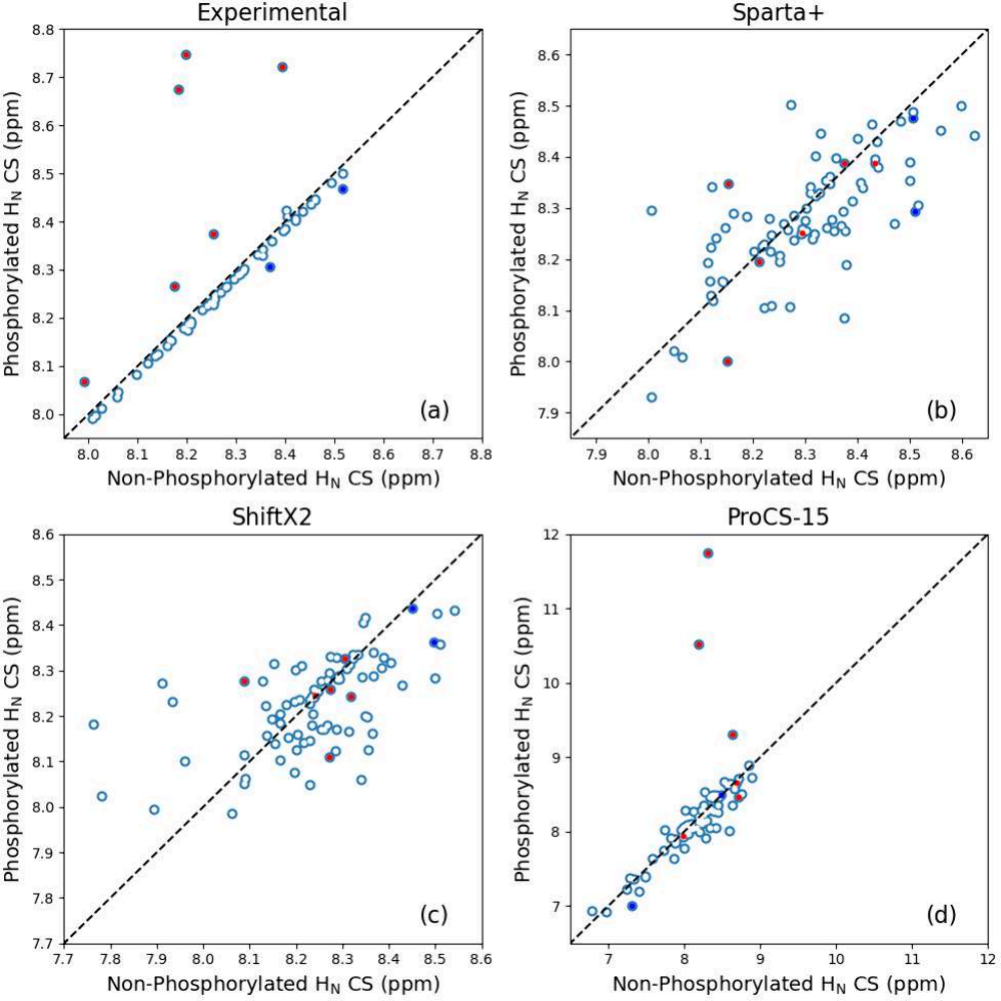
Influence of the phosphorylation of the protein on the ^1^H_N_ CS by residue plotted for experimental (a), Sparta+ (b), ShiftX2 (c), and ProCS-15 (d) with specific shifted residues marked red (upshifted) and blue (downshifted).

Performance rankings for CSPs differ between different atoms and proteins, but some trends can be observed. For the intrinsically disordered proteins (hTh1 and MAP2C), Sparta+ and ShiftX2 produced relatively good agreements for C_α_, C_β_, and N, although it was not great for H_α_ and H_N_. Sparta+ also performed better than ShiftX2 for calculating nonphosphorylated chemical shifts. ShiftX2 was better at predicting C′, and Prosecco best predicted CS based on amino acid sequences. Nevertheless, since Prosecco uses only amino acid sequences as input, it cannot be used to evaluate the performance of MD trajectories. Sparta+ and ShiftX2 ignore proline N atoms due to the challenges in assigning them experimentally, as they require customized heteronuclear NMR experiments.^93^ Prosecco performs quite well in predicting chemical shifts on the basis of protein sequences. However, its usage is limited because of its inability to describe dynamic behaviors, while Sparta+ and ShiftX2 are well suited for describing dynamic behavior, as they utilize conformation information in the form of PDB files. ProCS-15 performed poorly in predicting chemical shifts for ordered and disordered proteins, as highlighted in Figure 8. It was outperformed by the most commonly used prediction tools, such as Sparta+ and ShiftX2, in terms of both accuracy and linearity for the chemical shift data examined in this study. Furthermore, ProCS-15 showed a higher standard deviation for some residues than other prediction tools, suggesting inconsistent performance for different amino acids (Figure 7). Thus, while it is helpful in specific applications, the general performance of ProCS-15 indicates that it may not be a suitable prediction tool for all protein systems and conditions, and its predictions should be used with caution.

### Phosphorylation Influence

The CSs from the phosphorylated and nonphosphorylated proteins of MAP2C were compared, and the CSPs were evaluated for their ability to describe phosphorylation. The MAP2C trajectory was phosphorylated at the Ser^184^ and Thr^220^ sites, and the experimental chemical shifts were derived for comparison.^51^Figure 8 shows the influence of phosphorylation on the H_N_ CSs; the N plots produced similar trends (Figure S21) although the variation is less pronounced. In addition to the phosphorylation sites (Ser^184^ and Thr^220^), four residues were deshielded upon phosphorylation (Arg^221^, Thr^219^, Leu^185^, and Ala^164^) and two were shielded (Glu^223^ and Glu^180^). These six residues represent sites where the phosphorylation alters the chemical environment or the phosphate group interacts intramolecularly with the respective H_N_. Those that were shielded were both negatively charged residues near the phosphorylation sites. Residue Ser^183^ was not affected by phosphorylation despite its proximity, and the MD trajectory suggests an atomic-level explanation. Due to interactions between the phosphorylated Ser^184^ and Arg^187^, the protein appears pinched in many parts, which prevents the phosphate from interacting with its neighbor. The study revealed that recent neural network-based CSPs, such as Sparta+ and ShiftX2, could not incorporate the influence of phosphorylation, which was expected due to their reliance on data sets without phosphorylated CSs. However, the DFT-trained CSP, ProCS-15, distinguished three relevant phosphorylation shifts, indicating potential for this approach, although signficant improvement must be made toward improving its accuracy and precision. This highlights the importance of developing more advanced prediction methods to accurately account for the influence of phosphorylation, which would require smaller ensemble sizes. Furthermore, the limitations of Sparta+ and ShiftX2 highlight the need for higher-level, precise prediction techniques.

### GROMOS-Clustered Ensembles

Before we embarked on the derivation of various clustered ensembles, we constructed SEs for comparison, see Section S3. CEs can be generated from different characteristics of the trajectories. To develop a CE, we must first decide on the logical feature based on which clustering will be performed. The Gromos algorithm^87^ built into GROMACS^45,94^ employs RMSD, and we implemented the algorithm in this investigation on several trajectories at different RMSD cutoff points. The resultant CEs were averaged and compared to sequential ensembles of the same size (Figure S24–S25), and additional figures and explanations can be found in Section S4 in the SI. Regardless of the ensemble size or trajectory, the CEs produced less agreement with experimental values in all trajectories and atoms tested. Even for the less pluriform, more homogeneous GB3 protein trajectory, the ensembles produced rarely exceeded agreement with those generated sequentially. One reason is that the RMSD matrix used for this method captures the system’s overall behavior rather than focusing on specific regions or interactions. This can be problematic as it ignores local fluctuations and conformational changes, which are crucial for accurately describing the behavior of macromolecules reflected in NMR CSs. As a result, CEs generated using this technique fail to capture the level of detail necessary for further analysis. Better considerations of the possible clustering features of the protein, both local and global, are required to generate more precise CEs, particularly for disordered systems.

### Dimensionality Reduction

Multicollinearity (high correlation between features) in high-dimensional data sets can confound clustering results. Multicollinearity arises when two or more predictor variables are highly correlated. This can lead to difficulties in various analytical tasks, including clustering.^95^ To address this, dimensionality reduction techniques can be applied. These methods reduce the number of features (dimensions) while preserving essential information. Doing so mitigates noise, redundancy, and multicollinearity, leading to more effective clustering. By reducing the dimensions, we also create a more manageable representation of the data, making it easier to visualize and analyze. Each dimensionality reduction yields distinct latent spaces (Figure S26). These spaces emerge from intricate variations in input features, unveiling diverse patterns within the system and singling out specific states that may be of significance biologically. Choosing a suitable DR technique can depend on many factors, outlined in Section S5. For our purposes, the linear method tICA and nonlinear method tSNE were selected to generate the reduced landscapes in the procedure. Several other linear (PCA, LDA) and nonlinear (UMAP) techniques were attempted, although, as explained in Section S5, many were discarded for various reasons.

**Figure 9:**
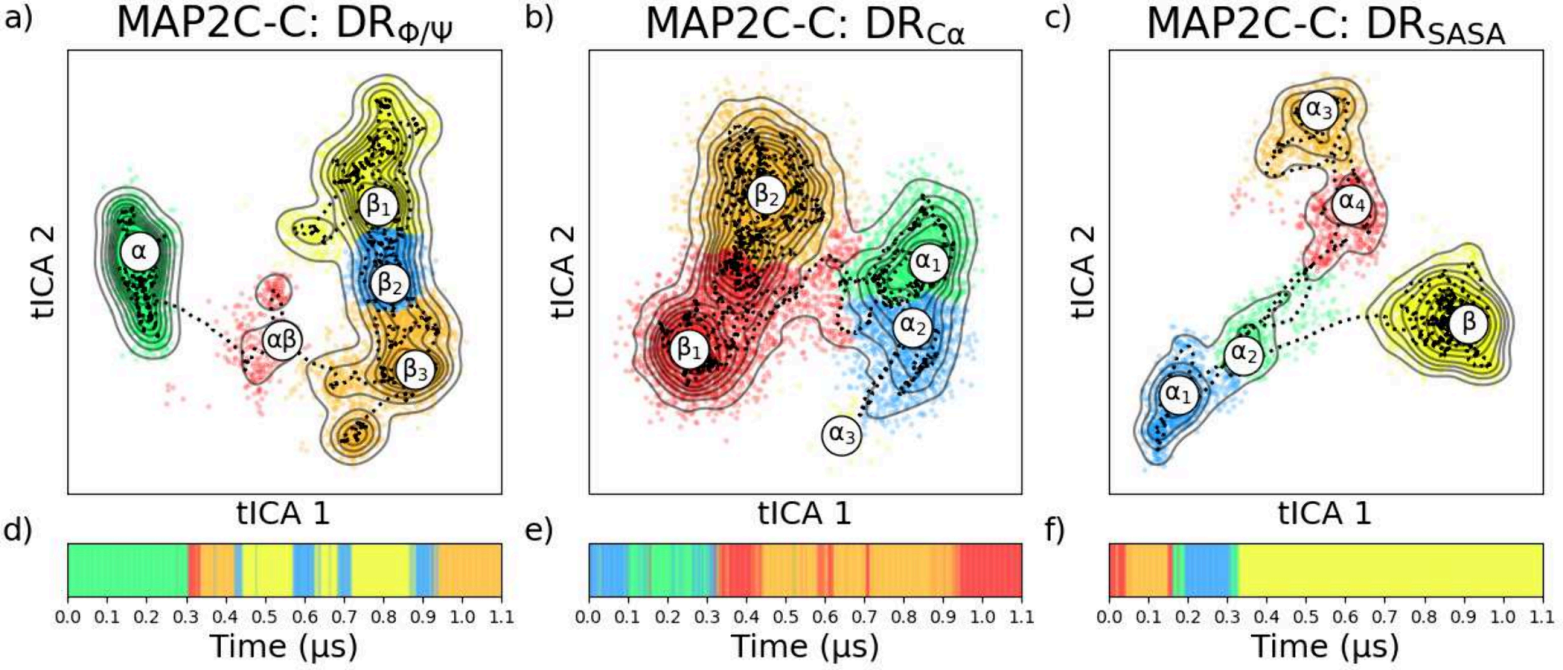
Dimensionally reduced landscapes of MAP2C/C clustered into five labeled clusters (a-c), and the respect place within the trajectory (d-f) using different input features; ϕ/ψ (a), α carbons distances and angles (b), and SASA (c).

**Figure 10:**
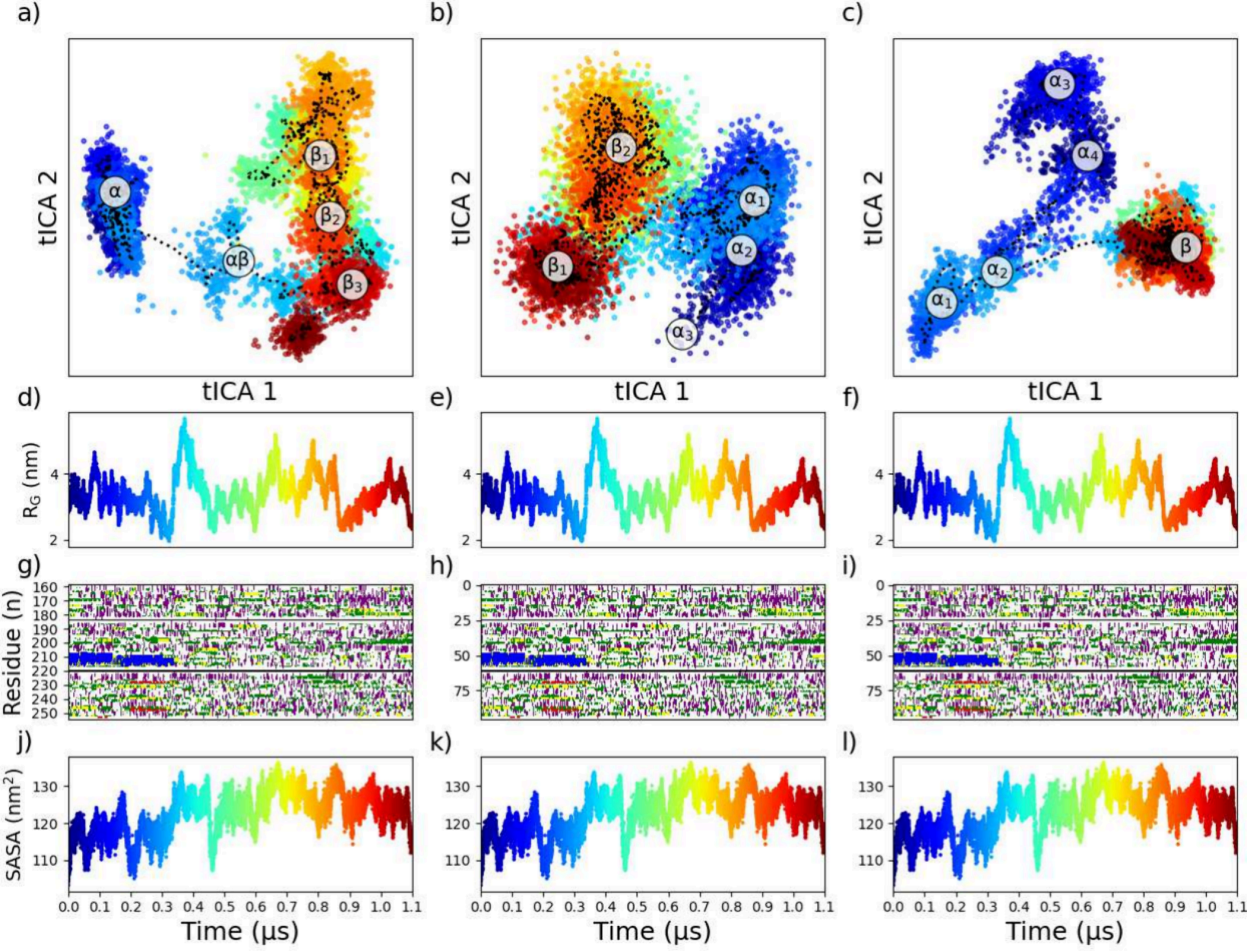
Distribution of conformations from the MAP2C/C trajectory on different DR latent spaces; ϕ/ψ (a), α carbons distances and angles (b), and SASA (c). Rolling averages are included to show the overall path of the trajectories (a-c), and several collective variables are plotted, such as radius of gyration (d-f) and total SASA (j-l) as a function of time. The DSSP plots of the secondary structures (g-i) are included for comparison.

In order to implement DR, specific input features must be selected to adequately describe the system. tICA was implemented using each of the characteristics (ϕ/ψ angles, α-carbon distances and angles, or SASA) and the hierarchical agglomeration (5 groups) in the resultant manifold (Figure 9) shows some significant variations between representative spaces. The choice of agglomerative hierarchical clustering over faster but less accurate techniques like K-means or highly parametrized techniques like DBSCAN likely stems from its ability to more accurately capture the underlying structure of the data without being heavily influenced by parameter settings. The hierarchical nature of this method offers a nuanced understanding of data grouping at different levels of granularity, which is crucial to accurately capture the complex biological phenomena represented in the MAP2C/C trajectory and the various ways in which it can be described (Figure 10). Approximately 40% of the MAP2C/C trajectory showed the existence of α-helices, which is clearly observed in the DSSP plot (Figure 10g-i and SI: Figure S6c) of the protein. Assuming that the DR is accurate, this trend and others should also be accurately captured in the latent spaces.

In the ϕ/ψ DR (DR_ϕ/ψ_), the backbone conformations are the main contributors to the expression of the conformational landscape. Thus, the state DR_ϕ/ψ_^α^ exhibits a high propensity (approximately 7.1 ± 1.9 residues) for α-helices (Table S9), three states (DR_ϕ/ψ_^β_1_^, DR_ϕ/ψ_^β_2_^, DR_ϕ/ψ_^β_3_^) lack instances of α-helices, and a transitional state (DR_ϕ/ψ_^*αβ*^) that spans a continuous range between these extremes, with partial α-helices characteristics (≈5 residues). In the case of the SASA DR (DR_SASA_), fingerprints strongly favor solvent intractability instead of only backbone conformations. Because of this, conformations with α-helices are separated into four states (DR_SASA_^α_1_^, DR_SASA_^α_2_^, DR_SASA_^α_3_^, DR_SASA_^α_4_^), and one state (DR_SASA_^β^) with no α-helices. As seen in Figure 9a-c, many of these clusters overlap, as the transition between these states is much more continuous, and there is no significant barrier between the sampled conformations. This can also be seen by the rolling average “path” of the trajectory, shown in the dotted line (Figure 9a-c), which demonstrates that the protein travels within the α and β states with minimal restriction, but is hindered from traversing between them, either indicating poor sampling in the trajectory data or rare phenomena with a high energy barrier. To achieve a robust ensemble from DR/CA, there needs to be some assurance that the centers of each cluster in an ensemble give a good representation of the full trajectory. Similar plots and DR of the other MAP2C trajectories can be found in the Supporting Information (Figures S31–S35). Based on silhouette scores and Davies-Bouldin index (Table S8), SASA produced the best clustering separation of all features, although an investigation of the cluster sizes is warranted. It is important to note that the success of DR_SASA_ with IDP may not produce satisfactory results when applied to ordered proteins, as seen in Figure S38d-i, where GB3 and UBIQ exhibit very different behavior with a preference for DR_ϕ/ψ_.

Several methods were explored to select a representative center from each cluster identified by cluster analysis. An initial approach involved identifying the Euclidean center and choosing the closest conformation on the reduced landscape. However, this method proved to be insufficient due to technical limitations and the potential for outliers to be selected as representatives. As an alternative, the Euclidean center was computed for each cluster on the higher-dimensional landscape, and the structure closest to this point was selected as the center representative. However, this method also had drawbacks and did not yield satisfactory results. To address these limitations, a new approach was used based on the concept of density in the reduced landscape. Specifically, the Gaussian density of the reduced landscape was calculated, and structures residing in the highest-density regions of the latent space were chosen as representative centers for their respective clusters. This method minimized the risk of selecting outliers as representatives, ensuring that the chosen structures accurately represented the characteristics of their clusters.

**Figure 11:**
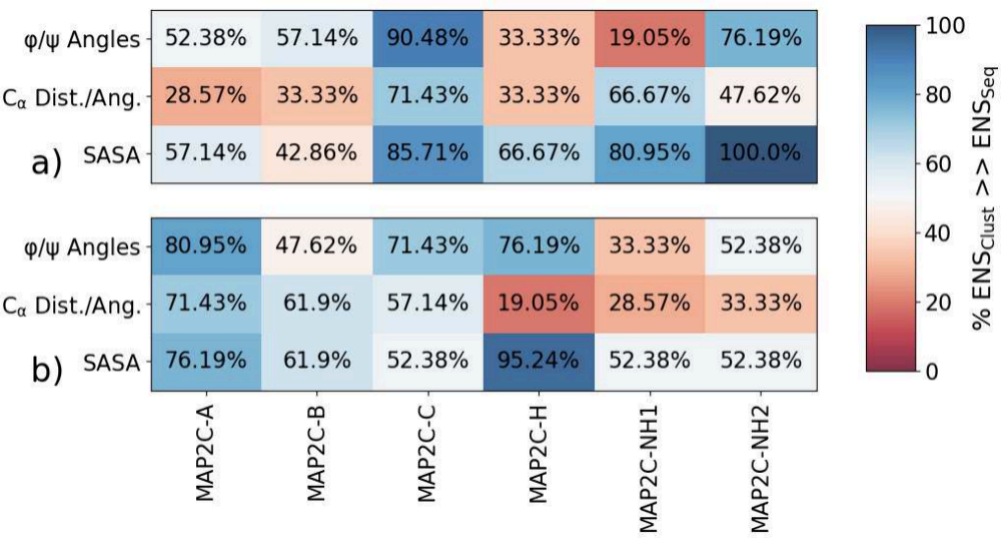
Performances of the clustered ensembles, represented as the % of ensembles which outperform sequential ensembles, using different input features and linear dimensionality reduction on different trajectories for H_N_ and N chemical shifts.

Using this method to generate CE and compare them with SEs of similar size, we can average the chemical shifts of each to determine which method produces the best ensemble consistently in relation to experimental chemical shift averages. Linear techniques seem to adequately group the trajectories into small ensembles (<10) as seen in Figure 11, although as recorded in Section S3, this ensemble size is insufficient to describe the complex behavior of the protein for the purpose of NMR CS. The results of our study consistently demonstrate the superior performance of CEs over SEs. This is evident in most of the models produced, as shown in the histograms and scatter plots in Figures S48–S50. Although there are some exceptions, such as MAP2C/A ϕ/ψ DR N atoms, MAP2C/B α DR N atoms, and MAP2C/C SASA HN atoms, the trend toward improved performance in smaller ensembles is clear. As the ensemble size approaches 500, the difference between clustered ensembles and sequential ensembles becomes increasingly negligible. This is expected as the sequential ensembles reach convergence in this range, and the addition of new frames has little impact on overall performance. Nonlinear DR techniques are recommended for larger ensemble sizes (>50). Nonlinear DR techniques effectively generated larger ensembles of trajectories due to their ability to capture complex nonlinear relationships, account for higher-order interactions, handle nonuniform and high-dimensional data, and preserve the local and global structure of the data.

**Figure 12:**
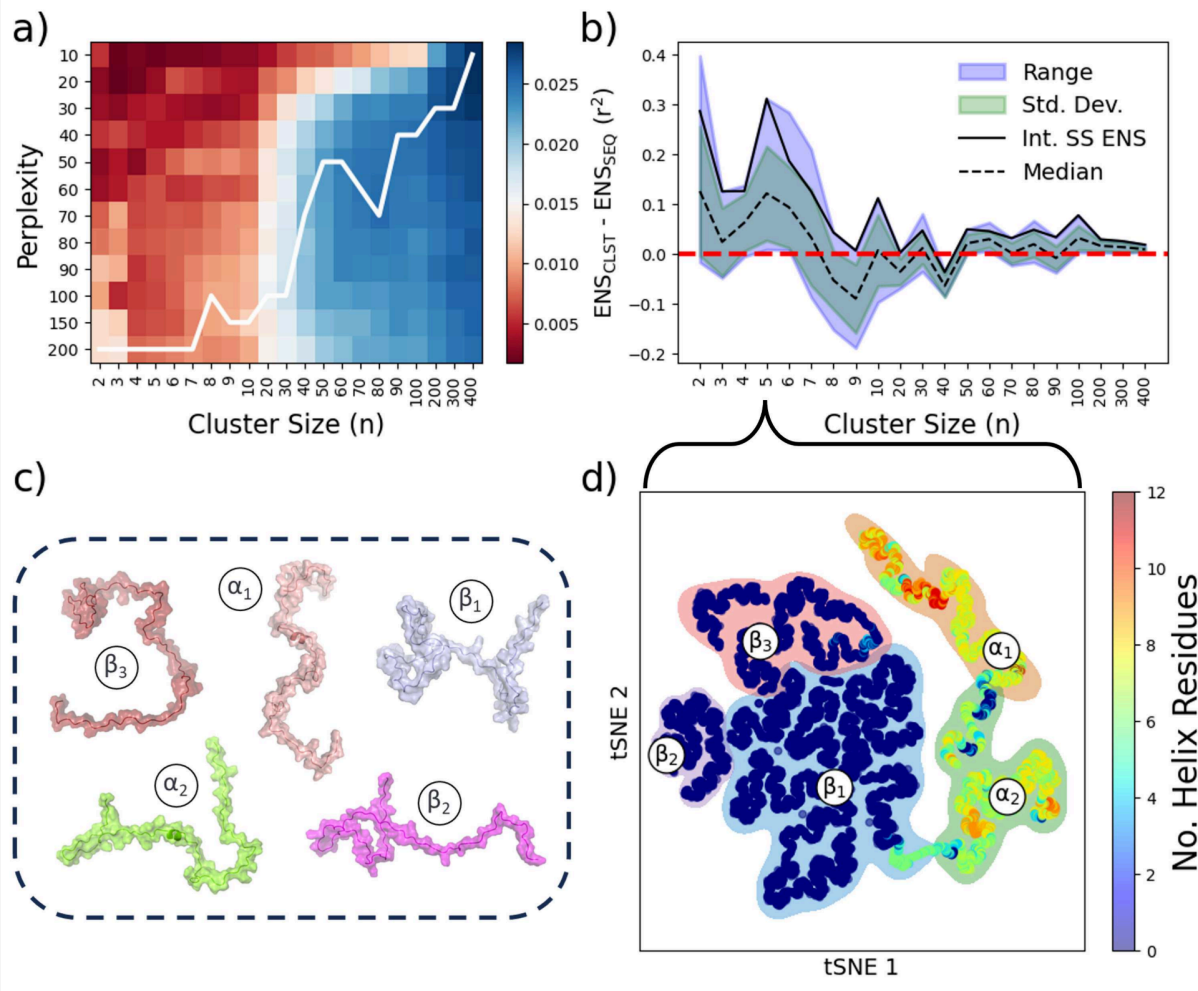
Representation of the best ensemble selected from (a) the integrated silhouette score, SS_INT_, (b) the relevant performances derived from CEs compared to SEs, and the performance of that selected by SS_INT_, (c) a representation of the DR latent space and subsequent clustering, and (d) visual representation of a small cluster with excellent CS agreement.

To compensate for the additional parametrization required for a nonlinear technique such as tSNE, we devised a protocol to select the best DR based on a metric, integrated silhouette score, SS_INT_.^45^ The SS_INT_ combines the clustering quality from both the higher and lower dimensional space, as described in Section S7 in the SI. For each feature tested, the perplexities were scanned as seen in Figures S39 - S44, and the best SS_INT_ DR was selected for clustering at each ensemble size (Figure 12a). The resultant set was compared to ensembles of similar sizes generated sequentially and performed well (Figure 12b). Not all cluster sizes produce better ensembles than traditional sequential ensembles; however, utilizing SS_INT_, we were able to achieve ensembles with better performance than the averaged CEs derived, and the selected ensembles perform better than SE in ≈85.7% of cluster sizes. The best-selected ensemble (n = 5) is shown in the latent space (Figure 12d) with data point shading representing the number of α-helices, and representative structures from the ensembles can be seen in Figure 12c. It should be noted that the ensembles generated by this methodology are tested primarily for NMR chemical shifts, and further experimental data would better improve the technique and avoid the risk of overfitting.

**Figure 13:**
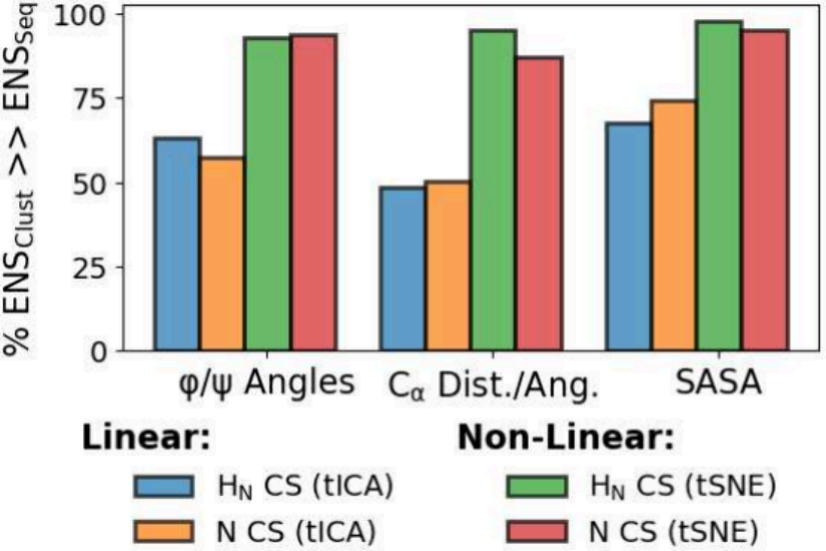
Comparisons of the different dimensionality reductions and clustering, % of ensembles generated through clustering (ENS_Clust_) that outperform those generated from sequential (ENS_Seq_).

Similar clustering can be implemented on each of the other phosphorylated trajectories to avoid overfitting of the model (Figure S51–S53), and each time implemented the number of ensembles which performed better than sequential increased, by an average of ≈38%. The amount that the techniques improved the sampling in the subsequent ensembles varied depending on the sampling quality of the original trajectory. Even still, all features and trajectories generate CEs that, on average, performed better than the sequential ensembles, suggesting a robust technique for generating CEs. A compilation of all MAP2C trajectories and the percent of CEs that outperform similar-sized SEs can be seen in Figure 13, highlighting the benefits of both utilizing SASA as a feature for dimensionality reduction and the use of scanning the SS_INT_ to determine the ideal matching of perplexity and cluster size. These ensembles not only match local properties such as chemical shifts, but also exhibit a similar global structure compared to the original trajectories, as indicated by the agreement of properties such as R_G_ and RMSD (Figure S54). This provides further evidence that our method effectively captures the true conformational space of the protein and allows for the identification of representative conformations that accurately reflect the dynamics of the system.

## Conclusion

This study illustrates the effectiveness of integrating DR and CA techniques to generate CEs of IDPs that align well with the NMR CS predictions. Within the spectrum of rapid CSPs assessed, Sparta+ emerged as the preeminent choice, albeit with discernible performance disparities dependent on the atom type. The accuracy in predicting the CSs of nitrogen and carbon atoms was notably higher, with a r^2^ of no less than 0.9 for all trajectories, ordered and disordered. In particular, in the hydrogen atoms, there is a substantial drop in agreement, from 0.95 to 0.72 (H_α_) and from 0.84 to 0.49 (H_N_). This can be attributed to a lower range in ppm for H atoms, coupled with the importance of solvent interactions, which are more important to IDPs than OPs. The former suggests the importance of reporting the error in absolute terms (RMSE) as well as r^2^, and the latter is given credence by the abnormally large range of performance depending on the MAP2C replicate. Additionally, the methodologies could not effectually capture the influence of phosphorylation, except for ProCS-15, which is explainable as the neural networks were not trained on phosphorylated samples, while the database of ProCS-15 was. Consequently, these findings emphasize the need for advanced methodologies that can more accurately predict CSs within IDPs, particularly for incorporating phosphorylated residues or PTMs.

In the exploration of methodologies to generate CEs that would make such higher-level computation techniques tractable for CS predictions, we utilized GROMOS^87^ CA to generate CEs, highlighting its limitations for IDPs. With few exceptions in the OPs and small ensembles, these ensembles markedly under-performed in alignment with experimental data. The algorithm’s focus on RMSD as a clustering metric fails to account for the specificity’s of local interactions and conformational dynamics, which are essential for precise NMR CS modeling in IDPs, pointing toward the necessity for a more nuanced approach in selecting clustering criteria. DR served as a preliminary step to clustering to mitigate the computational and analytical challenges posed by the high-dimensional nature of IDP data, enabling a more focused and effective clustering process. Upon applying DR before clustering, several issues arose, such as the selection of input features, the decision to use linear or nonlinear methods, and the calibration of hyperparameters to refine the data analysis process.

This study explored the impact of using different fingerprints for DR on the analysis of IDPs, specifically focusing on the solvent-accessible surface area (SASA), ϕ/ψ dihedral angles, and α-carbon distances and angles. The distinct latent spaces generated from each feature set underscore the importance of feature selection in DR. SASA offers a more comprehensive view of the protein’s external interactions, including transient secondary structures and overall shape. In contrast, ϕ/ψ and α-carbon-based DR focus more on the protein’s backbone conformation and local structure, respectively, which excels particularly for capturing conformational changes in ordered proteins. The study further underscores the insubstantial impact of SASA associated with carbon and nitrogen atoms on capturing the system’s conformational dynamics. The study also indicates that no single feature set universally outperforms the others, suggesting that the choice of DR features should be tailored to the specific analytical goals and characteristics of the protein under study. This performance differentiation underscores the complexity of IDP analysis and the need for a nuanced approach to feature selection in DR techniques.

When choosing between linear and nonlinear DR techniques, the key considerations include the desired ensemble size and the data’s intricacy. For smaller ensembles (<10), linear DR methods such as PCA and TICA are adequate but may not fully capture the complex behaviors necessary for accurate NMR CS predictions. For larger ensembles (>50), nonlinear DR methods, such as tSNE, are preferred due to their ability to handle complex nonlinear relationships and high-dimensional data, effectively generating larger and more representative ensembles. Additionally, the utilization of the techniques outlined in this paper are highly dependent on the input source. Readers are therefore advised that replication might entail the use of verification of trajectories with experimental evidence or enhanced sampling techniques.

The integration of dimensionality reduction (DR) techniques prior to clustering significantly enhanced the clustering process for intrinsically disordered proteins (IDPs), as demonstrated by the creation of conformational ensembles that more precisely mirror the dynamic behaviors of these proteins. This was evidenced by the improved performance of these ensembles over those created through traditional sequential ensemble generation, with ensembles generated using DR and clustering techniques outperforming sequentially generated ensembles in approximately 85.7% of cluster sizes evaluated. This approach not only underscores the effectiveness of DR techniques in refining the clustering process but also highlights the potential of these methods to produce more representative and accurate conformational ensembles of IDPs. While the preliminary results of this investigation are promising, it should be noted that the current method was only trained on one form of experimental data and would benefit from the inclusion of additional sources to avoid overfitting, which is a future endeavor we plan to investigate utilizing more adept neural network approaches.

## Data Availability

All molecular dynamics simulation trajectories are available upon request. All input files to run the simulations, as well as our current implementation of the model, can be downloaded from the GitHub repository: https://github.com/Chemistry-Mike/NMR-Clustering-IDP. Additionally, all materials used to generate and run the ProCS from the source can be found available here: https://github.com/martinj80/phaistos.

## Supporting Information Available

(1) Additional trajectory analysis, including RMSD calculated for all trajectories in the nonphosphorylated and phosphorylated MAP2C, hTH1, and ordered trajectories (GB3/UBIQ), with the calculated root mean squared deviation to confirm that the trajectory was not trapped in any local minima or microstates. The variation in RMSD and the combined gyration radius (R_G_) kernel density estimation is discussed. (2) Analysis and investigation of protein trajectories, DR methods, and further information on the integrity and techniques described in the manuscript. (3) Information on the influence of protein phosphorylation on the chemical shift of ^15^N by residue, including experimental data and predictions from Sparta+, ShiftX2, and ProCS-15, with specific labeled shifted residues. (4) Performance comparisons of the ensembles generated by the GROMOS algorithm^87^ and sequentially for different types of atoms in MAP2C/A, MAP2C/C, and all types of atoms in hTH1. (5) DR techniques selected for the study, including principle component analysis (PCA), linear discriminant analysis (LDA), and time-lagged independent component analysis (tICA), with reasons for the selection and implementation details. (6) Details on the challenges in assigning proline 1H N and 15 N atoms in NMR experiments and the performance of various prediction tools, such as Prosecco, Sparta+/ShiftX2, and ProCS-15, for these atoms. (7) Discussion on the importance of selecting the appropriate input fingerprint for DR analysis in molecular dynamics simulations, including dihedral ϕ and ψ angles, α carbon distances and angles, and solvent-accessible surface area. (8) Comparison of different CEs achieved from utilizing different features for the fingerprints of the DR and clustering, with their averaged R_G_, RMSD, SASA, and EE DIST. (9) Regression plots and performance evaluations for various NMR chemical shift prediction tools, highlighting their deviations from experimental values and the impact of different factors like phosphorylation and protein order (PDF)
