# Supplementary material for: Streamlining NMR Chemical Shift Predictions for Intrinsically Disordered Proteins: Design of Ensembles with Dimensionality Reduction and Clustering
Source: J Chem Inf Model. 2024 Aug 5;64(16):6542–56. doi: 10.1021/acs.jcim.4c00809 (PMC11412307; doi:10.1021/acs.jcim.4c00809)
Supplement: Supplementary file 1 — ci4c00809_si_001.pdf [file ci4c00809_si_001.pdf]

Supporting Information for Streamlining NMR Chemical Shift Predictions for Intrinsically Disordered Proteins: Design of Ensembles with Dimensionality Reduction and Clustering

Michael J. Bakker,† Amina Gaffour,† Martin Juhás,† Vojtěch Zapletal,† Jakub Stošek,†,‡ Lars A. Bratholm,¶ and Jana Pavlíková Přecechtělová*,†

†*Faculty of Pharmacy in Hradec Králové, Charles University, Akademika Heyrovského 1203/8, 500 05 Hradec Králové, Czech Republic*

‡*Department of Chemistry, Faculty of Science, Masaryk University, Kotlářská, 611 37 Brno, Czech Republic*

¶*School of Chemistry, University of Bristol, Cantock's Close, BS8 1TS Bristol, UK*

E-mail: precechj@faf.cuni.cz

Phone: +420 495 067 488

This document contains much of the analysis and investigation into the protein trajectories, dimensionality reduction methods, and further information about the integrity and techniques described in the manuscript.

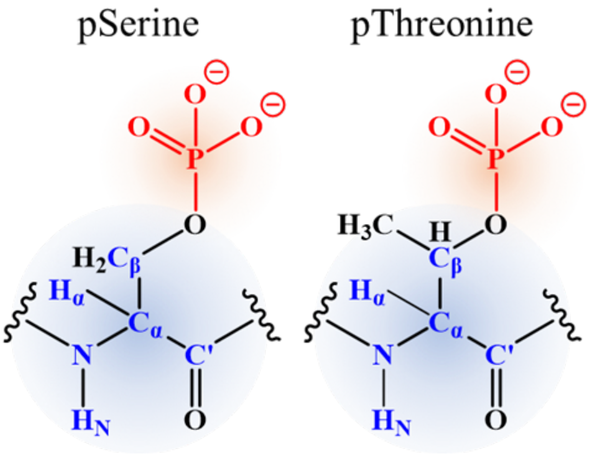

Figure S1: Example phosphorylation for **serine** (left) and **threonine** (right), the phosphate group highlighted in red. The individual atoms which will be discussed in this manuscript are highlighted in blue with their respective naming.

S1 Additional Trajectory Analysis

The root-mean-squared deviation was computed (Figure S2) to confirm that the trajectory was not trapped in any local minima or microstates. Noticeably, the RMSD variation in the ordered proteins was substantially lower than that in the disordered proteins. This trend can also be observed in the combined gyration radius (R_G) kernel density estimation plots (KDE), as seen in Figure S3. Additionally, this distinction between the ordered and disordered trajectories can be seen from the comparisons between both the total solvent-accessible surface area (SASA) and average SASA by atom (Figure S4), or by the statistical analysis of the total SASA in Table S1, or SASA by atoms in Table S2.

In addition to the globular features detected from the protein, the secondary structure propensities were also investigated. For the non-phosphorylated MAP2C trajectories (Figure S5), we see sporadic appearances of PPII helices, random coils, and a strong appearance of an α-helix in the MAP2C/H trajectory. Among the phosphorylated MAP2C trajectories (Figure S6), there is a noticeable and persistent turn moiety forming in MAP2C/B, and a transient α-helix in MAP2C/C, observed in the exact location as in MAP2C/H. The hTH1 trajectory has little or no significant appearance of a secondary structure, either persistent or

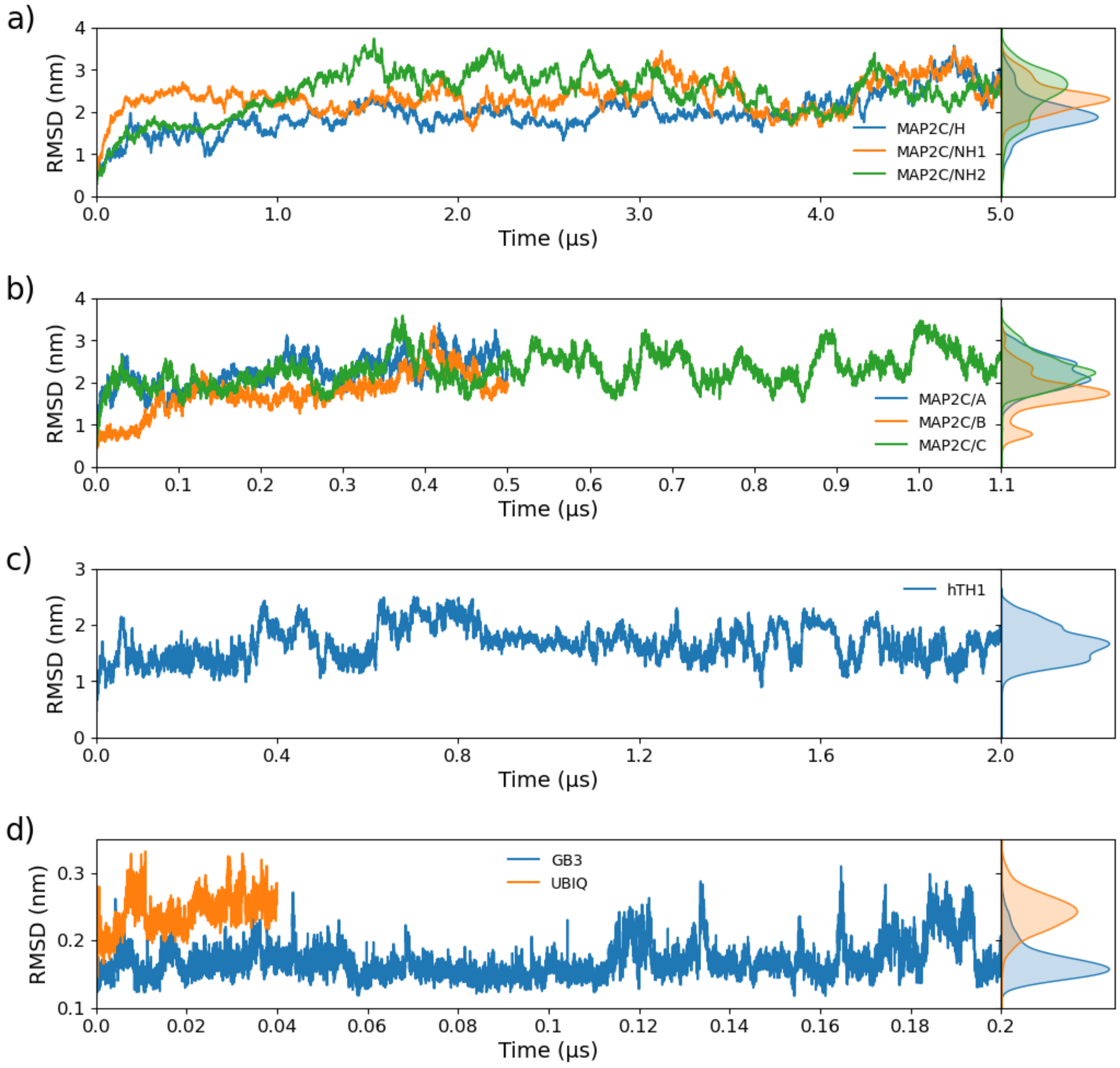

Figure S2: RMSD computed for all trajectories in the non-phosphorylated (a) and phosphorylated (b) MAP2C, hTH1 (c), and ordered (GB3/UBIQ) trajectories (d).

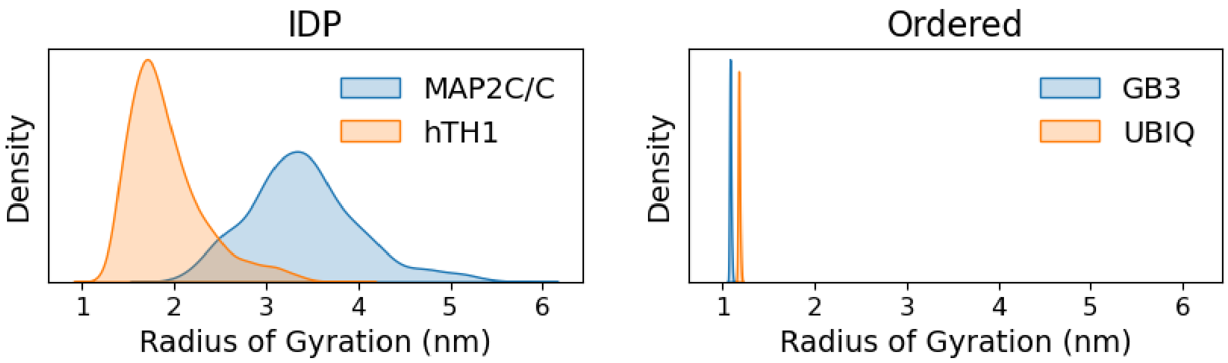

Figure S3: Radius of Gyration distribution for disordered (left) and ordered (right) trajectories.

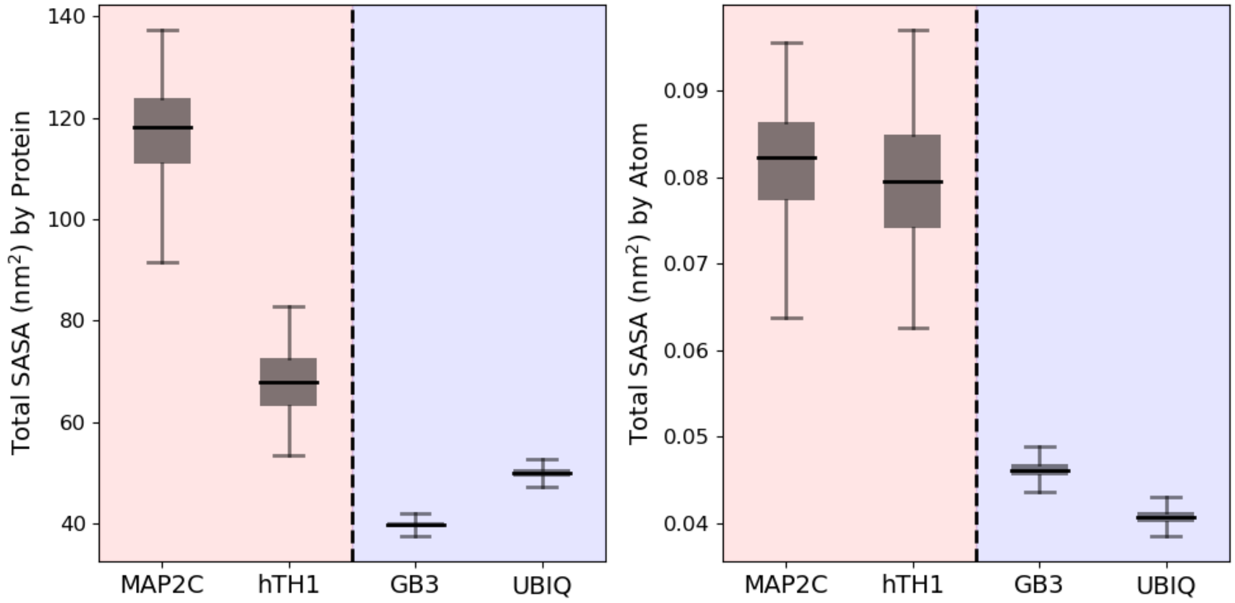

Figure S4: The total SASA computed by protein (a) and per atom (b) in the trajectories investigated among the ordered (blue) and disordered (red) proteins simulated.

transient (Figure S7), contrasting heavily from the DSSPs obtained for the ordered proteins, GB3 and Ubiquitin (Figure S8). The persistence and localization of poly-proline type 2 (PP2) helices were also computed for each trajectory in MAP2C (Figure S9).

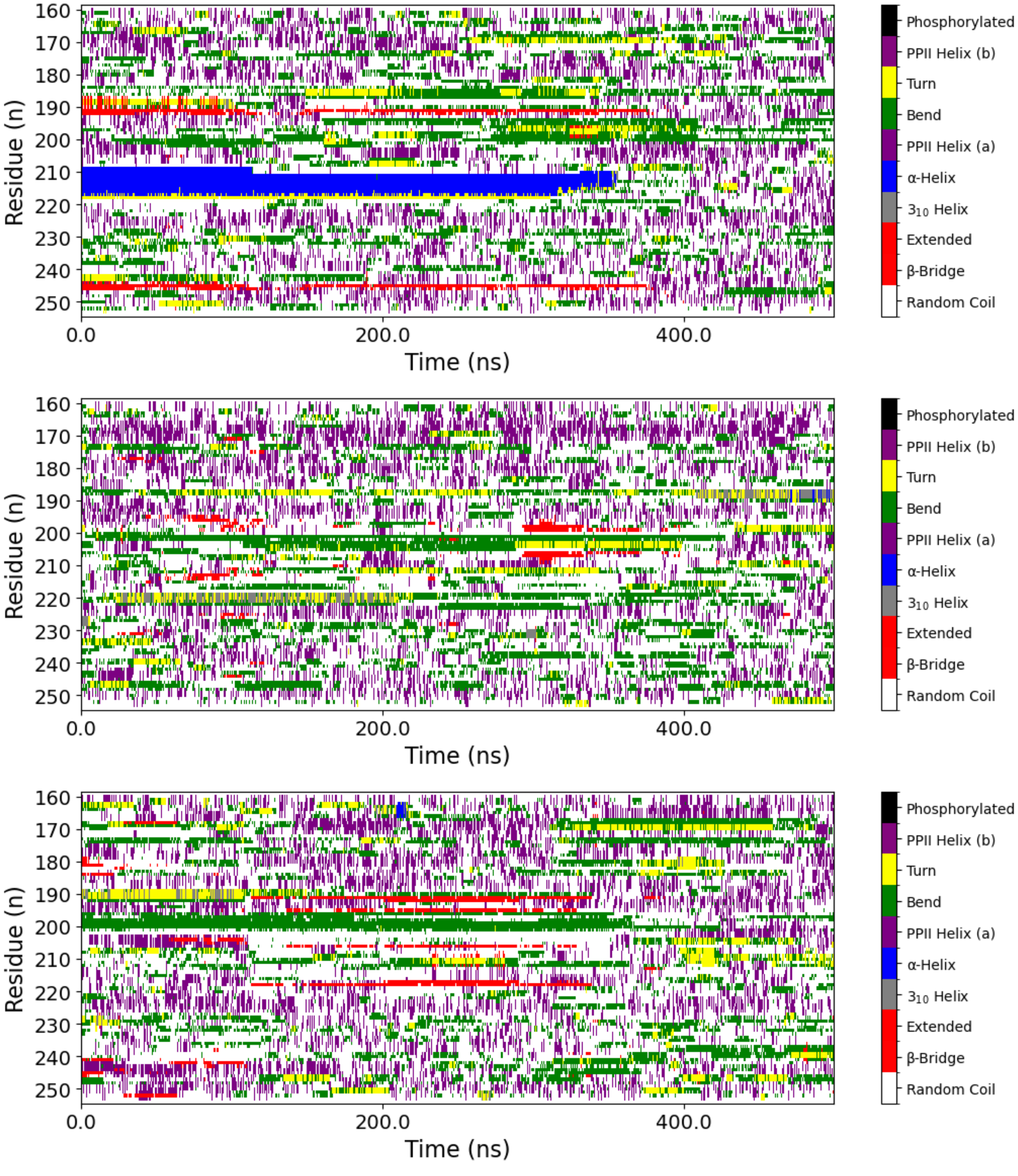

Figure S5: DSSP plots for the non-phosphorylated MAP2C trajectories; MAP2C/H, MAP2C/NH1, and MAP2C/NH2 (top to bottom).

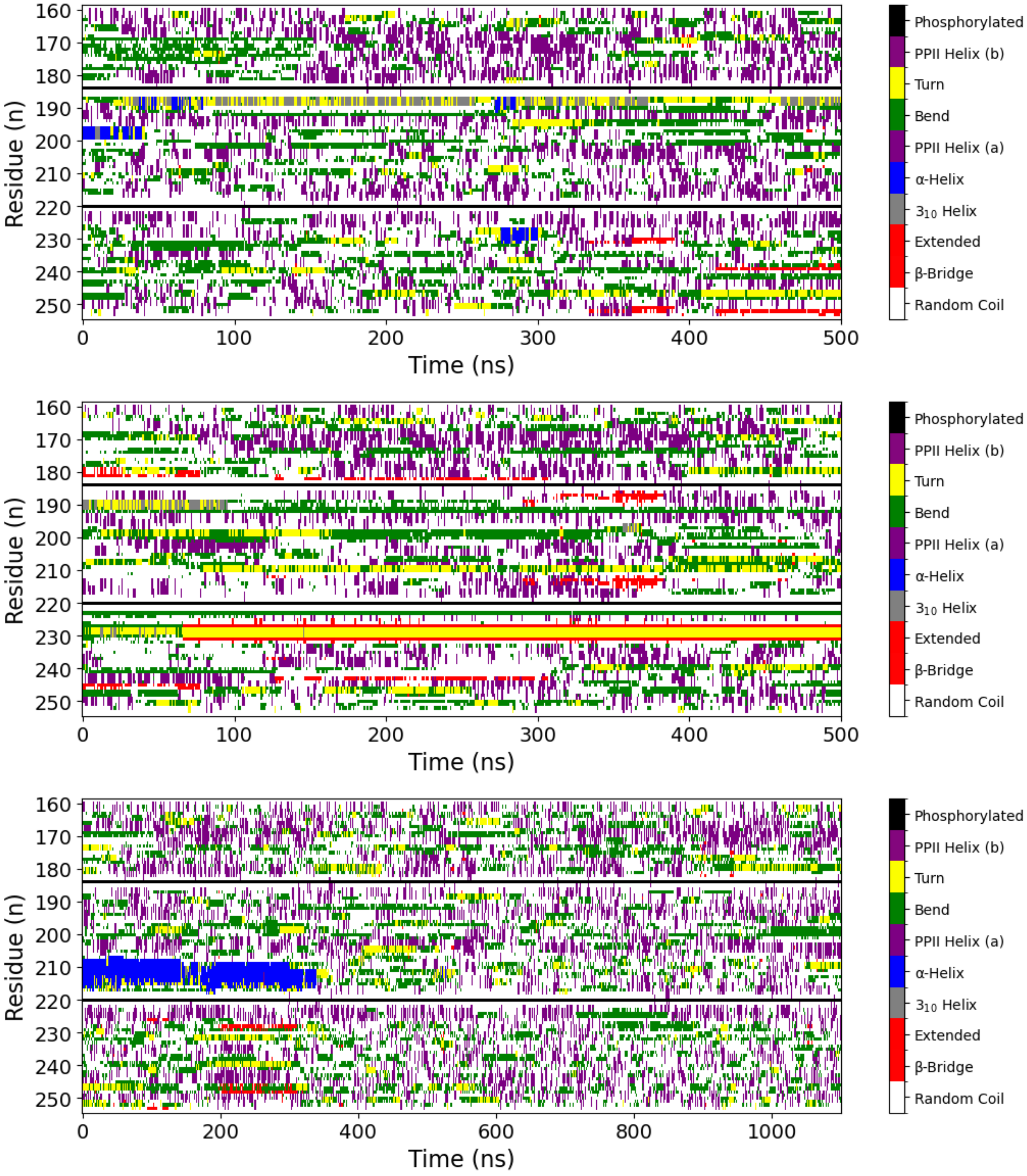

Figure S6: DSSP plots for the phosphorylated MAP2C trajectories; (top to bottom) MAP2C/A, MAP2C/B, and MAP2C/C.

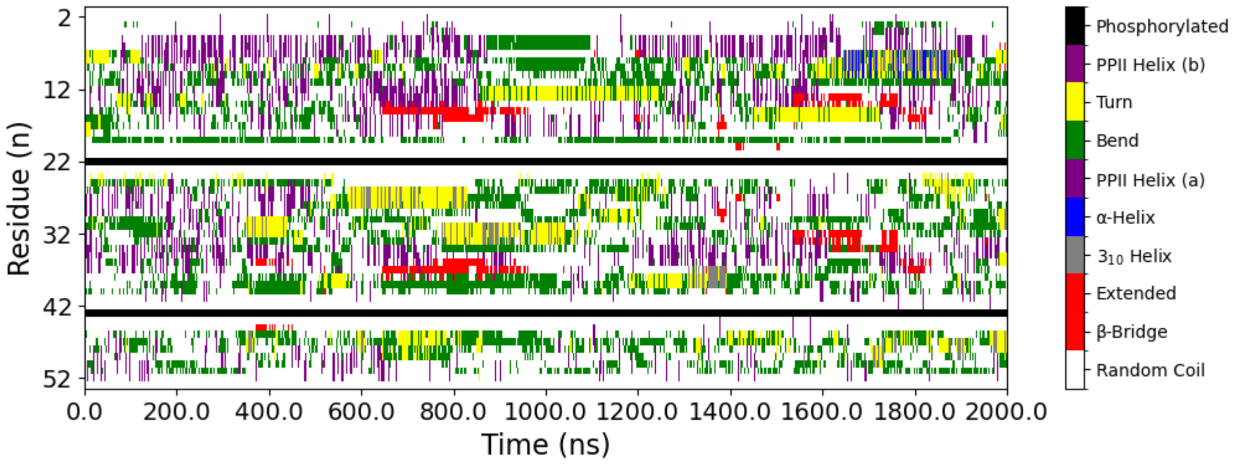

Figure S7: DSSP plots for the hTH1 trajectory with phosphorylated residues highlighted.

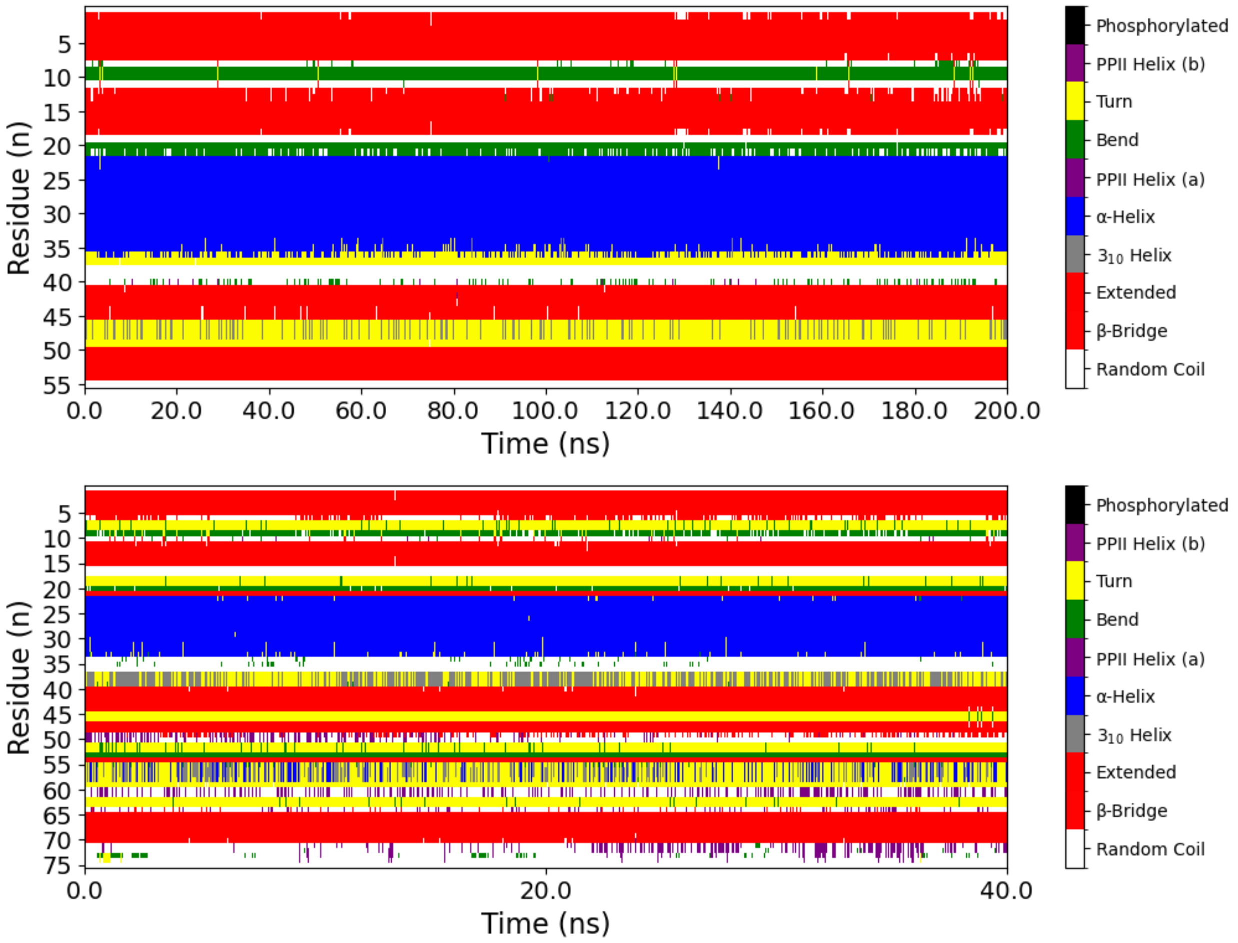

Figure S8: DSSP plots for the GB3 (top) and UBIQ (bottom) trajectories.

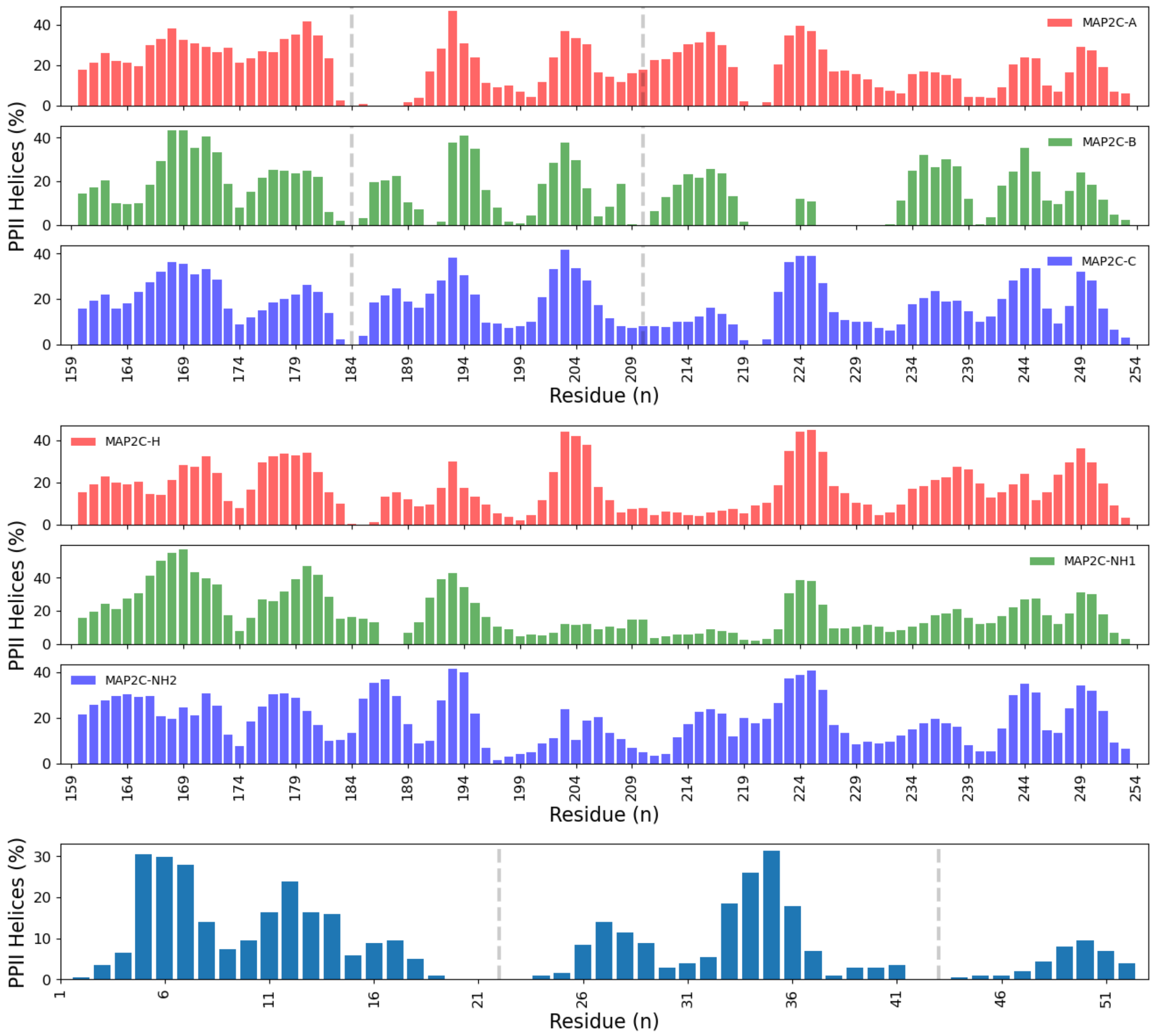

Figure S9: The percent (%) appearance of PPII helices in each of the disordered protein trajectories; MAP2C (both phosphorylated and non-phosphorylated), and hTH1 (bottom) by residue.

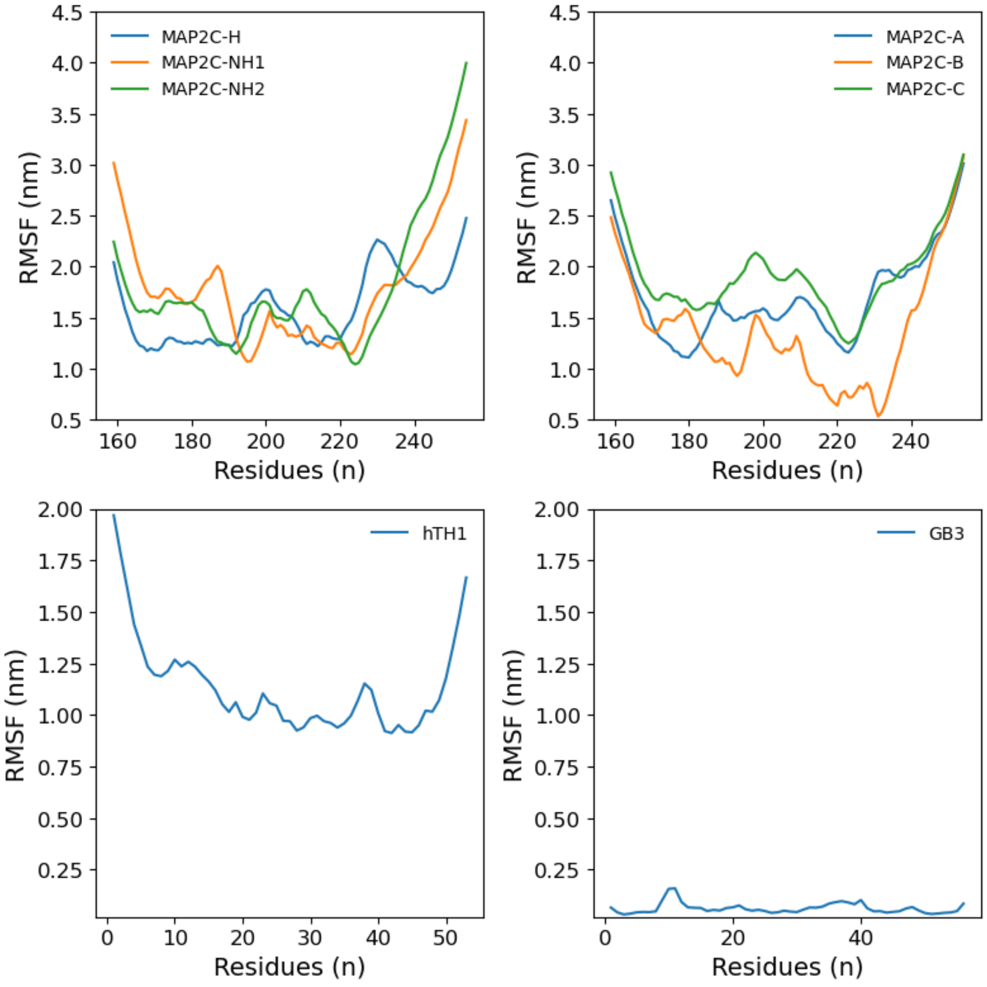

Figure S10: RMSF plots for all trajectories by residue computed based on the C' atom, demonstrating the extreme amounts of fluctuations experienced by IDPs, not exhibited in OPs.

Table S1: Computed solvent-accessible surface area (SASA) per protein means ($\bar{x}$), standard deviation (σ), kurtosis, skew, and variance from each respective trajectories

$SASA_{sum}$	$\bar{x}$ (nm^2)	σ	Kurtosis	Skew	Variance
MAP2C-H	114.4	8.4	-0.6	0.6	70.9
MAP2C-NH1	118.4	5.6	-0.7	0.1	31.5
MAP2C-NH2	115.1	7.2	0.4	-0.5	52.3
MAP2C-A	121.4	4.8	-0.5	0.0	23.3
MAP2C-B	107.0	6.3	-0.6	0.0	39.8
MAP2C-C	122.7	5.7	-0.3	-0.4	33.0
hTH1	68.0	5.8	-0.9	0.0	33.5
GB3	39.9	0.9	0.3	0.6	0.8
UBIQ	50.1	1.1	0.2	0.3	1.2

Table S2: Computed solvent-accessible surface area (SASA) per atom means ($\bar{x}$), standard deviation (σ), kurtosis, skew, and variance from each respective trajectories

$SASA_{atom}$	$\bar{x}$ (nm^2)	σ	Kurtosis	Skew	Variance
MAP2C/H	0.080	0.006	-0.646	0.644	0.000
MAP2C/NH1	0.083	0.004	-0.679	0.076	0.000
MAP2C/NH2	0.081	0.005	0.434	-0.453	0.000
MAP2C/A	0.085	0.003	-0.545	-0.034	0.000
MAP2C/B	0.074	0.004	-0.630	-0.033	0.000
MAP2C/C	0.085	0.004	-0.342	-0.411	0.000
hTH1	0.080	0.007	-0.931	0.018	0.000
GB3	0.046	0.001	0.317	0.589	0.000
UBIQ	0.041	0.001	0.224	0.327	0.000

S2 Chemical Shift Prediction Tools

Chemical Shift Predictions. Each CSP was evaluated by averaging and comparing to experimental through two metrics: root-mean-squared error (RMSE) and the coefficient of determination (R^2). The range of the data greatly influences RMSE values, as large ranges (e.g., C_β) often result in high RMSE regardless of the quality of the model. Additionally, small ranges (e.g., H_α) result in a low error (RMSE) but give very poor R^2 values. As such, a combination of both metrics is considered when assessing the performances, as RMSE indicates accuracy, while R^2 gives a measure of linearity. R^2 can be computed multiple ways,[1] but for this project we used the equation,

$$R^2 = 1 - \frac{\sum(x_i - \hat{x}_i)^2}{\sum(x_i - \bar{x}_i)^2} \quad (1)$$

where the top summation represents the sum of squares of the residuals, and the bottom summation gives the total sum of squares. This allows for values between -1 and 1.

For hTH1, Sparta+ performed relatively well in terms of linearity for the C_α, C_β, and N atoms (Table S3) but has relatively poor performances for C', H_α, and H_N CSs. ShiftX performed slightly better for the C', C_α, C_β, and H_α CSs although Sparta+ produced better agreement for the H_N and N atoms. Extracting conformations from the first half of the trajectory (1 μs) to the complete trajectory shows a slight improvement in the CS agreements for all atoms, as can be seen in Table S3 and Figure S11. The H_N R^2 improved from 0.22 to 0.26 by extending the trajectory from 1 to 2 μs. The slight improvement suggests that additional structures may exist that can be sampled from a longer trajectory or by using alternative sampling techniques.

Prosecco had the best agreement among the CSPs. However, it is limited to predictions based on amino acid sequences, which are not dynamic and thus are incapable of determining micro-states or generating conformational ensembles. Sparta+ and ShiftX ignore proline N atoms due to challenges in assigning them experimentally, as they require tailored hetero-nuclear NMR experiments.[2] ProCS-15 yielded the poorest agreement due to limitations from the DFT parameterized prediction tools, particularly in protic solvents (Table S3). Regres-

Table S3: R^2 correlation values between experimentally obtained CSs and predicted CSs using different parameters for the hTH1 protein, RMSE in parenthesis (). 5,000 frames were extracted for SEs from trajectory length t.

**Method**	**t**	$\mathbf{H_N}$	**N**	$\mathbf{H_\alpha}$	$\mathbf{C_\alpha}$	$\mathbf{C_\beta}$	$\mathbf{C'}$
Prosecco		0.23(0.22)	0.88(1.41)	0.81(0.18)	0.98(1.50)	1.00(1.09)	0.78(0.67)
Sparta+	1 μs	0.22(0.26)	0.86(1.32)	0.14(0.39)	0.91(1.87)	1.00(1.16)	0.66(0.93)
Sparta+	2 μs	0.26(0.25)	0.87(1.30)	0.14(0.39)	0.92(1.86)	1.00(1.14)	0.70(0.92)
ShiftX	1 μs	0.18(0.29)	0.85(1.62)	0.19(0.35)	0.91(1.90)	1.00(0.90)	0.72(0.93)
ShiftX	2 μs	0.22(0.26)	0.85(1.61)	0.19(0.35)	0.92(1.89)	1.00(0.89)	0.74(0.93)
ProCS-15	1 μs	0.00(0.61)	0.59(13.3)	0.06(1.17)	0.84(3.31)	0.99(1.82)	0.40(16.9)
ProCS-15	2 μs	0.00(0.62)	0.60(13.3)	0.04(1.17)	0.85(3.28)	0.99(1.81)	0.43(17.0)

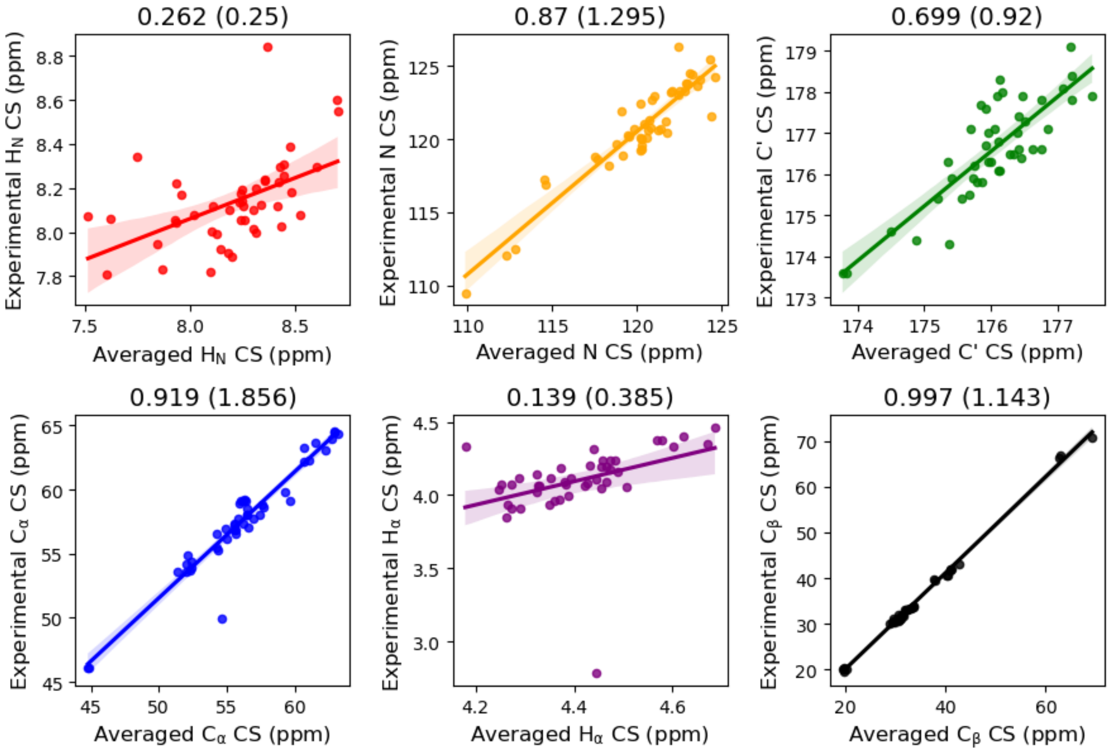

Figure S11: Chemical shift agreement plots using Sparta+ for hTH1 trajectory for each of the atoms with the r^2 value and RMSE () from experimental.

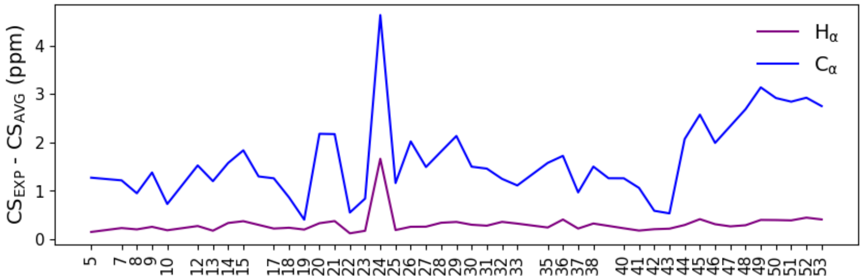

Figure S12: Difference in the H_α and C_α Sparta+ chemical shifts between the averaged Sparta+ calculations for hTH1 and the experimental by residue.

sion plots for the ProCS-15, ShiftX and Prosecco for the hTH1 agreements can be seen in the supporting information (Figures S11/S13/S15 respectively). All methods show significant deviation from experimental values for both C_α and H_α atoms on residue Leu^{24}, which might suggest an artifact in the trajectory, or an interaction not observed experimentally.

For the phosphorylated MAP2C trajectories, experimental CSs were available only for H_N and N. [3] There were three significant outliers in the H_N regression plots of MAP2C/A (Figure

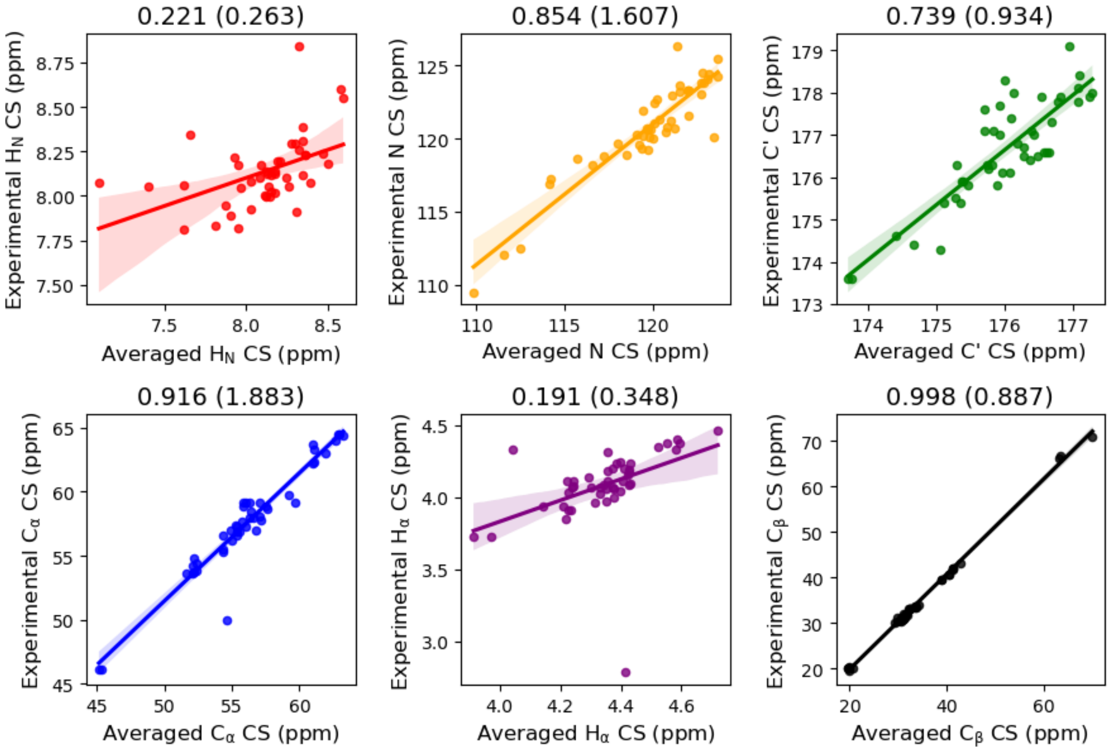

Figure S13: Chemical shift agreement plots using ShiftX2 for hTH1 trajectory for each of the atoms with the r^2 value and RMSE () from experimental.

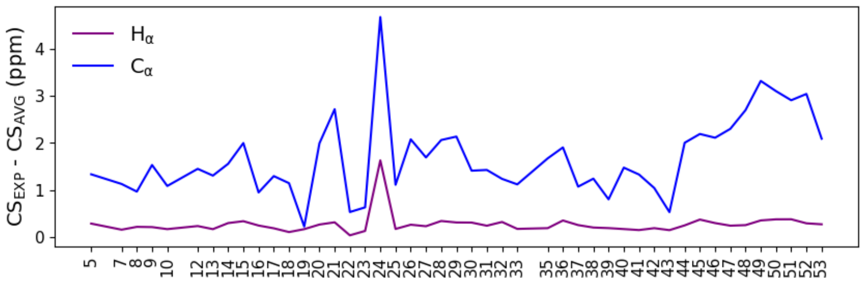

Figure S14: Difference in the H_α and C_α ShiftX2 chemical shifts between the averaged Sparta+ calculations for hTH1 and the experimental by residue.

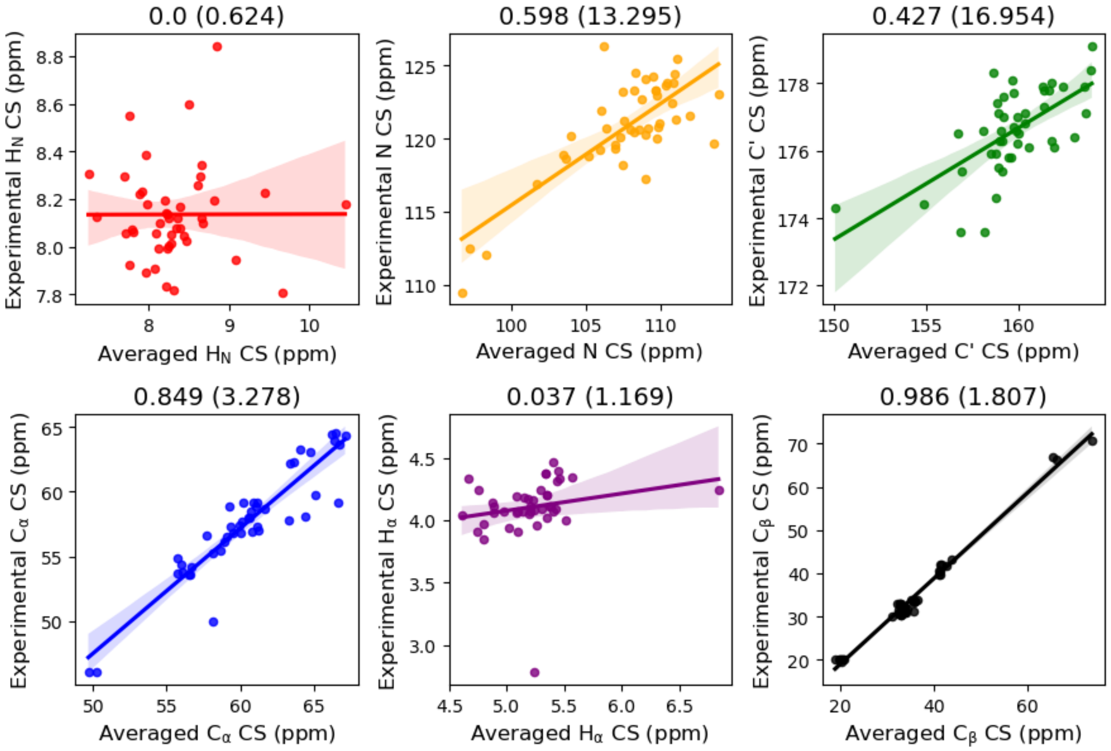

Figure S15: Chemical shift agreement plots using ProCS15 for hTH1 trajectory for each of the atoms with the r^2 value and RMSE () from experimental.

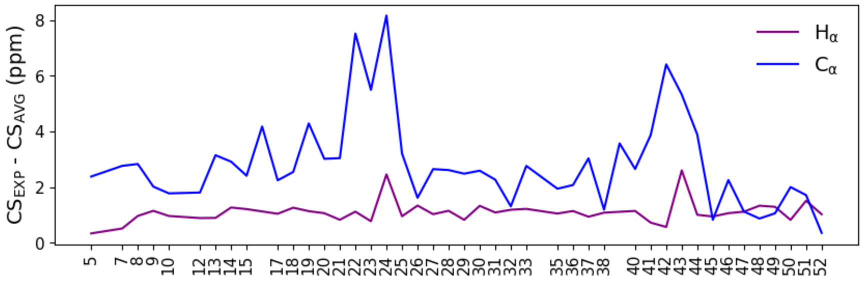

Figure S16: Difference in the H_α and C_α ProCS15 chemical shifts between the averaged Sparta+ calculations for hTH1 and the experimental by residue.

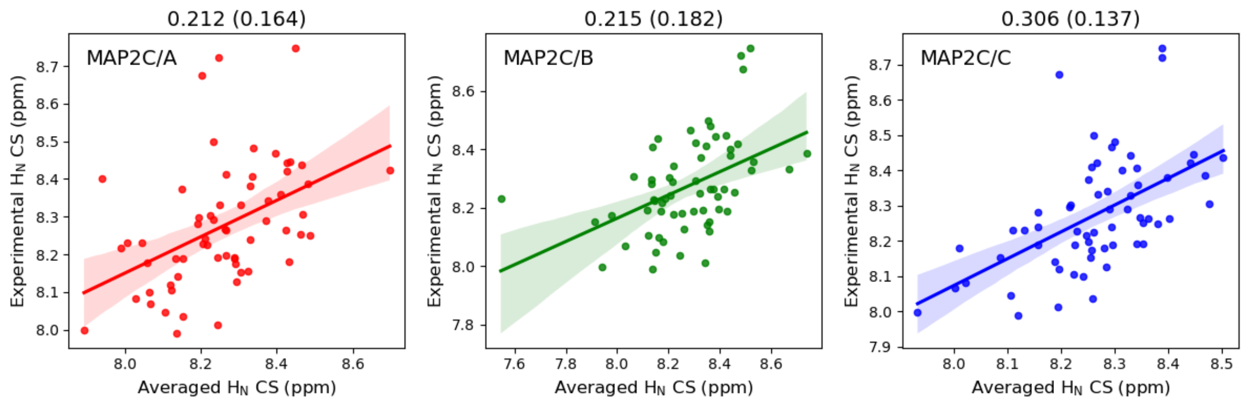

Figure S17: Regression plots for Sparta+ predicted H_N (top) and CSs in phosphorylated MAP2C including the R^2 value and the RMSE in parenthesis () for MAP2C/A (left), MAP2C/B (middle), and MAP2C/C (right).

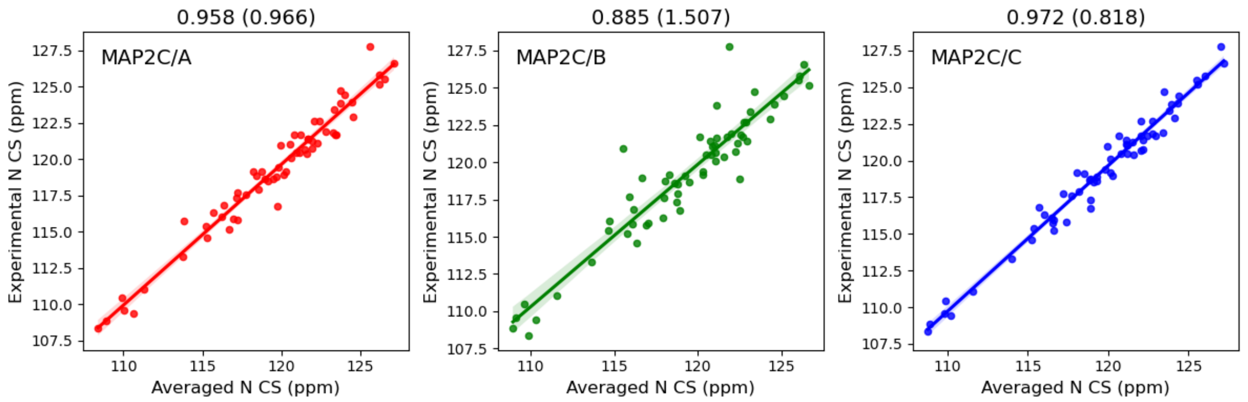

Figure S18: Chemical shift agreement plots using Sparta+ for the phosphorylated MAP2C trajectory for N atoms with the r^2 value and RMSE () from experimental.

S17a) and MAP2C/C (Figure S17c); Ser^{184}, Tyr^{220} and Arg^{221}. Each of these is a hydrophilic residue, and two were phosphorylated, which demonstrates that Sparta+ is unable to account for the phosphorylation using its current methodology. These three residues are not as evident in MAP2C/B, although there were four residues which produced large standard deviations: Phe^{207} (2.68 ppm), Tyr^{252} (1.99 ppm), Phe^{208} (1.67 ppm), Ser^{253} (1.63 ppm) as seen in Figure S17b. The MAP2C/C trajectory performed best, followed by MAP2C/A and MAP2C/B, both in the RMSE and their R^2 values. A comparison by the absolute error using RMSE produces a slightly different trend, although MAP2C/C is still paramount. ShiftX produces similar results, as seen in Table S4.

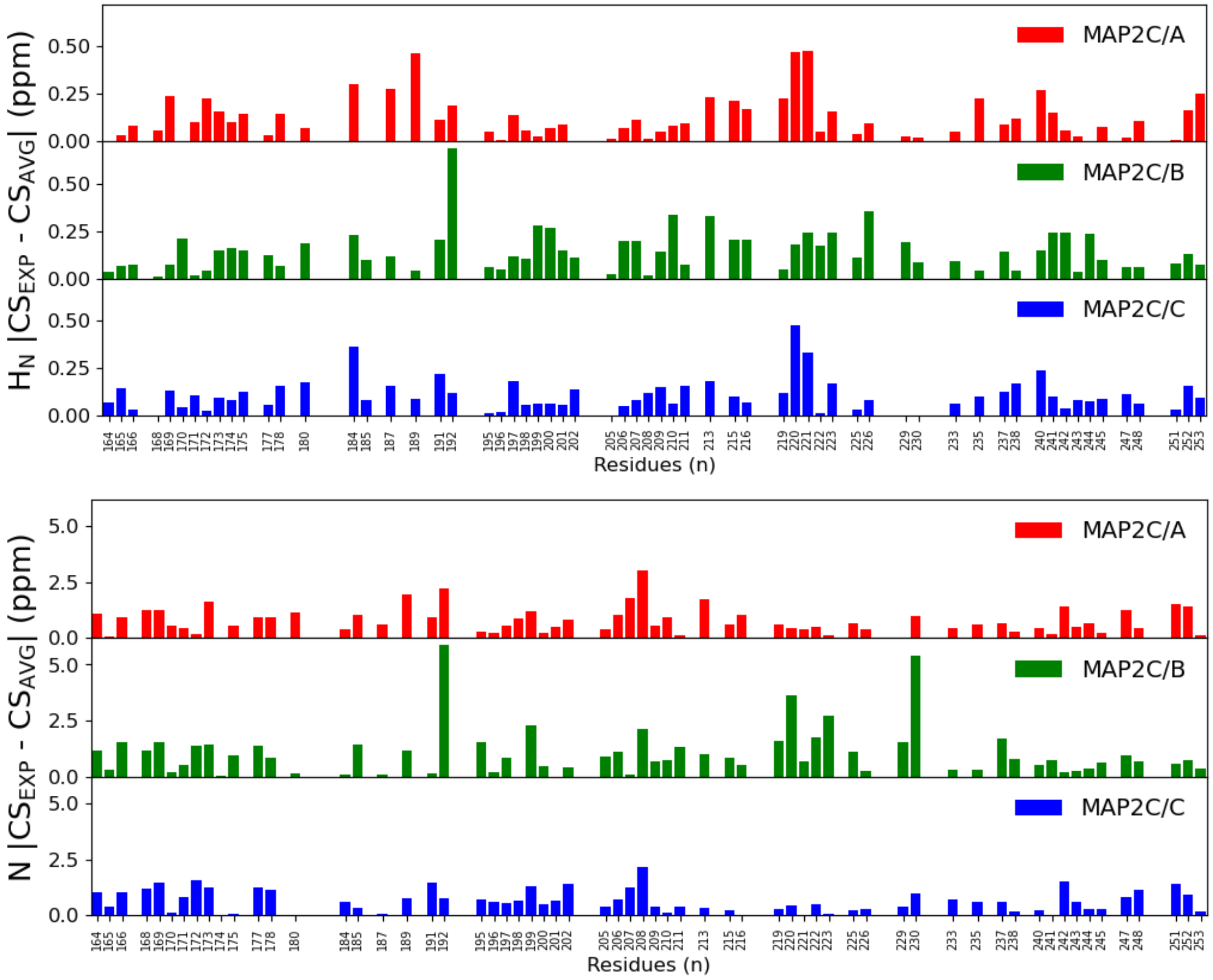

Figure S19: Chemical shift agreement plots using Sparta+ for the phosphorylated MAP2C trajectory for H_N (top) and N (bottom) atoms with the r^2 value and RMSE () from experimental.

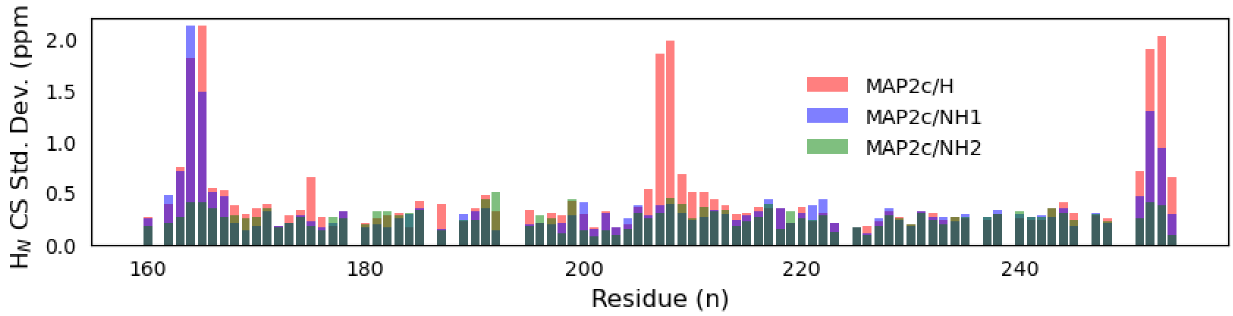

Figure S20: Standard deviations of the chemical shifts from non-phosphorylated MAP2C/H (red), MAP2C/NH1 (blue), and MAP2C/NH2 (green).

Table S4: R^2 values for MAP2C phosphorylated trajectory SEs using various CS prediction tools, with frames extracted sequentially from each trajectory. RMSE values are included in parenthesis () in ppm.

**Trajectory**	**CSP**	$\mathbf{H_N}$	**N**
MAP2C/A	Sparta+	0.21 (0.16)	0.96 (0.97)
	ShiftX	0.17 (0.18)	0.95 (1.0)
	ProCS-15	0.02 (0.72)	0.81 (13)
MAP2C/B	Sparta+	0.22 (0.18)	0.88 (1.5)
	ShiftX	0.20 (0.19)	0.86 (1.6)
	ProCS-15	0.01 (0.62)	0.59 (14)
MAP2C/C	Sparta+	0.31 (0.14)	0.97 (0.82)
	ShiftX	0.30 (0.14)	0.96 (0.92)
	ProCS-15	0.03 (0.72)	0.85 (13)

In the MAP2C non-phosphorylated trajectories, the best performing atoms according to their R^2 values are C_β, C_α, C', N, H_α, and H_N, although the trend for the RMSE are H_α, H_N, C_β, C', C_α, and N (Table S5). For comparisons between atoms, R^2 is more apt to describe the performance, as it details well the relative performance and not absolute errors generated from the models. There were six residues which produced significant standard deviations from the other residues in MAP2C/H, jumping from 0.75 to 1.81 ppm and increasing; Lys^{165} (2.13 ppm), Ser^{253} (2.03 ppm), Ser^{208} (1.98 ppm), Tyr^{252} (1.90 ppm), Phe^{207} (1.85 ppm), and Phe^{164} (1.81 ppm) in descending order (Figure S20). By contrast, only four of these residues are particularly divergent in the MAP2C/NH1 trajectory (Figure S20); Phe^{164} (2.12 ppm), Lys^{165} (1.47 ppm), Tyr^{252} (1.30 ppm), and Ser^{253} (0.94 ppm). Among the non-phosphorylated trajectories, the trend seems to be that MAP2C/NH2 performs the best, followed by MAP2C/H and MAP2C/NH1. This provides evidence that the non-phosphorylated MAP2C protein may not contain a strong presence of helical structure, in deference to the phosphorylated samples, although further investigations are warranted.

For the ordered proteins (GB3 and Ubiquitin), there is a similar trend in the prediction software performances among the atoms (Table S6). No experimental CSs were collected for C' with GB3, although all other atoms produced outstanding agreements in terms of RMSE and R^2, barring H_N. The performance in each instance is improved compared to the

Table S5: R^2 correlation values with experimental chemical shifts for MAP2C non-phosphorylated trajectories with various chemical shift predictors. Sequential ensembles were collected every 100 ps, or 5,000 frames each. RMSE is also calculated for comparison in parenthesis ().

**Protein**	**Method**	$\mathbf{H_N}$	**N**	$\mathbf{H_\alpha}$	$\mathbf{C_\alpha}$	$\mathbf{C_\beta}$	$\mathbf{C'}$
MAP2C	Prosecco	0.68 (0.1)	0.32 (6.5)	0.95 (0.0)	1.00(0.2)	1.00 (0.2)	0.98 (0.2)
H	Sparta+	0.23 (0.2)	0.94 (1.2)	0.51 (0.1)	0.98 (0.8)	1.00 (0.6)	0.86 (0.5)
	ShiftX	0.12 (0.2)	0.90 (1.5)	0.57 (0.1)	0.97 (0.9)	1.00 (0.4)	0.85 (0.6)
	ProCS-15	0.08 (0.5)	0.21 (12.7)	0.15 (0.8)	0.94 (4.1)	0.99 (2.8)	0.44 (16.0)
NH1	Sparta+	0.20 (0.2)	0.95 (1.1)	0.49 (0.1)	0.98 (0.8)	1.00 (0.5)	0.90 (0.5)
	ShiftX	0.10 (0.2)	0.94 (1.2)	0.62 (0.1)	0.98 (0.9)	1.00 (0.4)	0.91 (0.6)
	ProCS-15	0.10 (0.5)	0.24 (12.9)	0.18 (0.8)	0.94 (3.9)	1.00 (2.7)	0.47 (16.2)
NH2	Sparta+	0.24 (0.1)	0.95 (1.0)	0.57 (0.1)	0.99 (0.5)	1.00 (0.5)	0.92 (0.4)
	ShiftX	0.14 (0.1)	0.95 (1.0)	0.70 (0.1)	1.00 (0.4)	1.00 (0.4)	0.92 (0.6)
	ProCS-15	0.08 (0.4)	0.25 (12.3)	0.15 (0.8)	0.95 (3.8)	1.00 (2.6)	0.45 (16.2)

IDPs, with ShiftX slightly outperforming Sparta+ for every atom except for C_α. Despite significantly improved C_α performance, the C' CSs for Ubiquitin have a similar agreement value compared to the IDPs. This suggests that for some nuclei, the order in the protein has less influence on a CS prediction tool's ability to predict CSs than others.

Table S6: CS agreements (R^2 values) obtained from predictions of ordered proteins GB3 and UBIQ, averaged SEs obtained from 5000 frames at time steps of 100 ps.

Protein	Method	H_N	H_α	C_α	C_β	C'	N
GB3	Sparta+	0.70 (0.4)	0.81 (2.8)		0.97 (0.8)	0.91 (0.2)	1.00 (1.1)
	ShiftX	0.64 (0.5)	0.86 (2.6)		0.95 (1.0)	0.88 (0.3)	1.00 (1.1)
UBIQ	Sparta+	0.56 (0.4)	0.85 (2.3)	0.72 (1.1)	0.97 (0.8)	0.86 (0.2)	0.99 (1.1)
	ShiftX	0.55 (0.4)	0.85 (2.2)	0.70 (1.0)	0.97 (0.7)	0.84 (0.1)	0.99 (1.0)

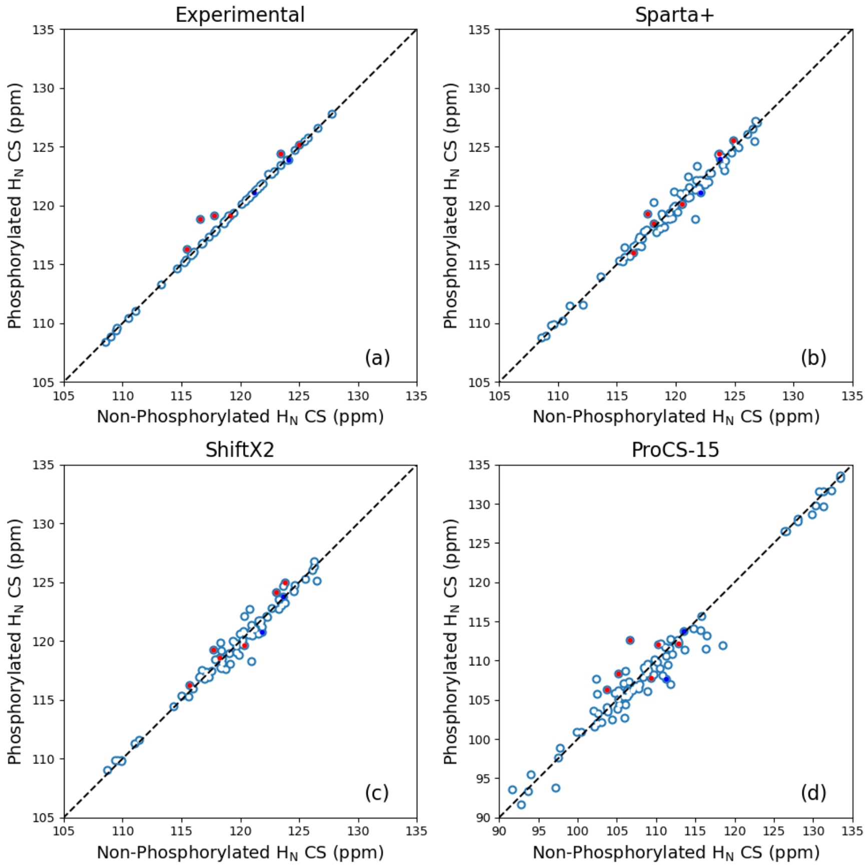

Figure S21: Influence of the phosphorylation of the protein on the ^{15}N CS by residue plotted for experimental (a), Sparta+ (b), ShiftX (c), and ProCS-15 (d) with specific shifted residues labeled.

S3 Sequential Ensembles

While CS prediction tools can be done on massive ensembles or even the complete set, quantum calculations become intractable when scaled to these levels. Additionally, if done properly, the generation of smaller ensembles can reduce noise from the system, identify key features or events, and allow for visualization of different conformational states of pluriform systems. Traditional ensembles are generated sequentially and equidistantly by extracting frames from the trajectory, and then the shifts are averaged as seen for CSs in Equation 2,

$$\bar{\delta}_{\mathrm{SE}} = \frac{1}{n}\sum_{i=1}^{n} \mathrm{CS}\Big(\Big\lfloor \frac{(0.5+i)*M}{n} \Big\rfloor \Big) \tag{2}$$

where M is the total size of the trajectory, n is the size of the sequential ensemble, and CS(j) represents the chemical shift at that particular time stamp. CS, in this case, is an already prepared array of chemical shifts generated for each frame of each trajectory. Using this method, the point at which the performance of the ensembles converges can be determined. Autocorrelation plots were computed for the chemical shifts from a single residue (Thr_{220}) showing that all values converge, although at different sizes of datasets. Figure S22a/b shows that the exact size required to achieve ideal results varies between atom types. For N CSs, the ensembles seem to converge within 100 frames, while $^{1}\mathrm{H}_{\mathrm{N}}$ can take hundreds, if not thousands, of frames to converge. While these larger ensembles may be helpful for rapid predictions, more in-depth analysis or quantum calculations become infeasible or prohibitively expensive. For this purpose, clustering techniques are applied to reduce noise and redundancy.

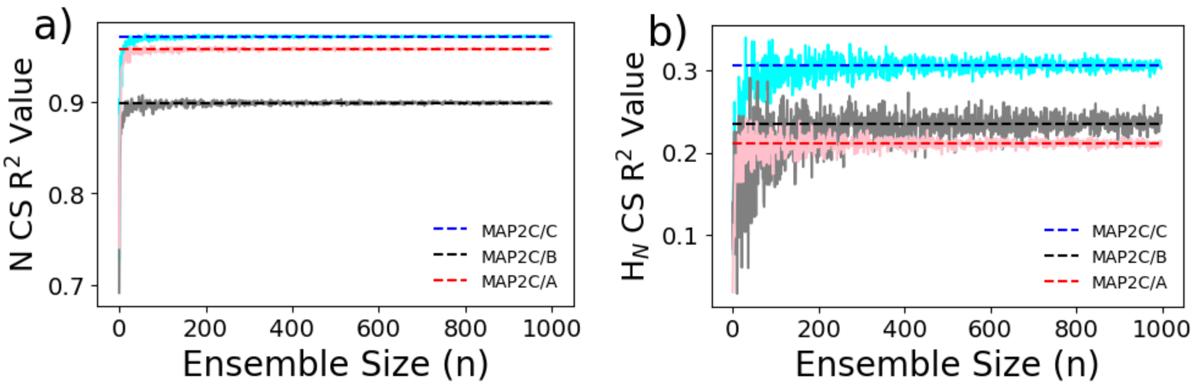

Figure S22: Performance of sequential ensembles averaged equidistantly from Equation 2. MAP2C/A, MAP2C/B, and MAP2C/C are shown in red, black, and blue, respectively, for N (a) and H_N (b) Sparta+ predicted CS R^2 agreements at different sized ensembles (n).

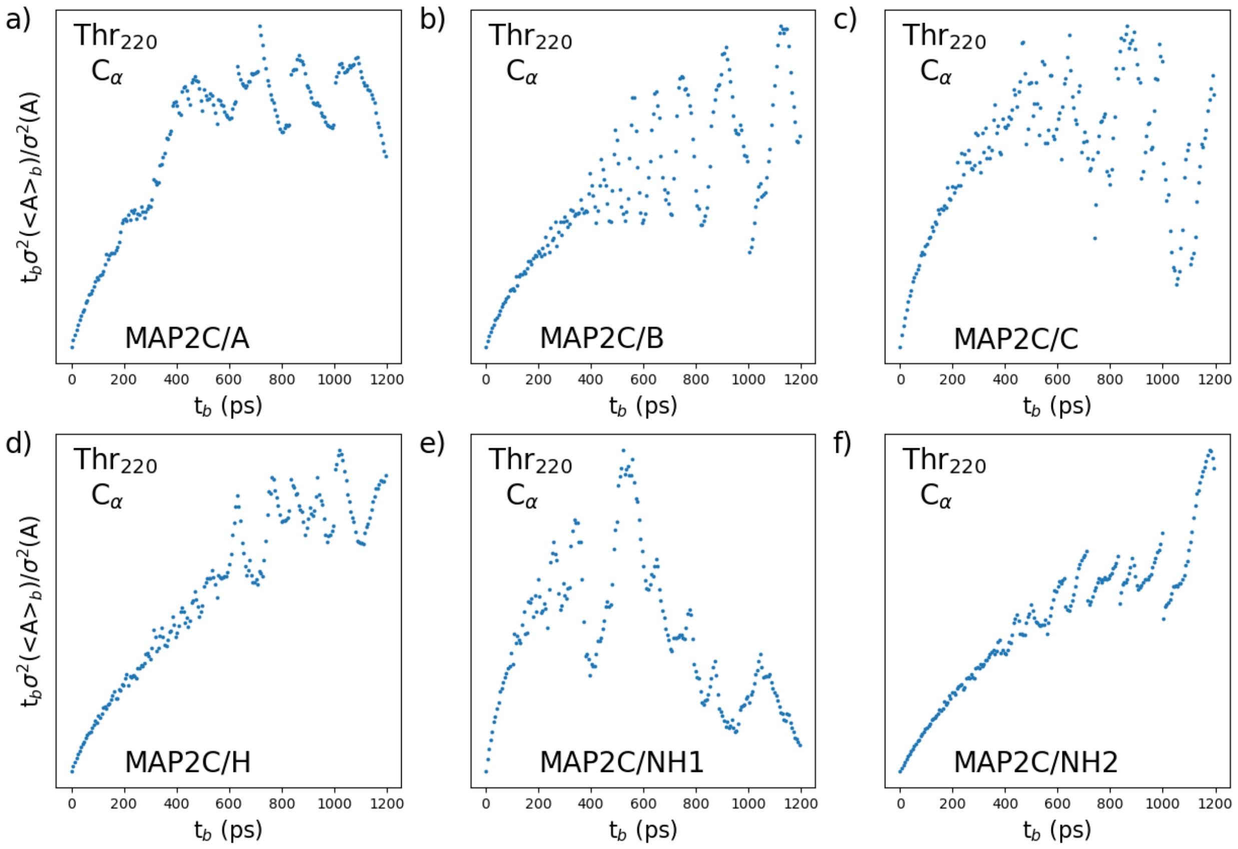

Figure S23: Autocorrelation plots for each of the MAP2C trajectories based on the C_α chemical shift from the Thr_{220} residue.

S4 GROMOS Clustered Ensembles

Table S7: Difference between the ensemble performance of the CSs from GB3 based on GROMOS RMSD clustering compared to experimental data at different RMSD cutoffs and the sequential ensembles of the same size.

**Cutoff**	**Size**	**N**	$\mathbf{H_N}$	$\mathbf{H_\alpha}$	$\mathbf{C_\alpha}$	$\mathbf{C_\beta}$
0.045	7421	-0.013	-0.005	-0.004	-0.004	-0.005
0.047	5162	-0.018	-0.007	-0.005	-0.004	-0.008
0.048	4266	-0.023	-0.008	-0.005	-0.008	-0.010
0.049	3484	-0.023	-0.009	-0.006	-0.006	-0.011
0.05	2851	-0.027	-0.010	-0.006	-0.009	-0.009
0.055	1044	-0.029	-0.014	-0.010	-0.011	-0.012
0.06	427	-0.025	-0.016	-0.012	-0.013	-0.020
0.07	104	-0.036	-0.019	-0.009	-0.008	-0.027
0.08	32	0.069	-0.015	-0.015	-0.028	-0.005
0.09	15	-0.075	-0.015	-0.007	-0.021	-0.025
0.1	7	-0.039	0.019	0.002	0.008	0.060
0.15	3	-0.030	-0.020	0.015	-0.005	-0.168

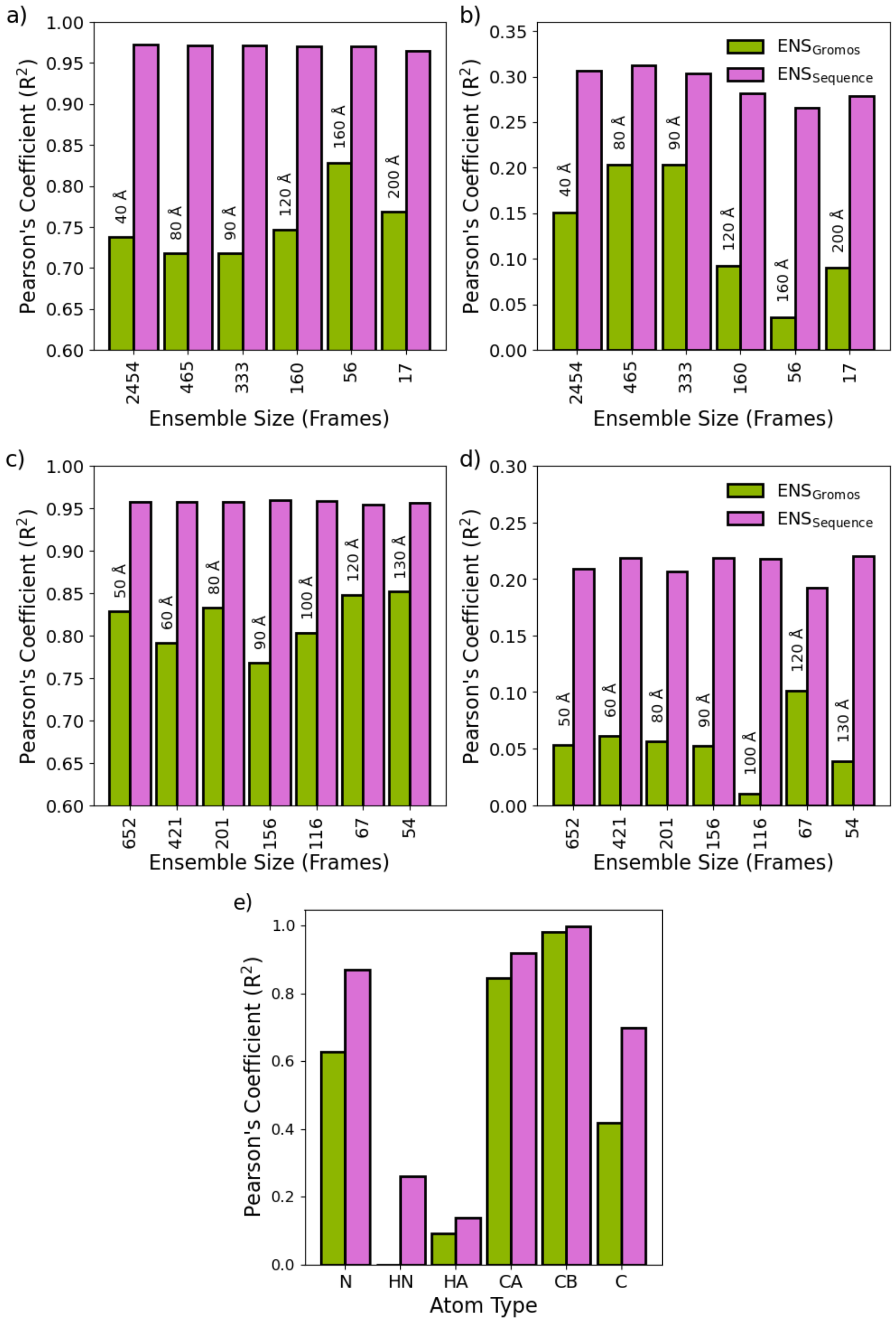

Figure S24: Comparisons of the performances for ensembles generated through the GROMOS algorithm in MAP2C/A for H_N (a) and N (b), MAP2C/C for H_N (a) and N (b), and all atom types in hTH1 (cutoff = 0.45 Å) to their respective ensembles generated sequentially.

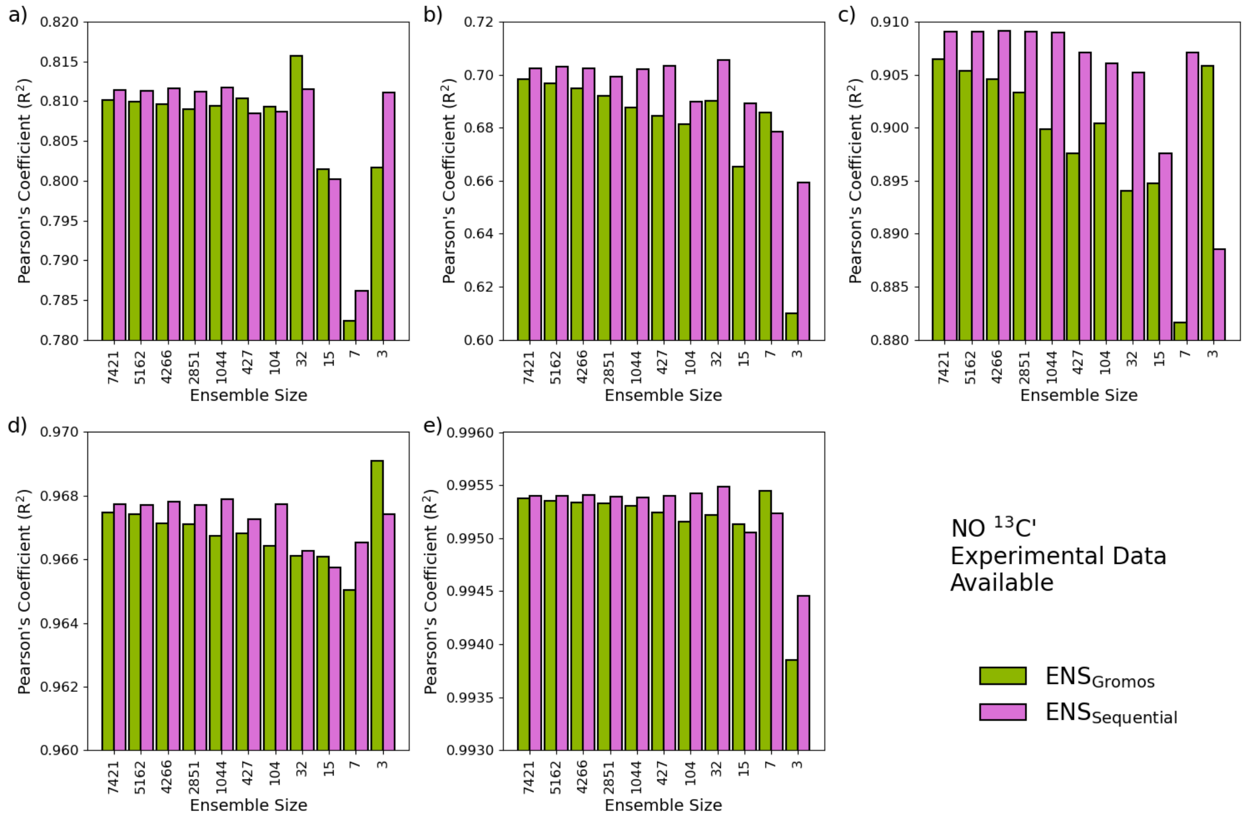

Figure S25: Comparison of ensembles' performances generated sequentially and through the GROMOS algorithm and for different atom types.

S5 Dimensionality Reduction

We first selected several DR techniques to implement to refine the procedure. Among linear techniques, we tested principal component analysis (PCA), linear discriminant analysis (LDA), and time-lagged independent component analysis (tICA). We eliminated LDA due to additional parameters that, in preliminary trials, did not improve feature selection or necessitate the need for differentiation between classes. We instead chose PCA and tICA, the first due to their ubiquitous implementation in multiple fields and versatility, and the second due to their consideration of properties as they propagate through time with time-lag parameters. LDA and PCA were implemented through *sklearn*, while tICA was implemented through deeptime. The resultant latent spaces for the different trajectories and different input features are described in Figure S26.

Among the nonlinear techniques, t-distributed stochastic neighbor embedding (tSNE) was tested as the baseline for nonlinear techniques, as well as unifold manifold approximation and projection (UMAP), kernel principal component analysis (kPCA), multidimensional scaling (MDS), Isomap, and Laplacian eigenmaps. Of these techniques, due to the complexity involved in nonlinear techniques and the additional parameterization (through hyperparameters) required for the proper implementation of the methods, only tSNE was evaluated for the generation of a manifold latent conformational space. The Python package sklearn was used to implement MDS, kPCA, tSNE, Isomap, and Laplacian eigenmaps with the independent Python package UMAP. Incorporating a well-chosen perplexity value into tSNE is imperative, as it enables the algorithm to effectively reduce the dimensionality of high-dimensional datasets. Given that this study does not focus on nonlinear dimensionality reduction, tSNE was selected because of its widespread use and ease of implementation.

In addition to choosing the appropriate DR method, the selection of an appropriate input fingerprint is equally important in generating an accurate representation of the latent space. An input fingerprint is a higher-dimensional dataset that captures the key structural fluctuations and conformational changes of a molecule during molecular dynamics simulations.

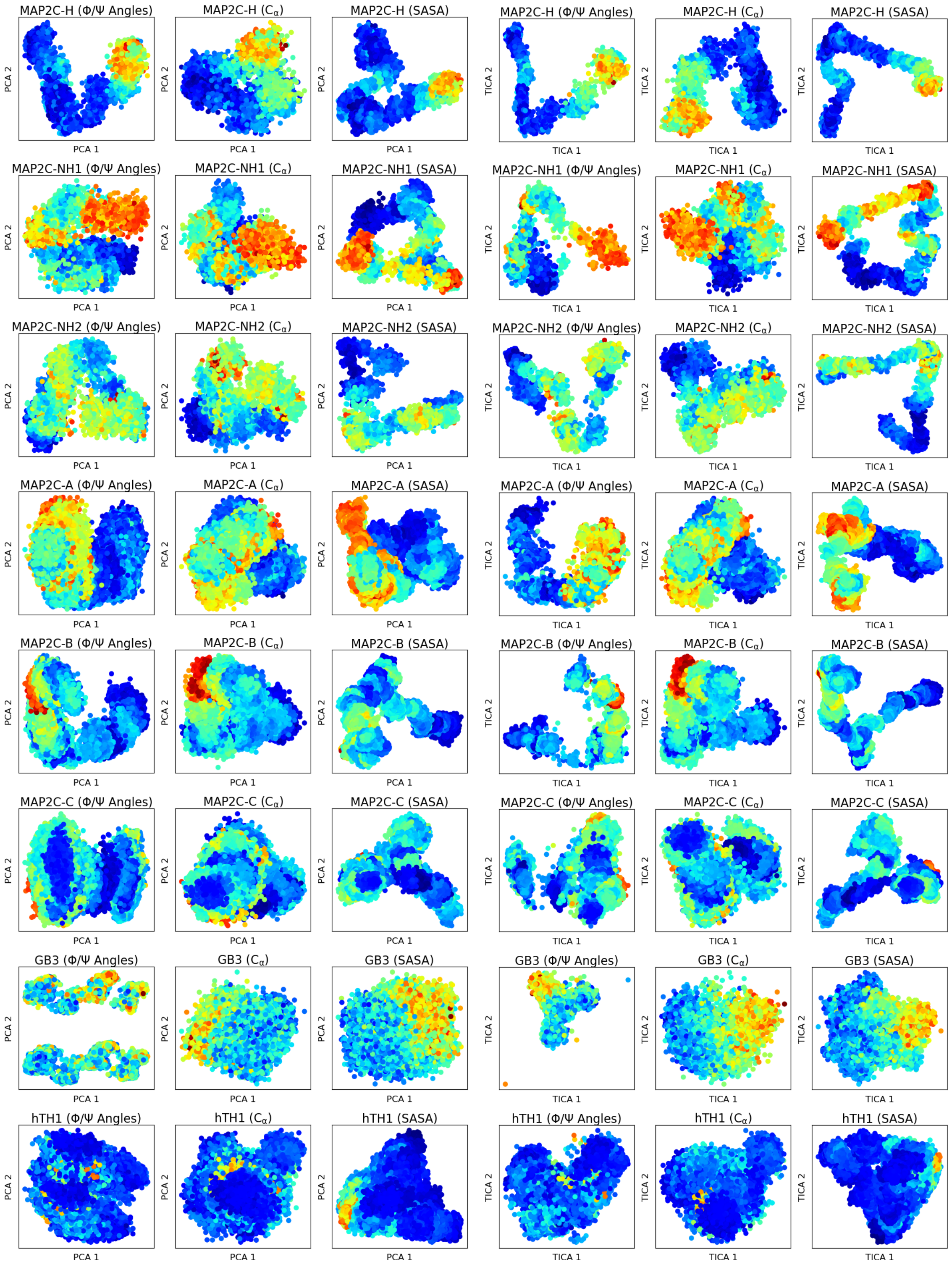

Figure S26: Dimensionality reductions on all trajectories, using linear DR techniques (PCA and TICA) with three features of the trajectories (ϕ/ψ, C_α, and SASA) plotted with R_G as the colors.

Several input fingerprints were proposed for use in DR-based analyses, of which three have been incorporated in this investigation. These include dihedral ϕ and ψ angles, α carbon distances and angles, and solvent-accessible surface area, as described in the introduction. Selecting the appropriate input fingerprint is of utmost importance, as it determines the ability of the DR method to effectively capture the essential information from the molecular dynamics trajectories. Neglecting significant features or including unimportant noise can greatly impact the quality of the latent space and, ultimately, the accuracy of the DR method. Therefore, careful consideration must be given to selecting the most appropriate input fingerprint that can adequately capture the relevant features and discard irrelevant noise for a successful DR analysis in the context of molecular dynamics simulations. For example, incorporating SASA from selective atoms as fingerprints for a TICA DR produces different latent spaces, some more significant than others (Figure S29). Notably, the significance of the N SASA is negligible to the conformational landscapes in the proteins.

Table S8: Different clustered ensembles achieved from utilizing different features for the fingerprints of the dimensionality reduction, and clustering with hierarchical clustering compared for their averaged R_G, RMSD, SASA, and EE_{DIST}.

**Feature**†	**State**	**R_G (nm)**	**RMSD (nm)**	**SASA (nm^2)**	**EE_{dist} (nm)**
	α	3.26 ± 0.41	2.00 ± 0.28	116.44 ± 3.91	5.90 ± 1.12
**Phi/Psi**	$\alpha\beta$	2.54 ± 0.46	2.28 ± 0.14	119.44 ± 3.34	5.22 ± 1.07
SS = 0.513	β_1	3.26 ± 0.57	2.38 ± 0.38	125.42 ± 4.99	4.95 ± 1.52
DB = 0.634	β_2	3.83 ± 0.44	2.17 ± 0.23	126.02 ± 3.23	5.27 ± 1.34
	β_3	3.67 ± 0.67	2.58 ± 0.38	124.96 ± 3.22	5.14 ± 1.39
	α_1	3.43 ± 0.29	2.08 ± 0.21	117.72 ± 2.49	6.44 ± 1.00
**Dist/Ang**	α_2	3.19 ± 0.46	2.02 ± 0.29	116.64 ± 4.00	5.83 ± 1.12
SS = 0.396	β_1	3.61 ± 0.62	2.63 ± 0.36	124.92 ± 3.56	5.22 ± 1.40
DB = 0.684	β_2	3.62 ± 0.79	2.49 ± 0.39	125.08 ± 3.30	4.95 ± 1.44
	β_3	3.40 ± 0.60	2.32 ± 0.35	125.48 ± 4.62	5.02 ± 1.47
	α_1	3.48 ± 0.42	1.94 ± 0.20	116.38 ± 3.22	5.86 ± 1.38
**SASA**	α_2	3.29 ± 0.31	1.84 ± 0.38	116.94 ± 3.89	6.13 ± 0.88
SS = 0.707	α_3	3.03 ± 0.35	2.13 ± 0.24	115.26 ± 3.83	5.66 ± 0.91
DB = 0.553	α_4	2.87 ± 0.53	2.13 ± 0.18	119.38 ± 3.39	5.92 ± 1.17
	β	3.48 ± 0.64	2.42 ± 0.38	125.29 ± 4.21	5.07 ± 1.45
**Complete**		3.40 ± 0.60	2.30 ± 0.40	122.68 ± 5.73	5.30 ± 1.41

†, Silhouette-scores (SS), and Davies-Bouldin Index (DB) were also computed for each clustering algorithm.

Table S9: Different clustered ensembles achieved from utilizing different features for the fingerprints of the dimensionality reduction, and clustering with hierarchical clustering compared for their averaged R_G, RMSD, SASA, and EE_{DIST}.

Feature	Cluster	α-Helices	PPII-Helices	Coils
	α	7.1 ± 1.9	16.6 ± 6.1	49.8 ± 6.5
	$\alpha\beta$	5.6 ± 1.6	15.5 ± 5.6	52.8 ± 5.9
$\mathbf{DR}_{\phi/\psi}$	β_1	0.0 ± 0.0	16.8 ± 6.3	58.7 ± 6.3
	β_2	0.0 ± 0.4	18.0 ± 6.4	57.6 ± 6.8
	β_3	0.0 ± 0.5	18.2 ± 6.7	56.9 ± 6.6
	β_1	0.3 ± 1.3	17.5 ± 6.6	57.4 ± 6.7
	β_2	0.0 ± 0.5	18.0 ± 6.4	57.6 ± 6.7
$\mathbf{DR}_{C\alpha}$	α_3	6.4 ± 2.2	13.3 ± 5.2	50.8 ± 6.1
	α_1	6.8 ± 1.8	18.2 ± 5.8	48.7 ± 6.5
	α_2	7.6 ± 1.7	14.3 ± 5.5	51.6 ± 6.2
	β	0.1 ± 0.7	17.8 ± 6.5	57.5 ± 6.7
	α_1	6.8 ± 2.8	13.3 ± 5.2	51.4 ± 5.4
$\mathbf{DR}_{SASA}$	α_2	7.4 ± 1.5	16.5 ± 6.5	50.4 ± 7.2
	α_3	5.9 ± 2.3	16.9 ± 5.7	49.8 ± 6.6
	α_4	7.1 ± 1.1	17.9 ± 5.4	48.9 ± 6.1
**Complete**		2.1 ± 3.4	17.4 ± 6.4	55.3 ± 7.5

The choice of input features for the dimensionality reduction is of vital consideration. Underinclusion of features results in non-robust ensembles, while overinclusion of features (as seen in Figure S29) may overcomplicate the dimensionality reduction and add noise.

S6 Silhouette Score and Davies Bouldin Index.

The Silhouette Score (SS) and the Davies-Bouldin Index (DBI) are commonly used metrics to evaluate the quality of clustering results in machine learning. These metrics give numerical scores that can help determine the effectiveness of a clustering algorithm. The Silhouette Score measures the cohesion and separation of the clusters in a dataset, as seen in Equation 3. It calculates the similarity of each instance to its own cluster and compares it to the similarity to other clusters. The score ranges from -1 to 1, where higher scores indicate that the clusters are well-defined and distinct. A score of 0 suggests that an instance could belong to multiple clusters, while a negative score indicates that an instance may have been assigned to the wrong cluster. On the other hand, the Davies-Bouldin Index evaluates the overall quality of clustering results by considering both the within-cluster variance and the separation between clusters. It quantifies the average separability of each cluster from its nearest counterpart. Lower DBI values indicate better clustering solutions, with 0 representing perfect clustering and higher values indicating poor separation and high variance within clusters. In general, the Silhouette Score focuses on how well individual instances are clustered, while the Davies-Bouldin Index considers the overall quality of the clustering solution. The Silhouette Score is useful for evaluating the placement of specific instances within clusters, while the DBI provides a comprehensive assessment of the clustering results.

$$s(i) = \begin{cases} 1 - a(i)/b(i), & \text{if } a(i) < b(i) \\ 0, & \text{if } a(i) = b(i) \\ b(i)/a(i) - 1, & \text{if } a(i) > b(i) \end{cases} \tag{3}$$

S7 Integrated Silhouette Score.

An integrated silhouette score (SS_{INT}) is a metric that combines silhouette scores computed in both high dimensional and low dimensional spaces to assess the reliability of clustering. Unlike the normal silhouette score, which is typically calculated based on distances in either the original high dimensional space or the reduced low dimensional space, SS_{INT} considers both to provide a more comprehensive evaluation of clustering efficiency. The normal silhouette score for a data point is calculated by:

Where $a(i)$ is the average distance of the ith data point to the other points in the same cluster, intracluster distance, and $b(i)$ is the minimum average distance of the ith data point to the points in a different cluster, thus measuring the nearest cluster that the point is not a part of the intercluster distance. A high value close to 1 indicates good clustering, with values closer to 0 indicating poor clustering, and negative values suggesting incorrect clustering configurations. The SS_{INT} (SS_{low} * SS_{high}) combines the silhouette scores calculated in the low dimensional space (SS_{low}) and the high dimensional space (SS_{high}), thereby adding value to the estimated clustering efficiency in terms of reliability. This approach further ensures that the clustering is not only effective in the latent space but also reflects true proximities and separation in the original high dimensional space, addressing potential distortions or losses of information due to the dimensionality reduction process.

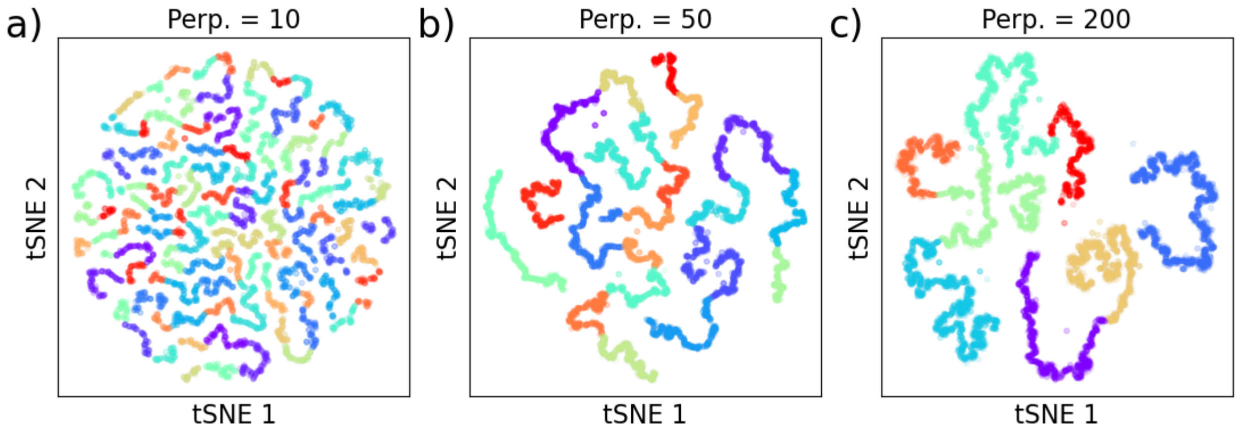

Figure S27: Dimensionality reduction of the MAP2C/C trajectory using ϕ/ψ features and nonlinear DR technique (tSNE) with the perplexity (a) 10, (b) 50, and (c) 200. Hierarchical clustering was implemented to generate (a) 100, (b) 20, and (c) 8 clusters representing the need to match cluster size to the respective perplexity.

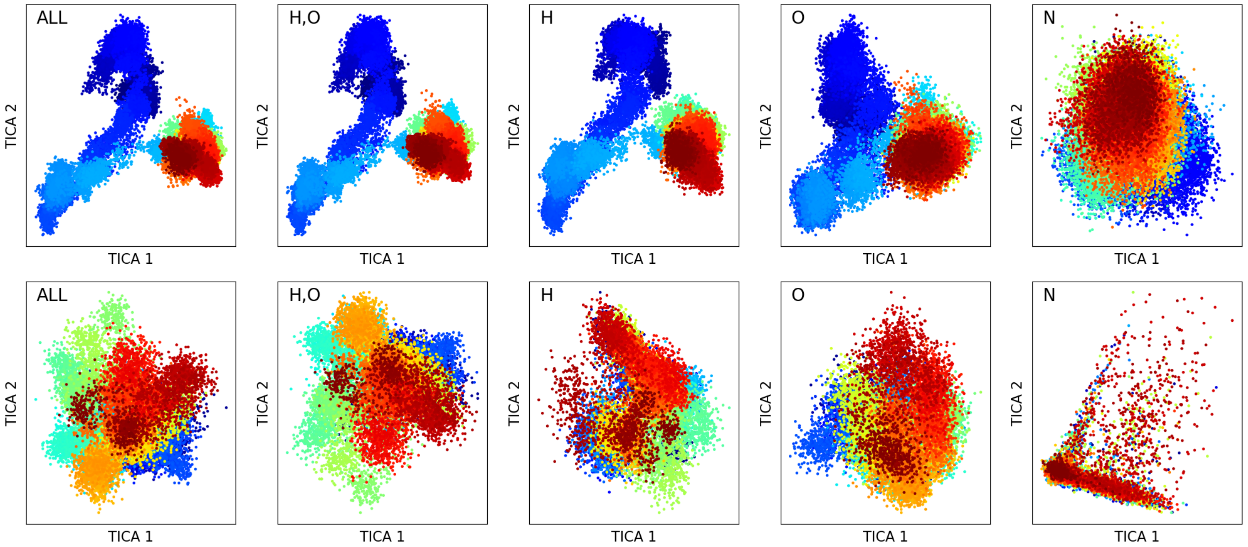

Figure S28: Dimensionality reductions on the SASA for different atom types and the distribution in the latent space of MAP2C/C (top), and the ordered protein GB3 (bottom).

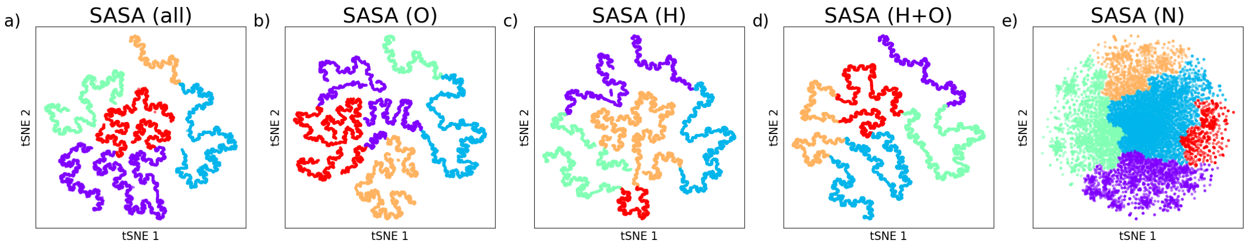

Figure S29: Dimensionality reductions on MAP2C/C using the feature SASA with different atom types and the distribution in the latent space upon clustering.

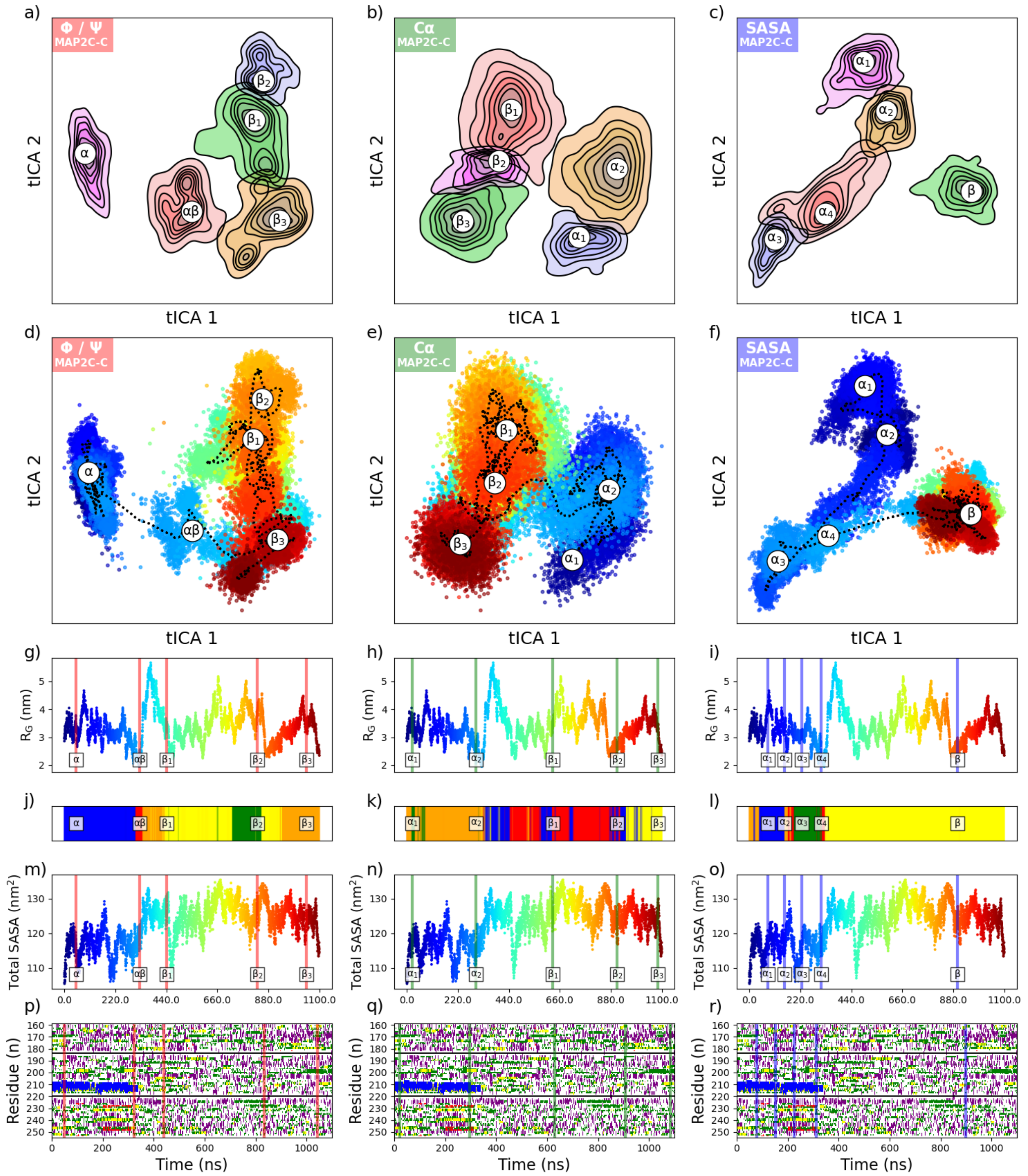

Figure S30: Dimensionality reduction and clustering of the MAP2C/C trajectory using linear technique tICA, and input features ϕ and ψ (a), α-carbon distances and angles (b), and SASA (c), with comparative rolling averages (d-f), labeled according to their observed secondary structures, as seen in the DSSP plots (p-r). Radius of gyration (g-i) and total SASA (m-o) are included for comparison for the time dependence of the clusters (j-l).

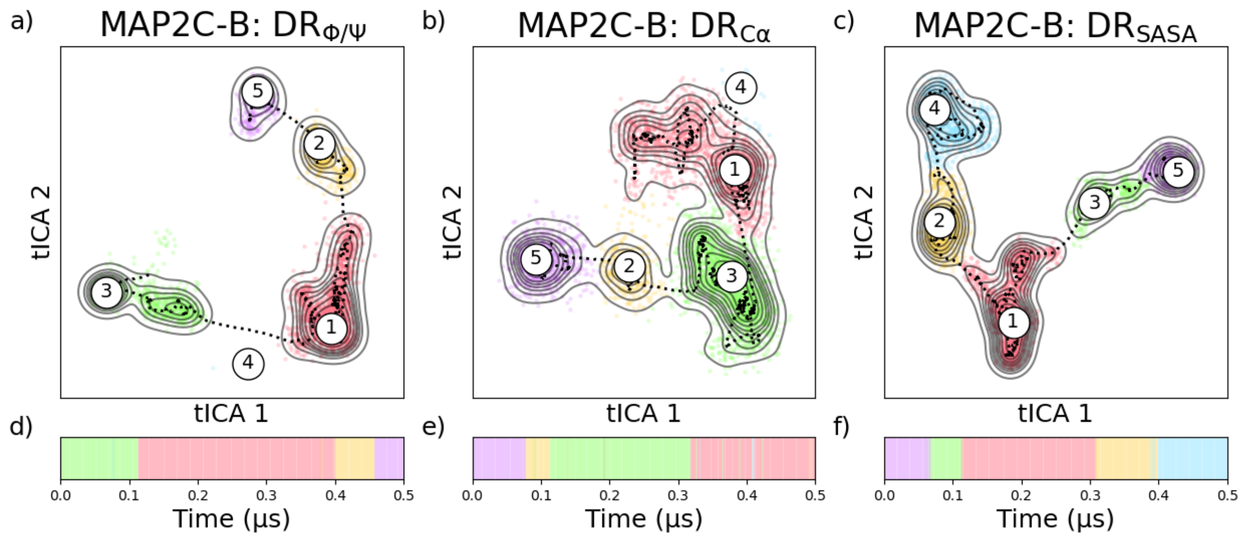

Figure S31: Dimensionally reduced landscapes of MAP2C/B clustered into five labeled clusters (a-c), and the respect place within the trajectory (d-f) using different input features; ϕ/ψ (a), α carbons distances and angles (b), and SASA (c).

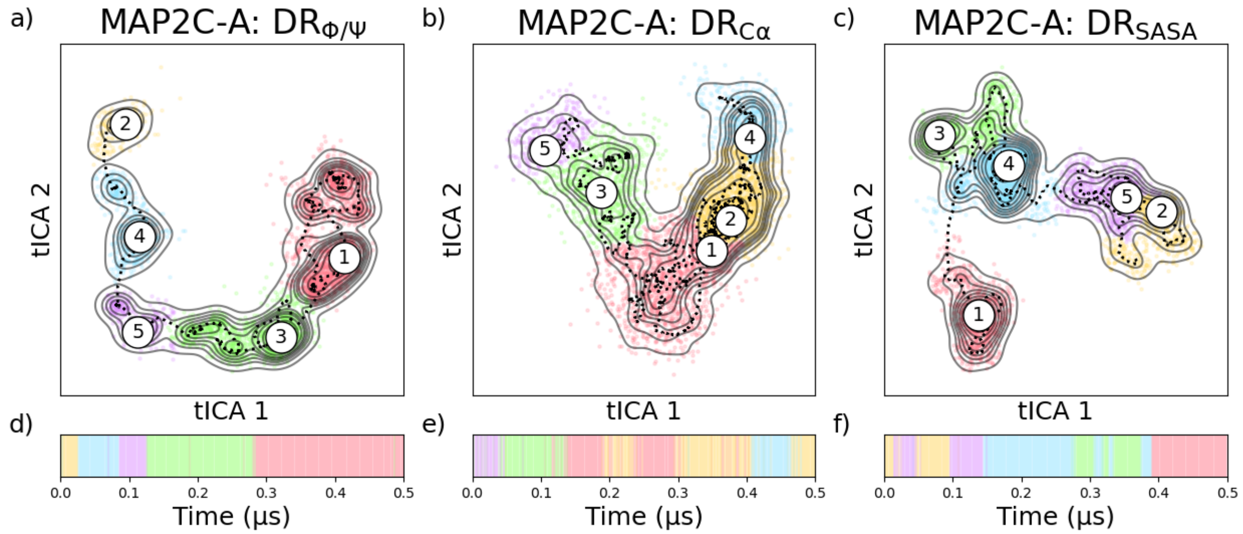

Figure S32: Dimensionally reduced landscapes of MAP2C/A clustered into five labeled clusters (a-c), and the respect place within the trajectory (d-f) using different input features; ϕ/ψ (a), α carbons distances and angles (b), and SASA (c).

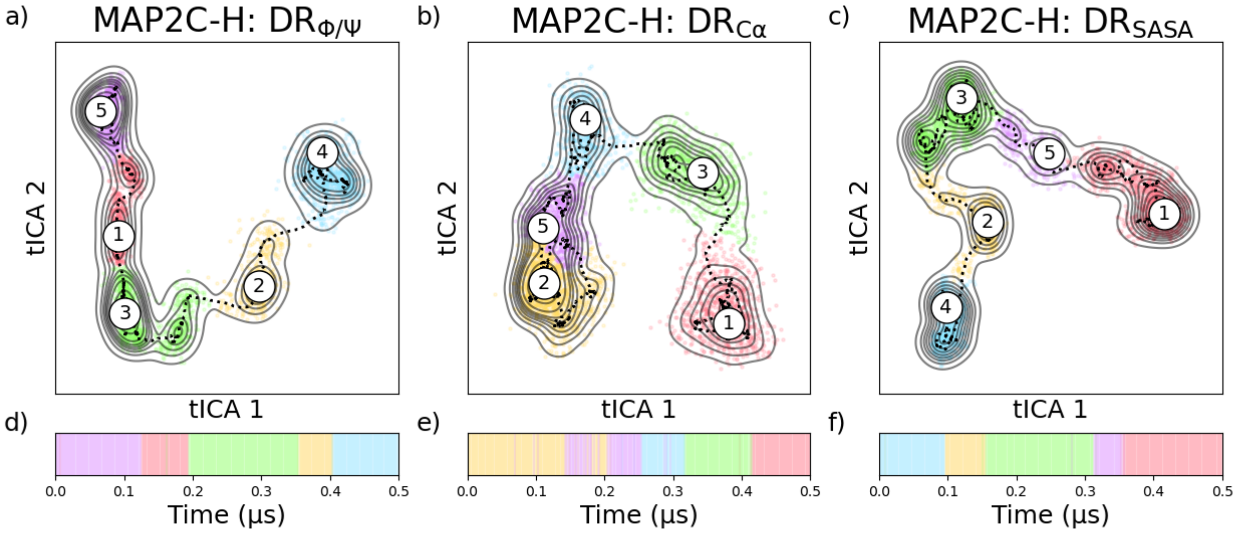

Figure S33: Dimensionally reduced landscapes of MAP2C/H clustered into five labeled clusters (a-c), and the respect place within the trajectory (d-f) using different input features; ϕ/ψ (a), α carbons distances and angles (b), and SASA (c).

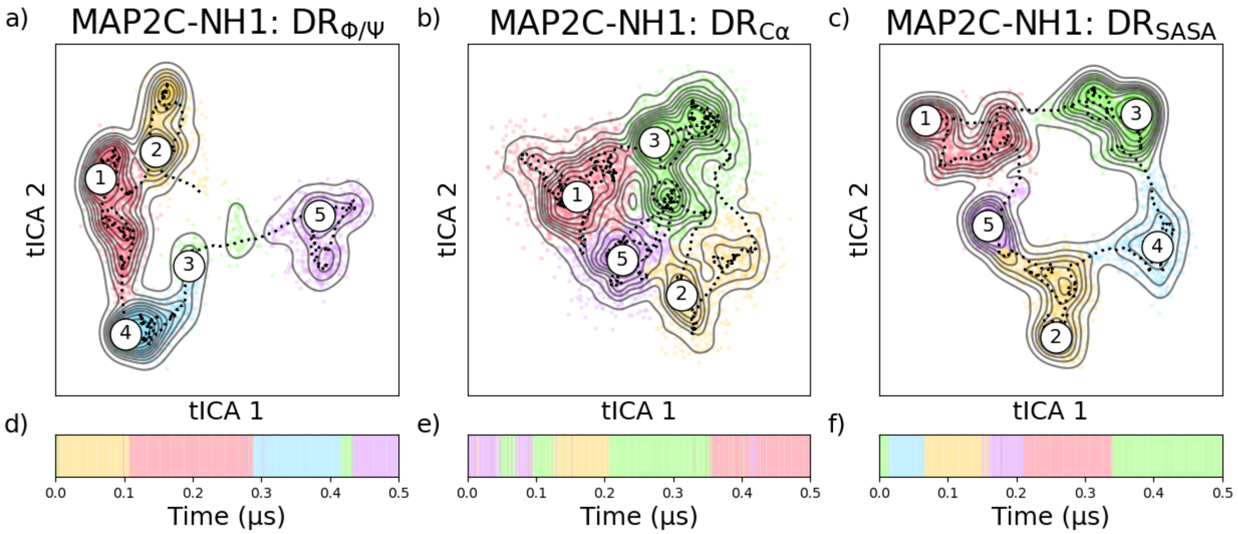

Figure S34: Dimensionally reduced landscapes of MAP2C/NH1 clustered into five labeled clusters (a-c), and the respect place within the trajectory (d-f) using different input features; ϕ/ψ (a), α carbons distances and angles (b), and SASA (c).

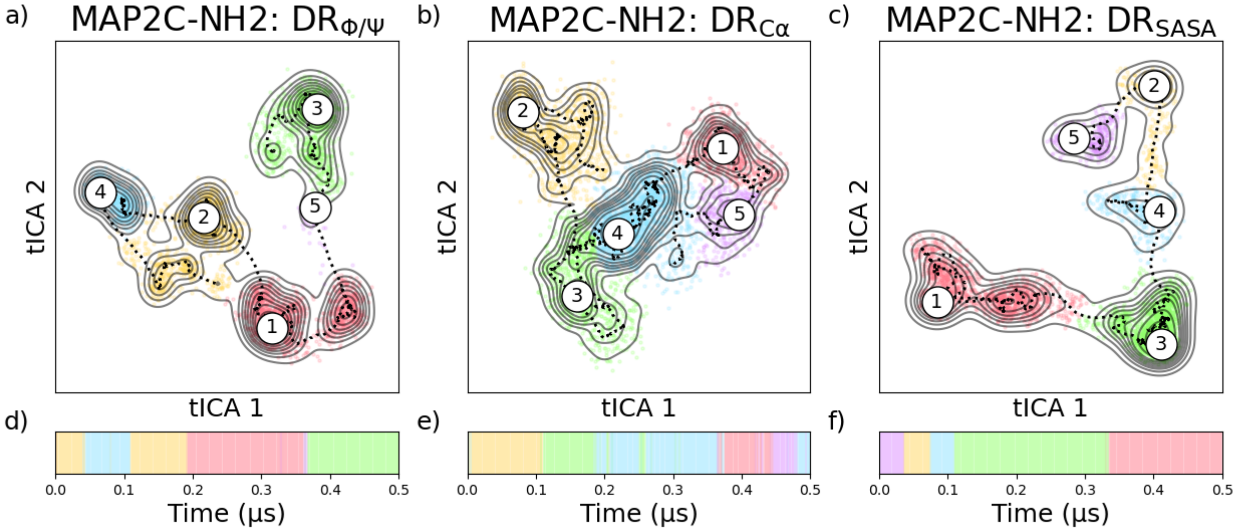

Figure S35: Dimensionally reduced landscapes of MAP2C/NH2 clustered into five labeled clusters (a-c), and the respect place within the trajectory (d-f) using different input features; ϕ/ψ (a), α carbons distances and angles (b), and SASA (c).

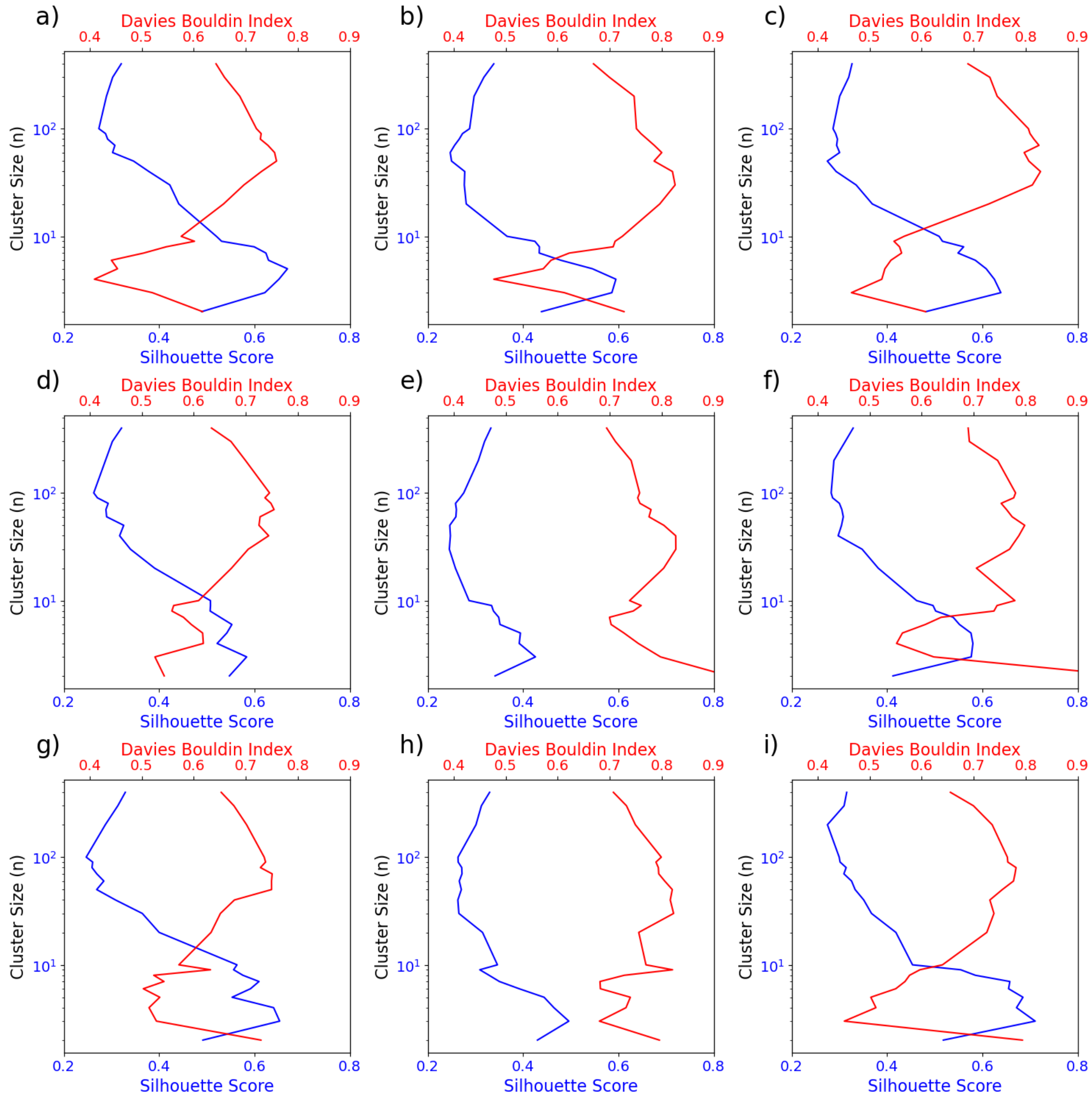

Figure S36: Silhouette Score and Davies Bouldin Index computed for different cluster sizes of the tICA reduced landscapes using the input features ϕ and ψ (a,d,g), α-carbon distances and angles (b,e,h), and SASA (c,f,i) in the non-phosphorylated MAP2C trajectories; MAP2C/H (a-c), MAP2C/NH1 (d-f), and MAP2C/NH2 (g-i).

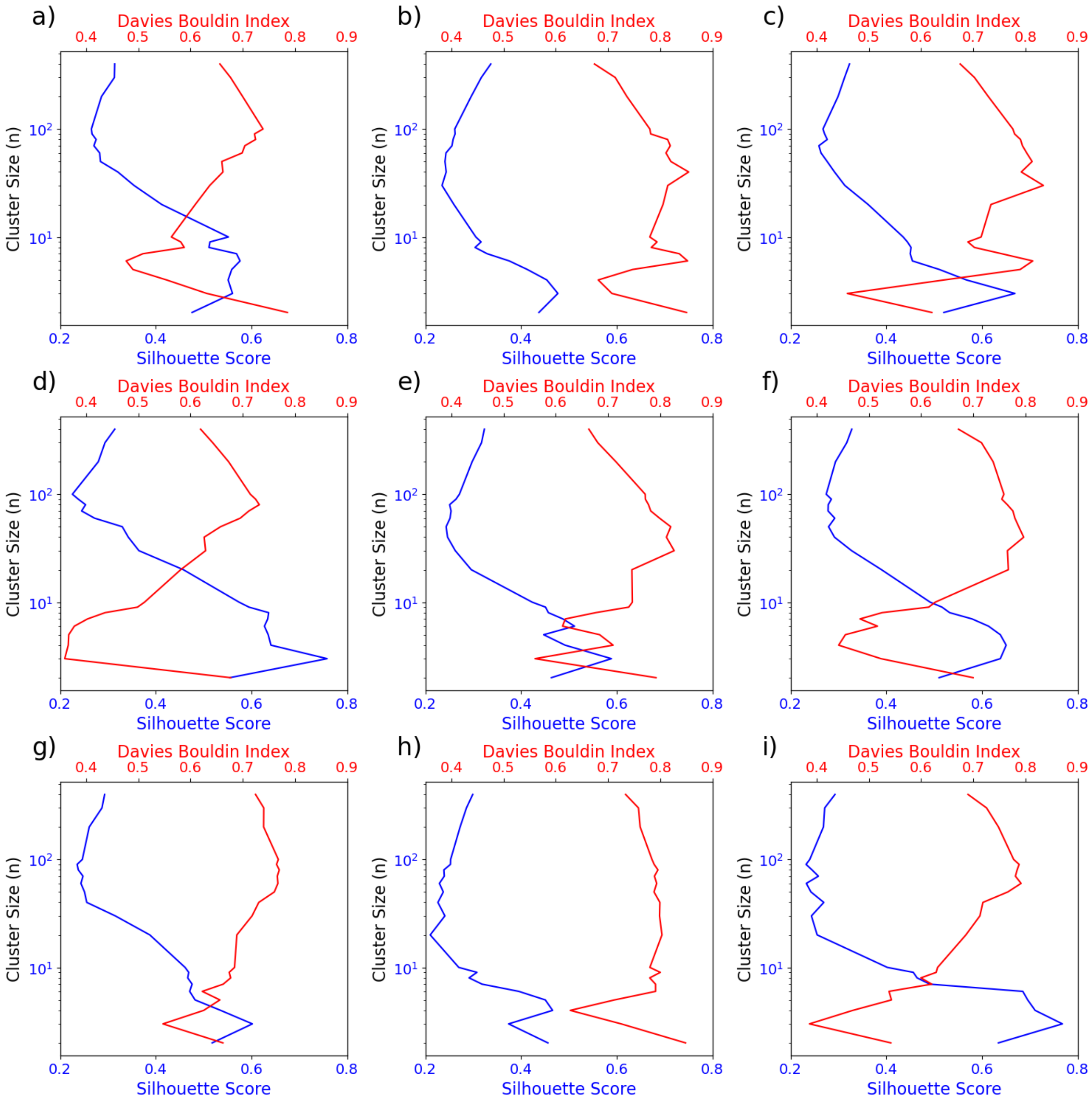

Figure S37: Silhouette Score and Davies Bouldin Index computed for different cluster sizes of the tICA reduced landscapes using the input features ϕ and ψ (a,d,g), α-carbon distances and angles (b,e,h), and SASA (c,f,i) in the phosphorylated MAP2C trajectories; MAP2C/A (a-c), MAP2C/B (d-f), and MAP2C/C (g-i).

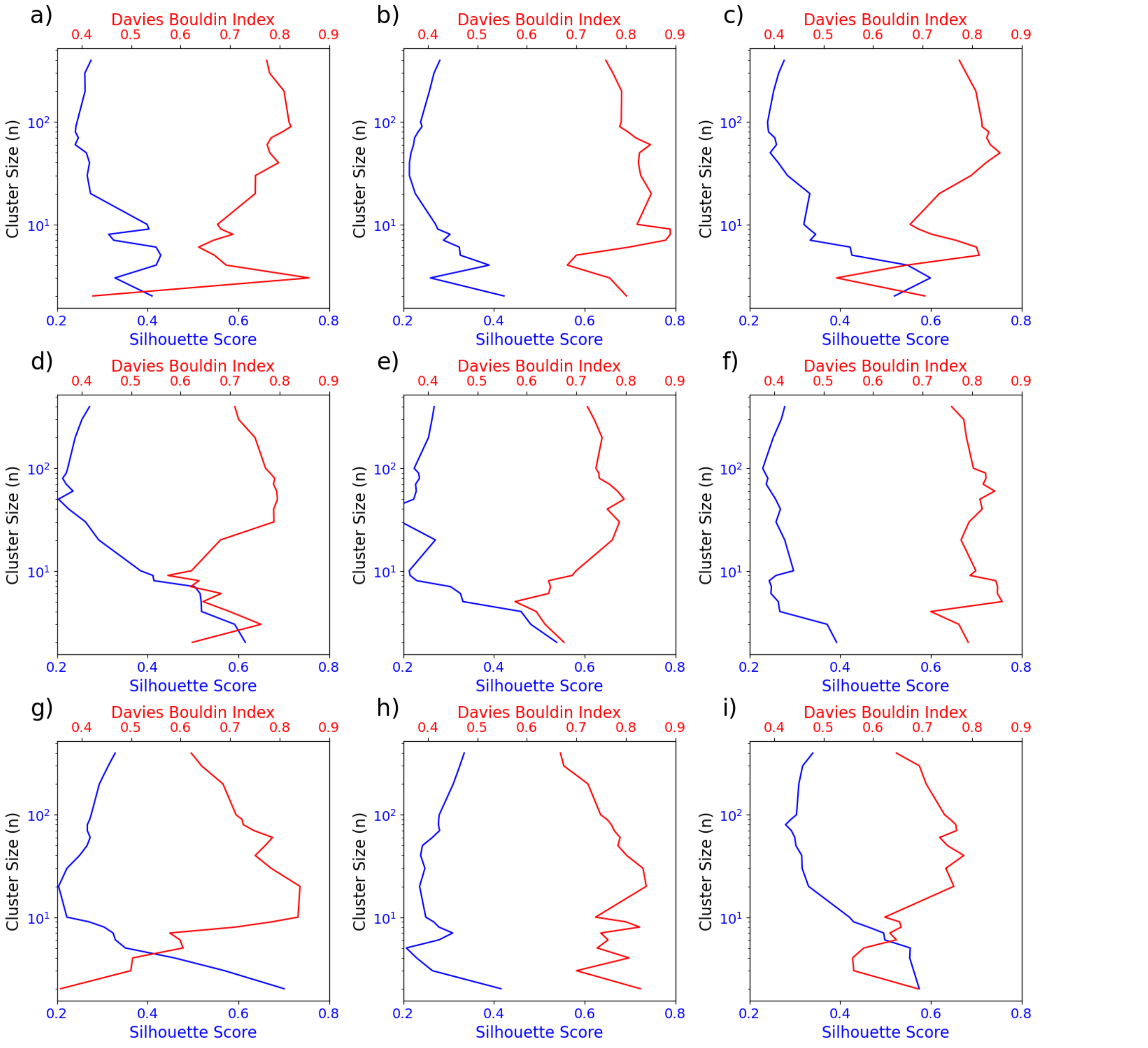

Figure S38: Silhouette Score and Davies Bouldin Index computed for different cluster sizes of the tICA reduced landscapes using the input features ϕ and ψ (a,d,g), α-carbon distances and angles (b,e,h), and SASA (c,f,i) in hTH1 (a-c), GB3 (d-f), and UBIQ (g-i).

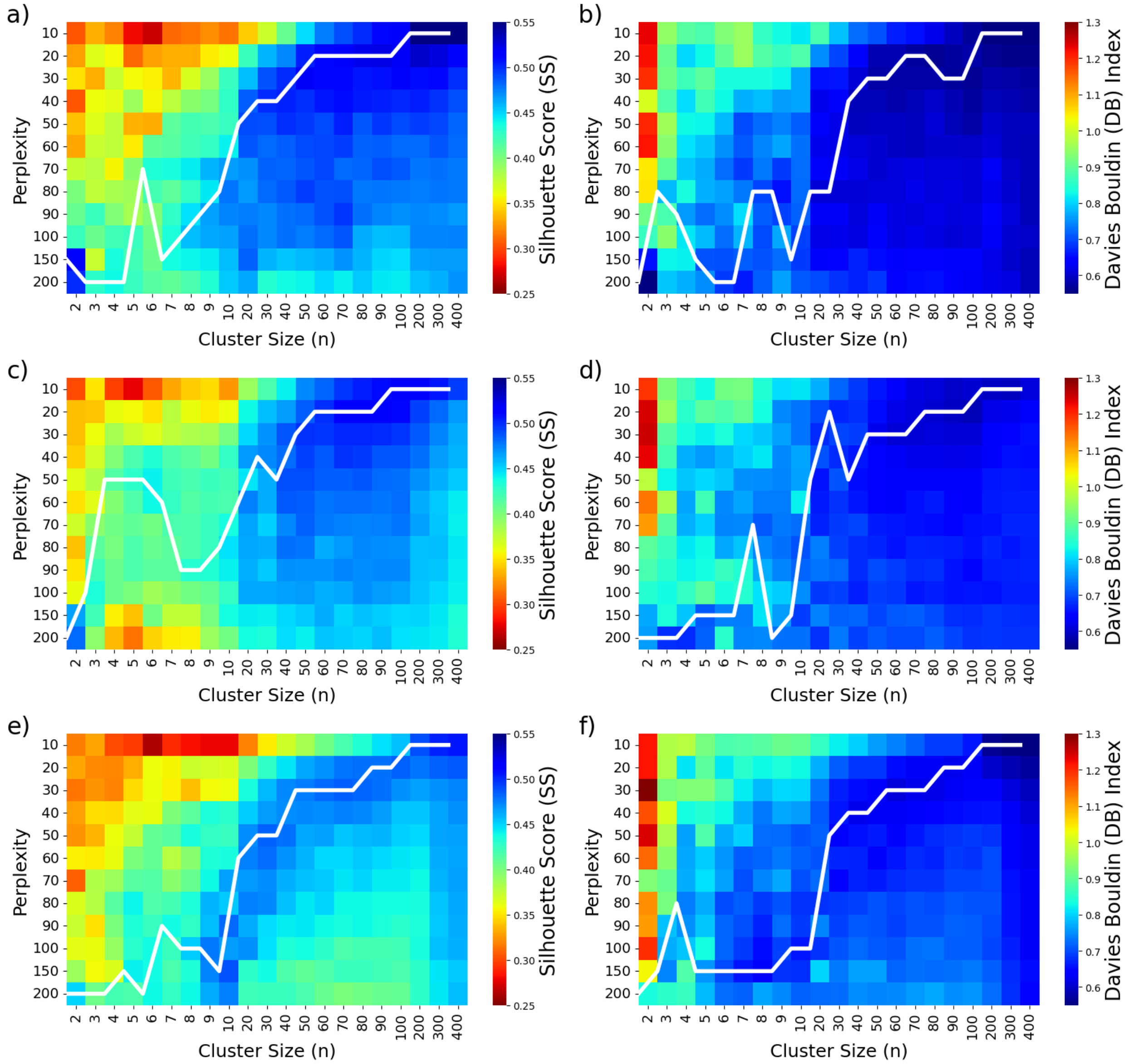

Figure S39: Silhouette Scores (a,c,e) and Davies Bouldin Indexes (b,d,f) computed for different cluster sizes and perplexities in tSNE dimensionality reduction and clustering using input features ϕ and ψ (a/b), α-carbon distances and angles (c/d), and SASA (e/f) in the MAP2C/A trajectory.

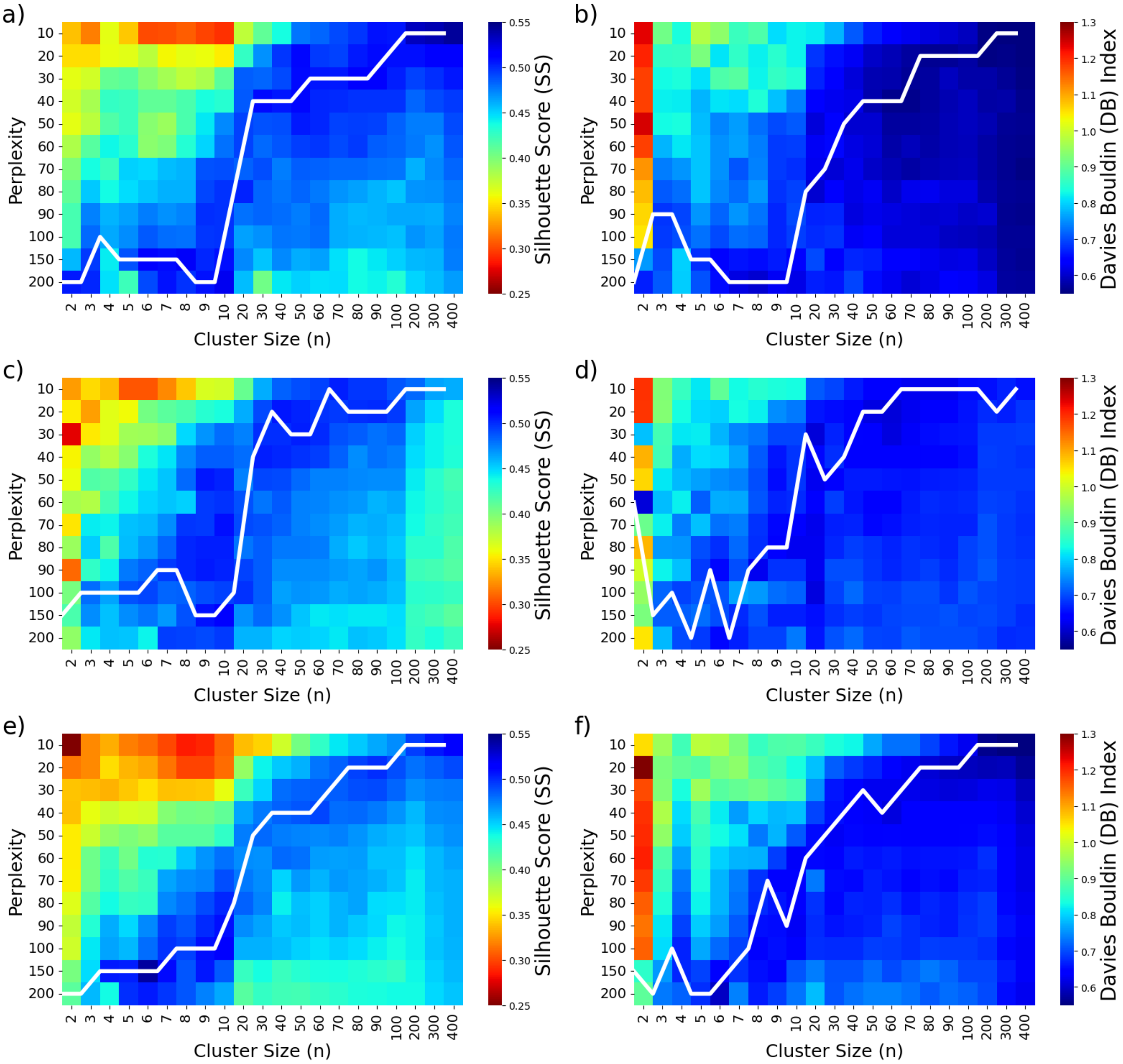

Figure S40: Silhouette Scores (a,c,e) and Davies Bouldin Indexes (b,d,f) computed for different cluster sizes and perplexities in tSNE dimensionality reduction and clustering using input features ϕ and ψ (a/b), α-carbon distances and angles (c/d), and SASA (e/f) in the MAP2C/B trajectory.

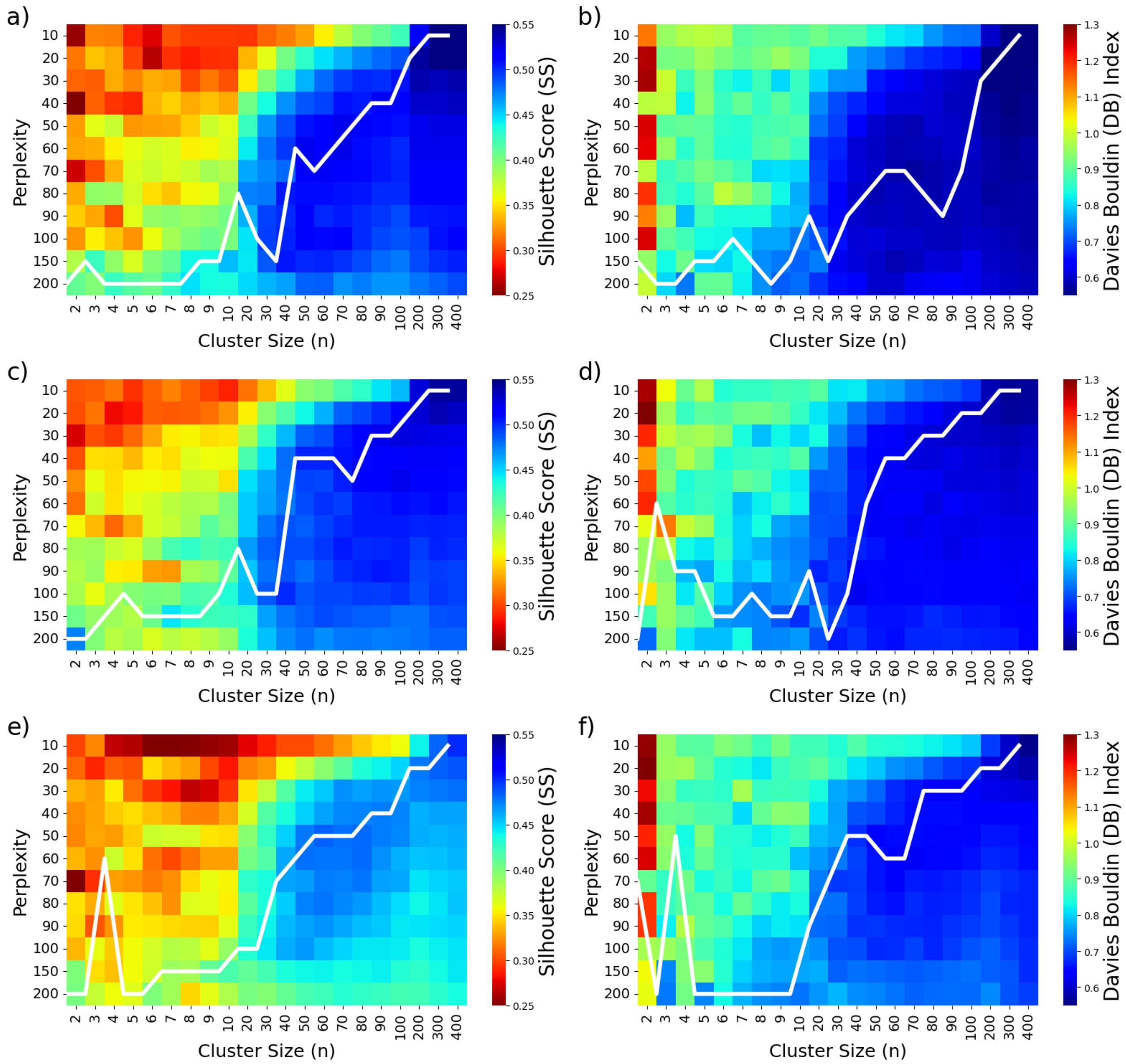

Figure S41: Silhouette Scores (a,c,e) and Davies Bouldin Indexes (b,d,f) computed for different cluster sizes and perplexities in tSNE dimensionality reduction and clustering using input features ϕ and ψ (a/b), α-carbon distances and angles (c/d), and SASA (e/f) in the MAP2C/C trajectory.

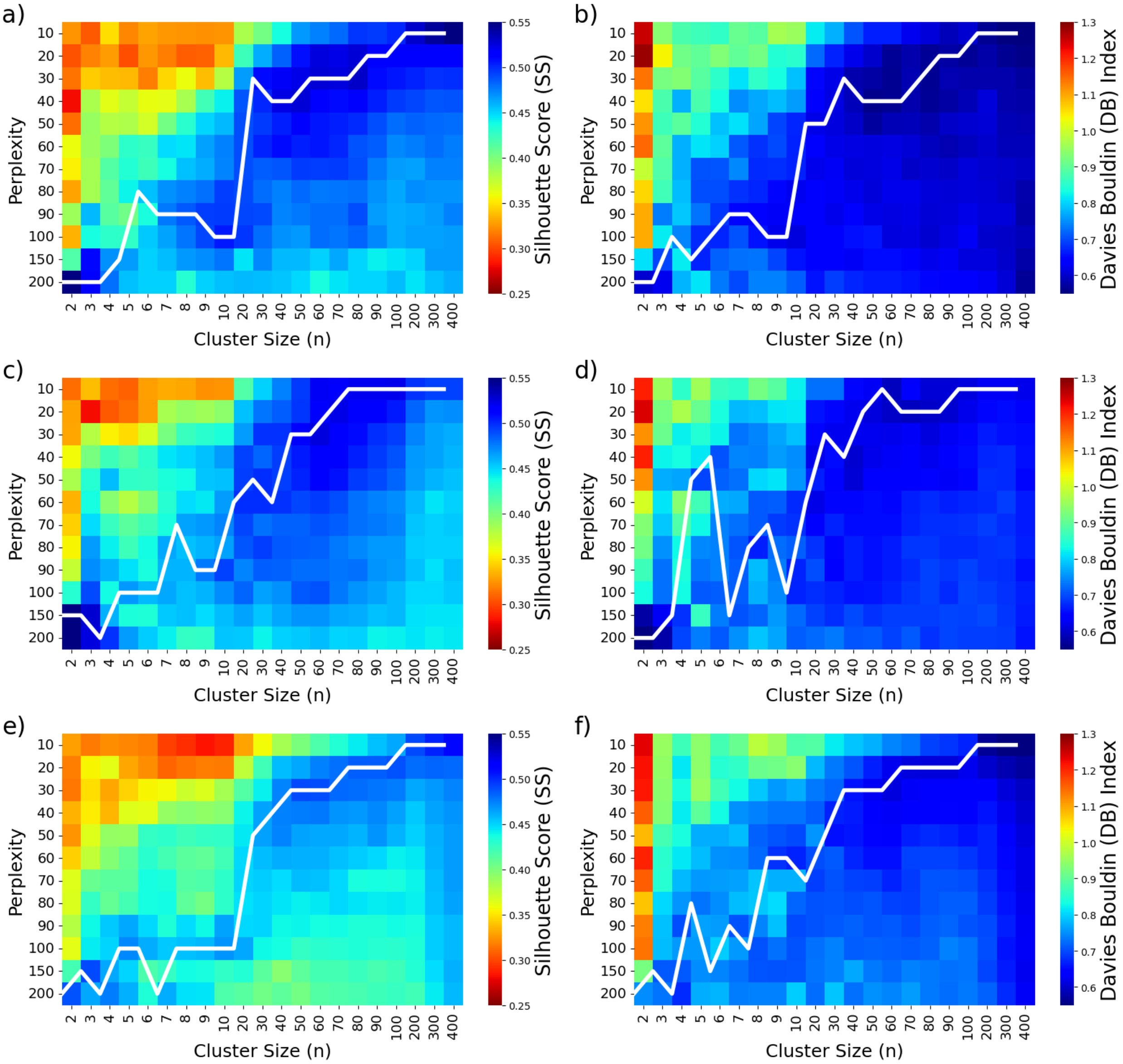

Figure S42: Silhouette Scores (a,c,e) and Davies Bouldin Indexes (b,d,f) computed for different cluster sizes and perplexities in tSNE dimensionality reduction and clustering using input features ϕ and ψ (a/b), α-carbon distances and angles (c/d), and SASA (e/f) in the MAP2C/H trajectory.

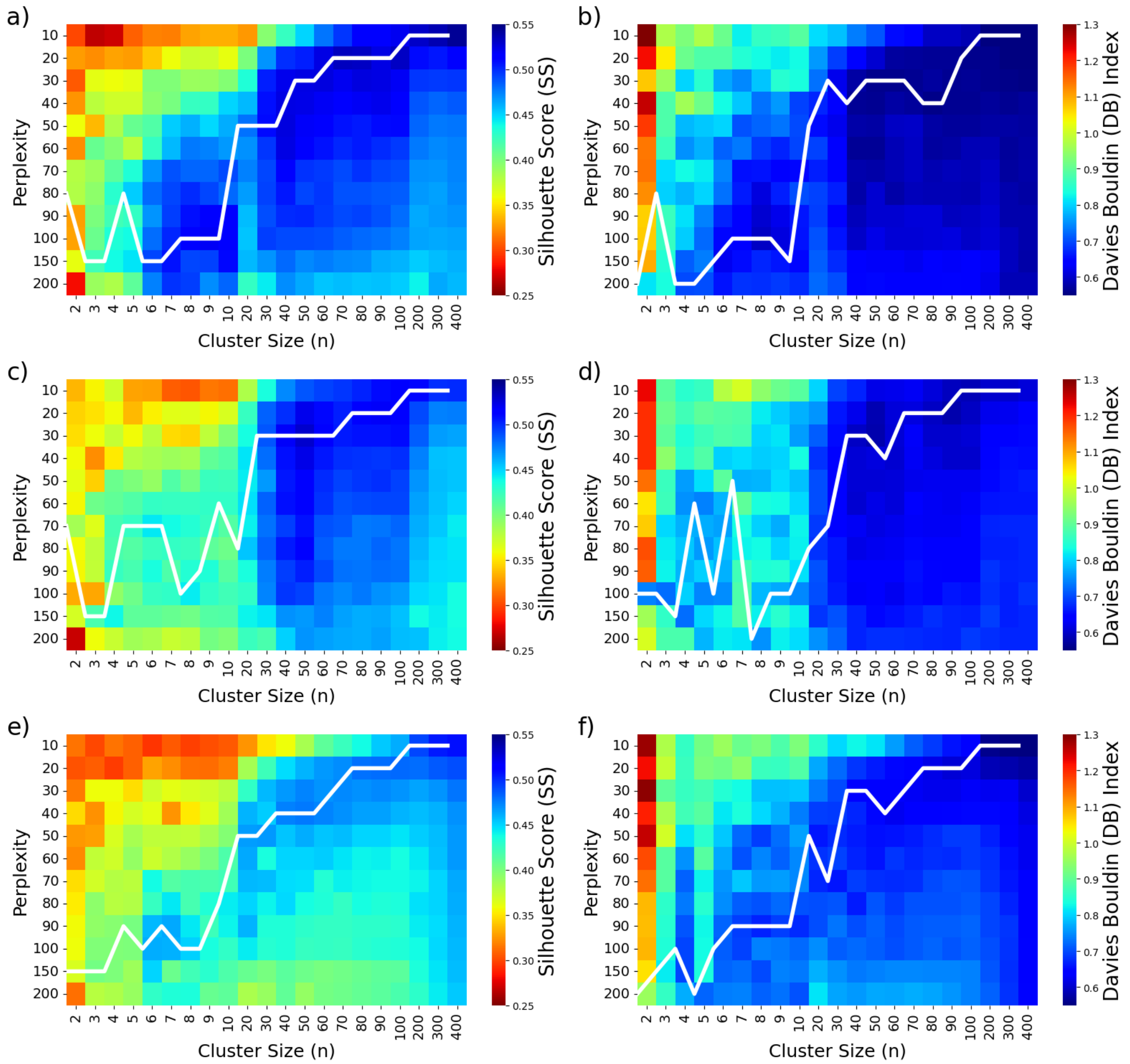

Figure S43: Silhouette Scores (a,c,e) and Davies Bouldin Indexes (b,d,f) computed for different cluster sizes and perplexities in tSNE dimensionality reduction and clustering using input features ϕ and ψ (a/b), α-carbon distances and angles (c/d), and SASA (e/f) in the MAP2C/NH1 trajectory.

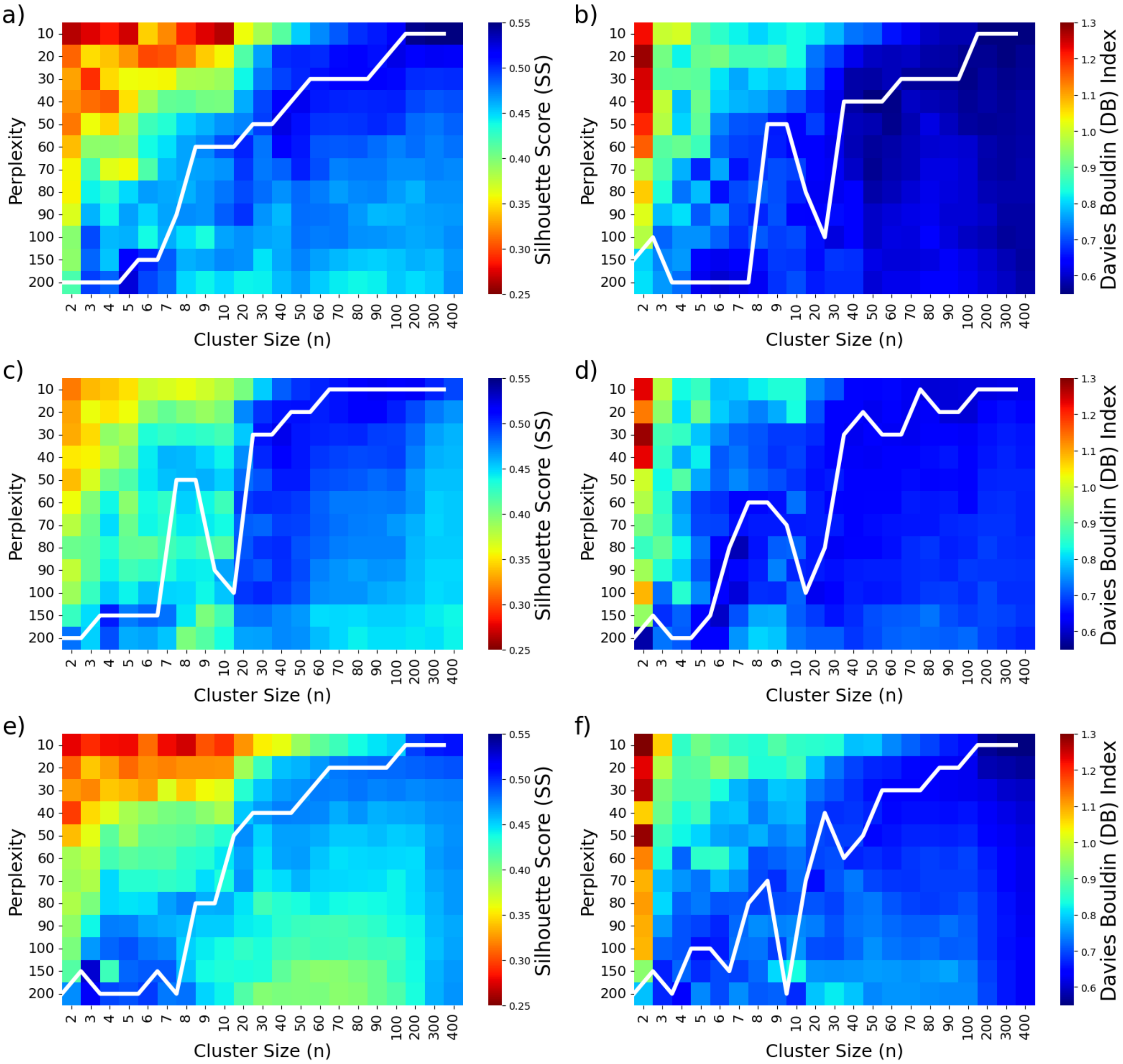

Figure S44: Silhouette Scores (a,c,e) and Davies Bouldin Indexes (b,d,f) computed for different cluster sizes and perplexities in tSNE dimensionality reduction and clustering using input features ϕ and ψ (a/b), α-carbon distances and angles (c/d), and SASA (e/f) in the MAP2C/NH2 trajectory.

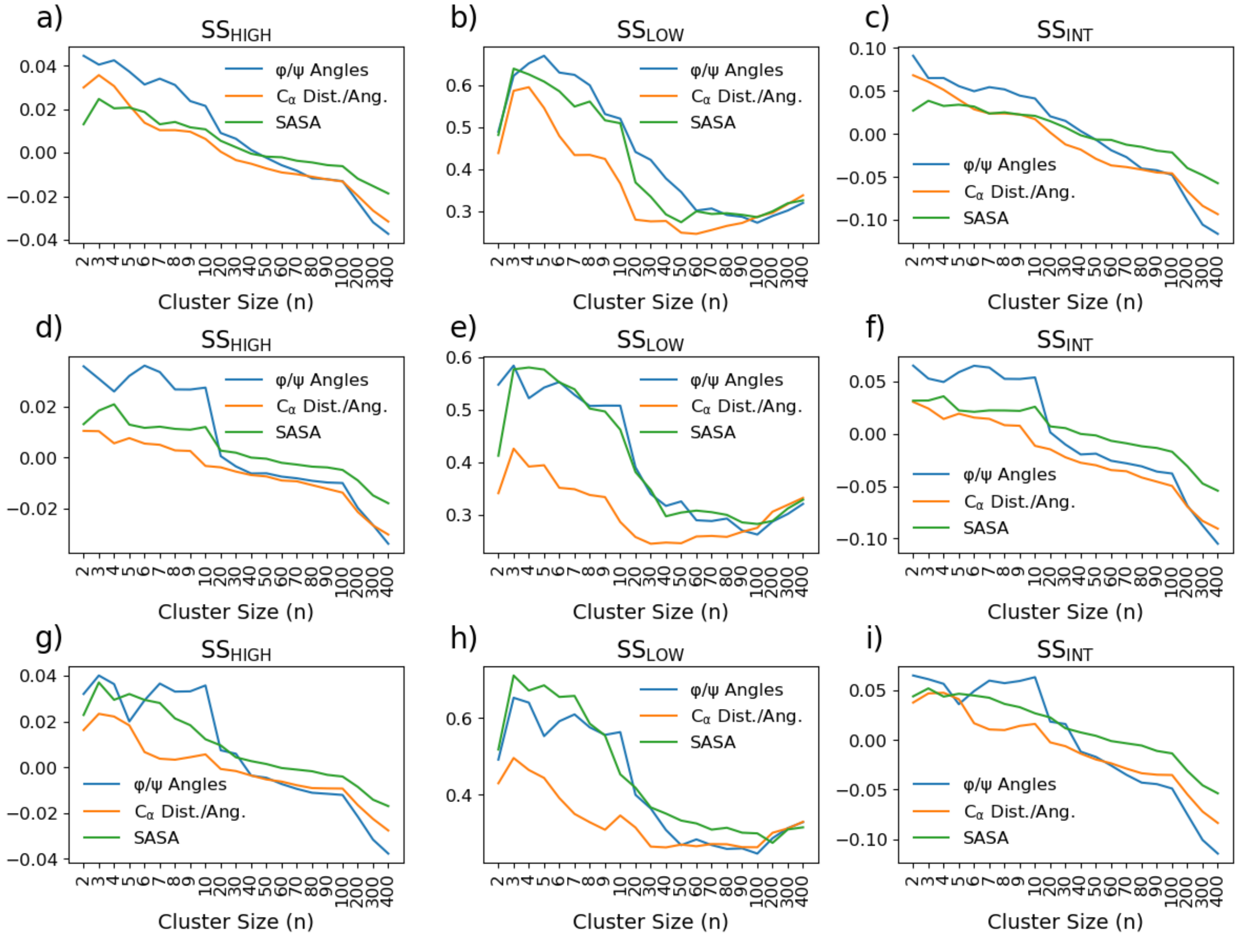

Figure S45: Silhouette Scores calculated from high (a,d,g), low (b,e,h), and integrated (c,f,i) for different cluster sizes of the tICA reduced landscapes using the input features ϕ and ψ, α-carbon distances and angles, and SASA in the non-phosphorylated MAP2C trajectories; MAP2C/H (a-c), MAP2C/NH1 (d-f), and MAP2C/NH2 (g-i).

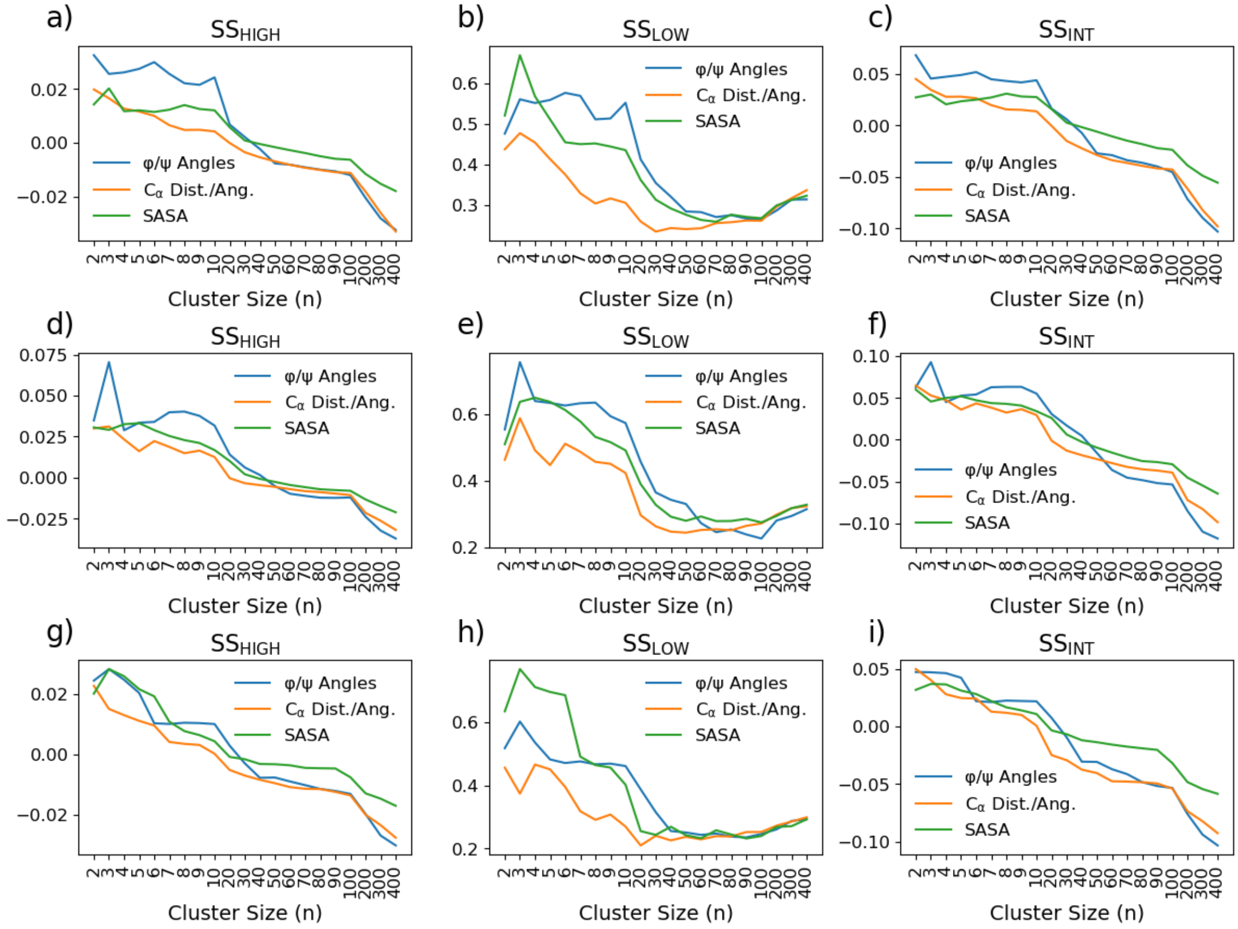

Figure S46: Silhouette Scores calculated from high (a,d,g), low (b,e,h), and integrated (c,f,i) for different cluster sizes of the tICA reduced landscapes using the input features ϕ and ψ, α-carbon distances and angles, and SASA in the phosphorylated MAP2C trajectories; MAP2C/A (a-c), MAP2C/B (d-f), and MAP2C/C (g-i).

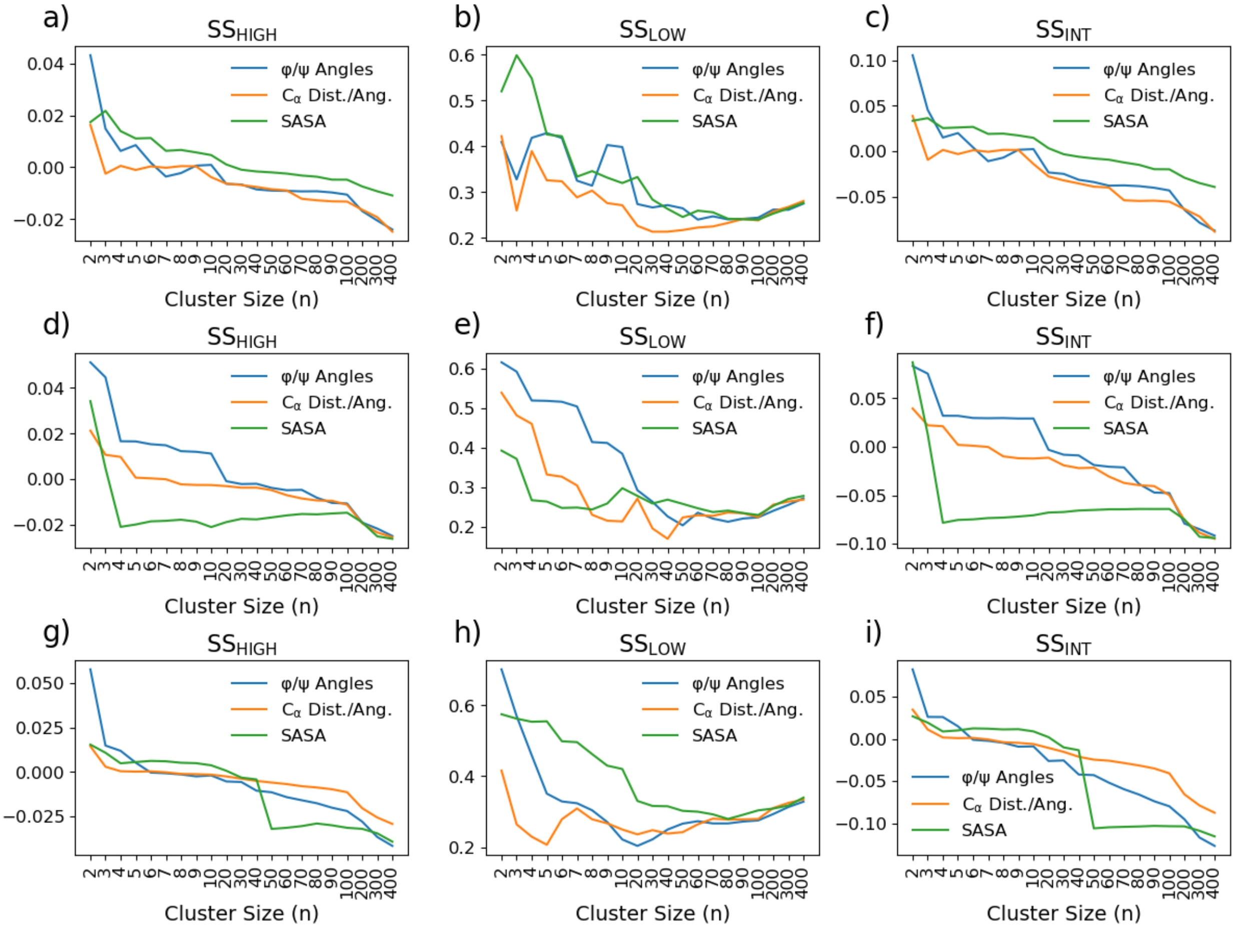

Figure S47: Silhouette Scores calculated from high (a,d,g), low (b,e,h), and integrated (c,f,i) for different cluster sizes of the tICA reduced landscapes using the input features ϕ and ψ, α-carbon distances and angles, and SASA in hTH1 (a-c), GB3 (d-f), and UBIQ (g-i).

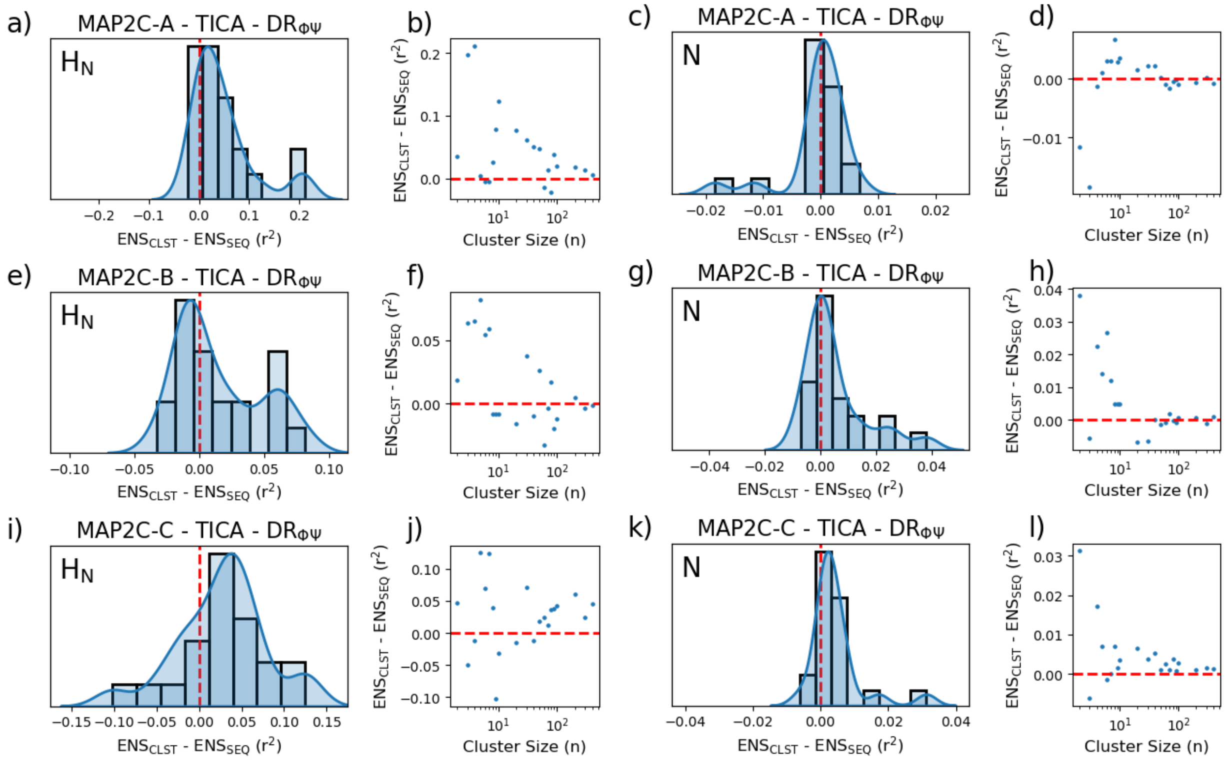

Figure S48: Distribution of the difference between the clustered ensemble's (CE) through tICA DR and clustering using ϕ/ψ angles for input features and equally sized sequential ensemble's (SE) agreement with experimental chemical shifts in the phosphorylated MAP2C trajectories represented as a histogram and kernel-density estimation, and the difference in performance of the CEs as a function of cluster size.

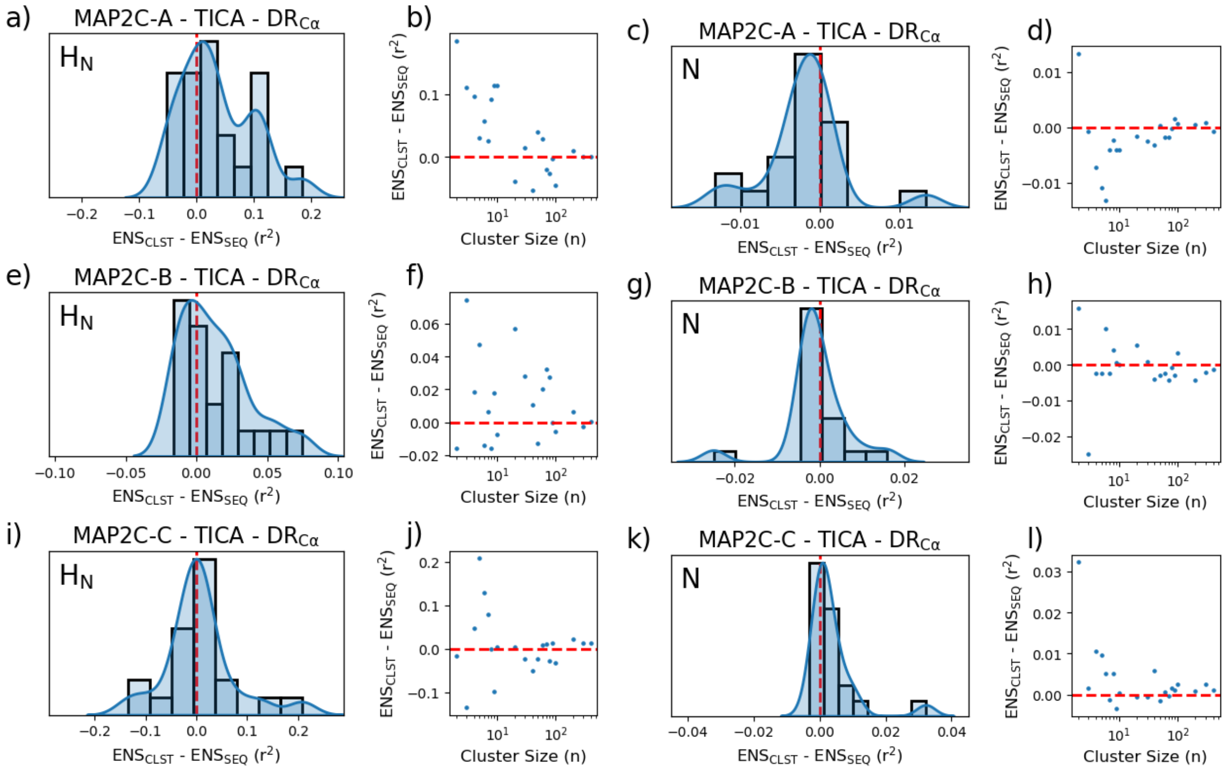

Figure S49: Distribution of the difference between the clustered ensemble's (CE) through tICA DR and clustering using α-carbon distances and angles for input features and equally sized sequential ensemble's (SE) agreement with experimental chemical shifts in the phosphorylated MAP2C trajectories represented as a histogram and kernel-density estimation, and the difference in performance of the CEs as a function of cluster size.

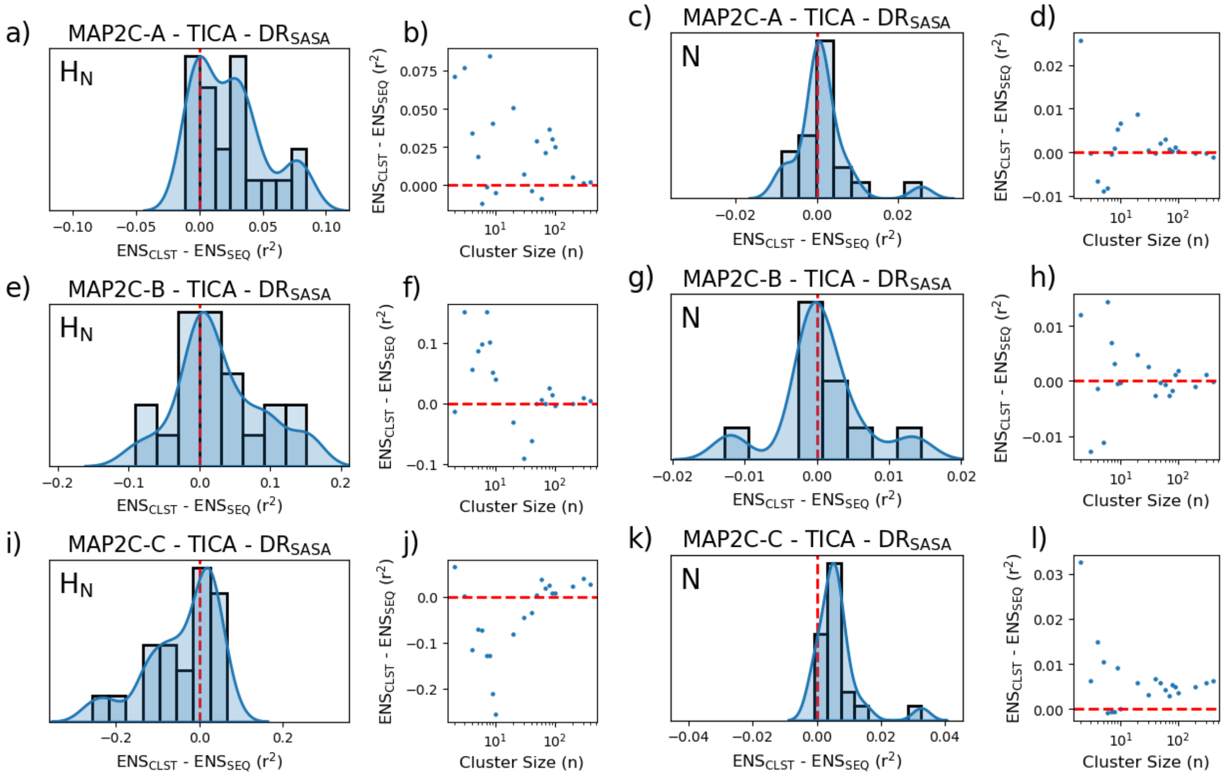

Figure S50: Distribution of the difference between the clustered ensemble's (CE) through tICA DR and clustering using SASA for input features and equally sized sequential ensemble's (SE) agreement with experimental chemical shifts in the phosphorylated MAP2C trajectories represented as a histogram and kernel-density estimation, and the difference in performance of the CEs as a function of cluster size.

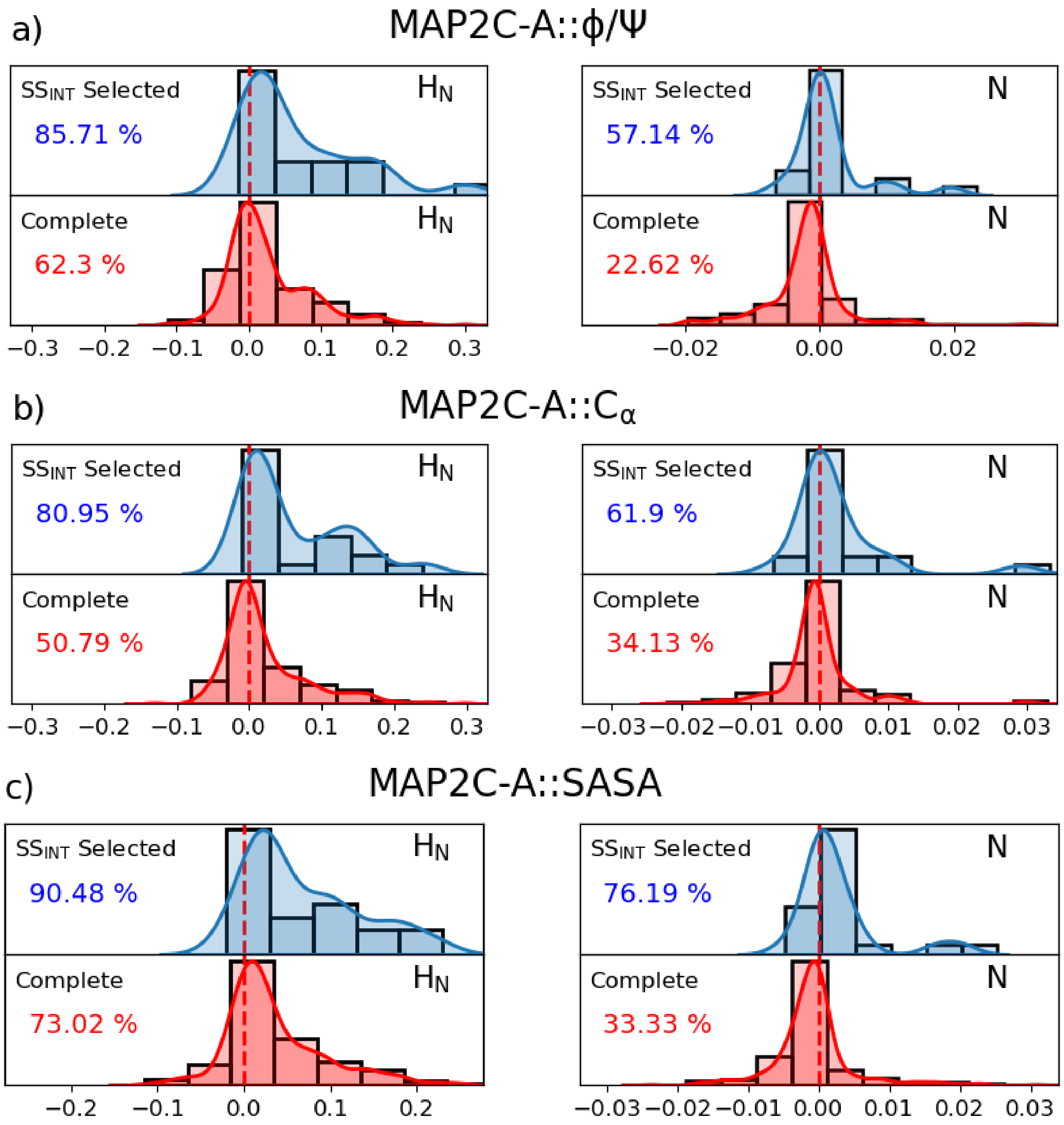

Figure S51: Distribution of clustered ensembles (CEs) generated from MAP2C/A through tSNE DR and clustering using (a) ϕ/ψ dihedrals, (b) α-carbon distances and angles, and (c) SASA as input features as a function of their performance in relation to equally sized sequential ensembles (SEs). Recorded in blue is the collection of ensembles selected using the SS_{INT} scanning protocol in blue, and the original complete set of CEs in red. X-axes represent r^2 values difference between clustered and sequential.

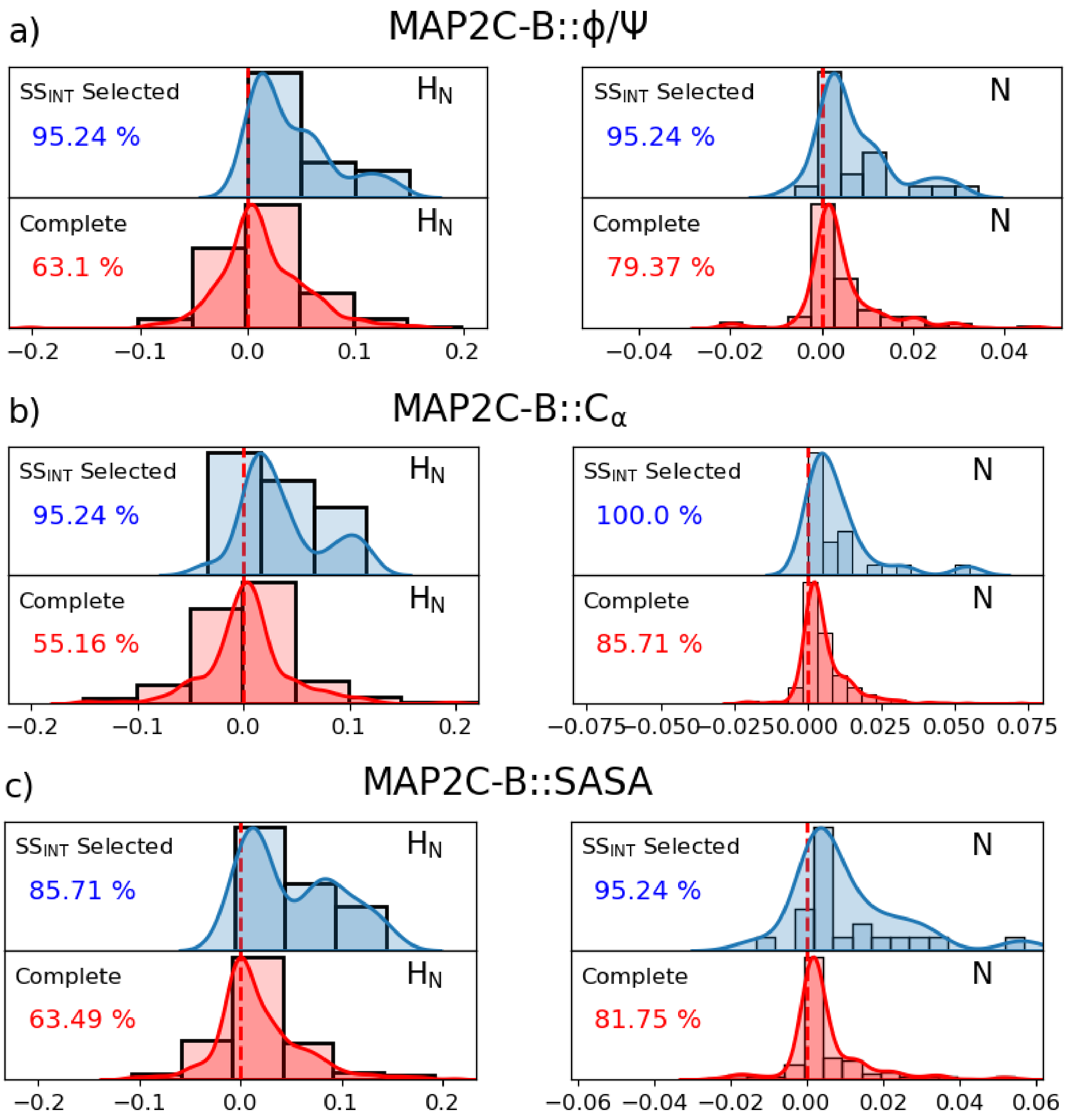

Figure S52: Distribution of clustered ensembles (CEs) generated from MAP2C/B through tSNE DR and clustering using (a) ϕ/ψ dihedrals, (b) α-carbon distances and angles, and (c) SASA as input features as a function of their performance in relation to equally sized sequential ensembles (SEs). Recorded in blue is the collection of ensembles selected using the SS_{INT} scanning protocol in blue, and the original complete set of CEs in red. X-axes represent r^2 values difference between clustered and sequential.

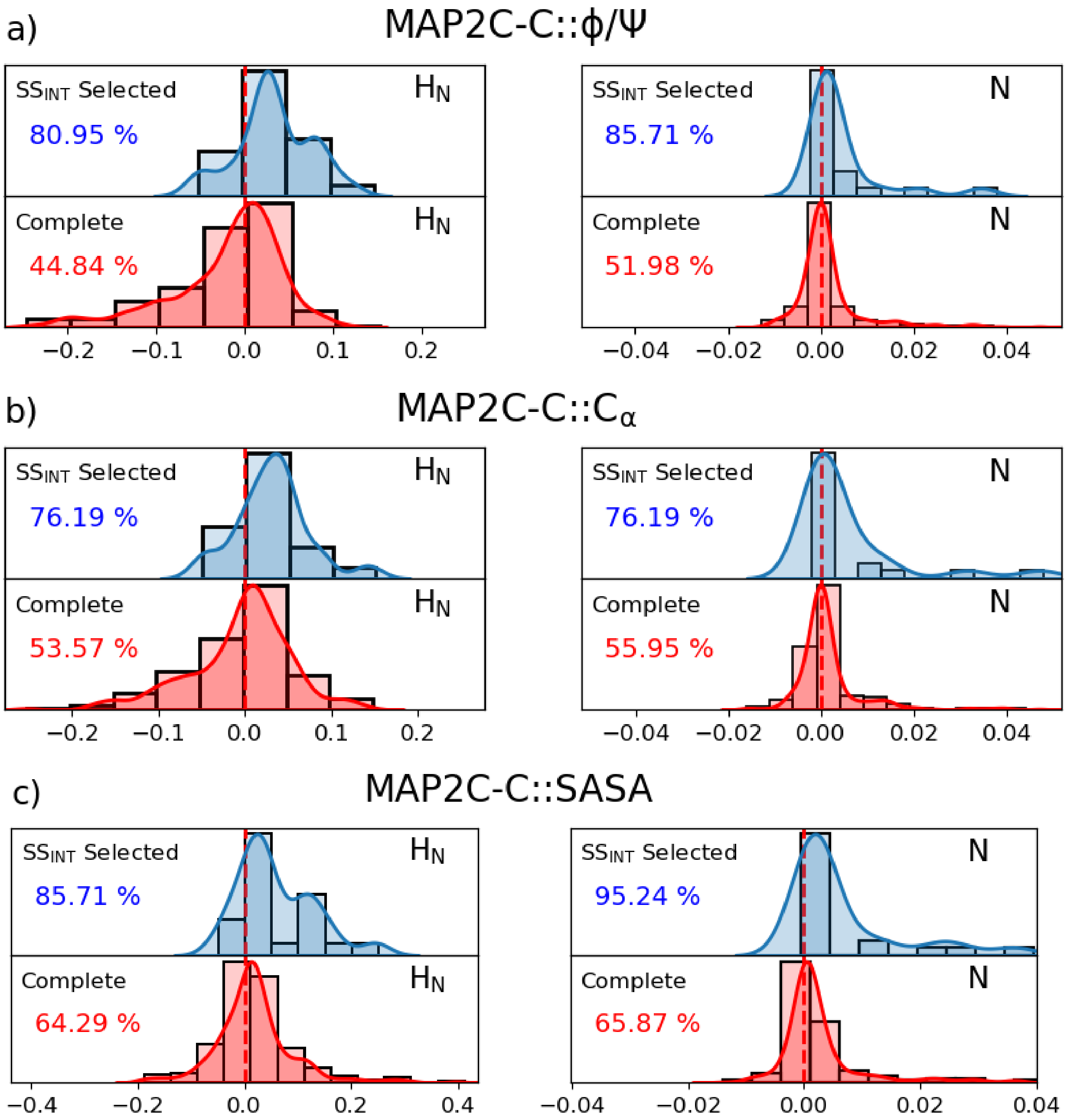

Figure S53: Distribution of clustered ensembles (CEs) generated from MAP2C/C through tSNE DR and clustering using (a) ϕ/ψ dihedrals, (b) α-carbon distances and angles, and (c) SASA as input features as a function of their performance in relation to equally sized sequential ensembles (SEs). Recorded in blue is the collection of ensembles selected using the SS_{INT} scanning protocol in blue, and the original complete set of CEs in red. X-axes represent r^2 values difference between clustered and sequential.

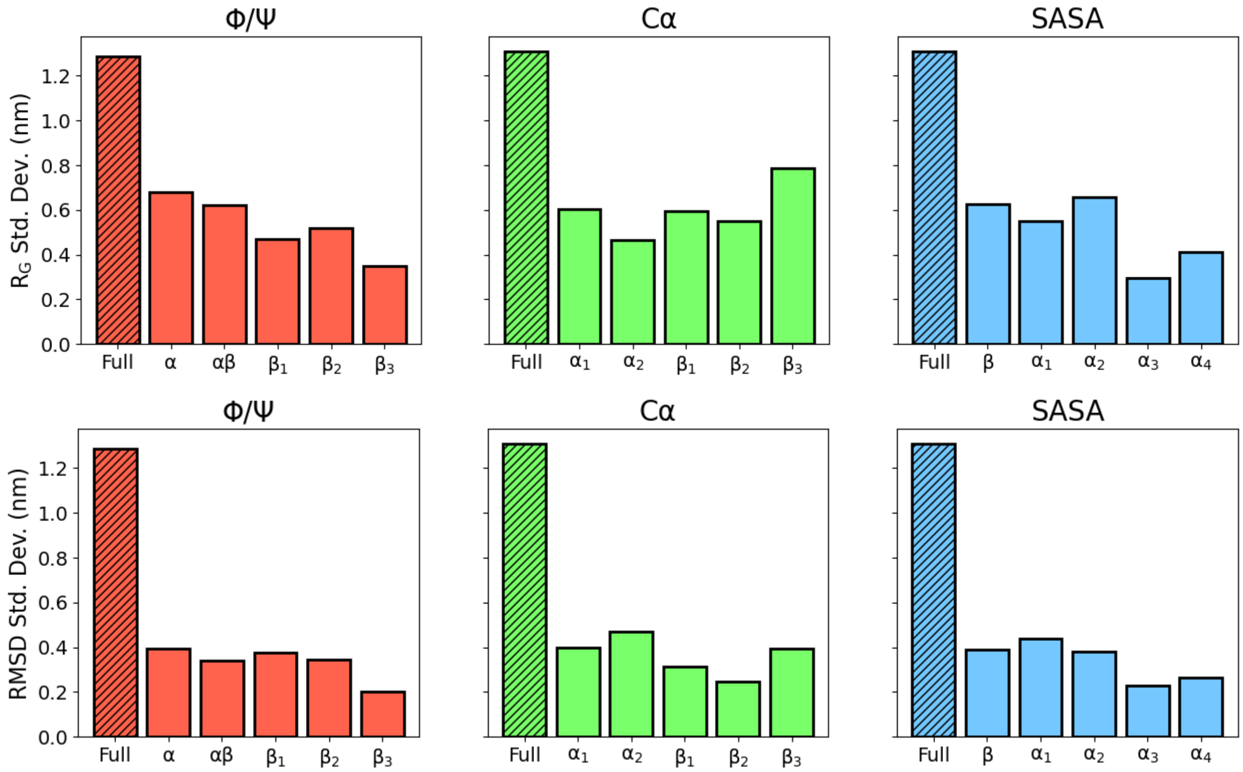

Figure S54: Comparisons of the variability (standard deviation) from the root-mean-squared deviation (top) and radius of gyration (bottom) of the ensembles generated from the MAP2C/C using ϕ/ψ (left), alpha carbon distance/angles (middle), and solvent-accessible surface area (right) after clustering (N=5). Shaded columns represent the global properties from the complete trajectory.

References

(1) Kvålseth, T. O. Cautionary Note about R 2. *Am. Stat.* **1985**, *39*, 279–285.

(2) Felli, I. C.; Bermel, W.; Pierattelli, R. Exclusively Heteronuclear NMR Experiments for the Investigation of Intrinsically Disordered Proteins: Focusing on Proline Residues. *Magn. Reson.* **2021**, *2*, 511–522.

(3) Plucarová, J.; Jansen, S.; Narasimhan, S.; Laníková, A.; Lewitzky, M.; Feller, S. M.; Žídek, L. Specific Phosphorylation of Microtubule-Associated Protein 2C by Extracellular Signal–Regulated Kinase Reduces Interactions at Its Pro-Rich Regions. *J. Biol. Chem.* **2022**, *298*, 102384.
